# Broad-Spectrum Virus Trapping with Heparan Sulfate-Modified DNA Origami Shells

**DOI:** 10.1021/acsnano.1c11328

**Published:** 2022-11-02

**Authors:** Alba Monferrer, Jessica A. Kretzmann, Christian Sigl, Pia Sapelza, Anna Liedl, Barbara Wittmann, Hendrik Dietz

**Affiliations:** † Laboratory for Biomolecular Nanotechnology. Department of Physics, Technical University of Munich, Am Coulombwall 4a, 85748 Garching, Germany; ‡ Munich Institute of Biomedical Engineering, Technical University of Munich, Boltzmannstraße 11, 85748 Garching, Germany

**Keywords:** DNA origami, heparan sulfate, heparin, antiviral, broad-spectrum, virus-like particles

## Abstract

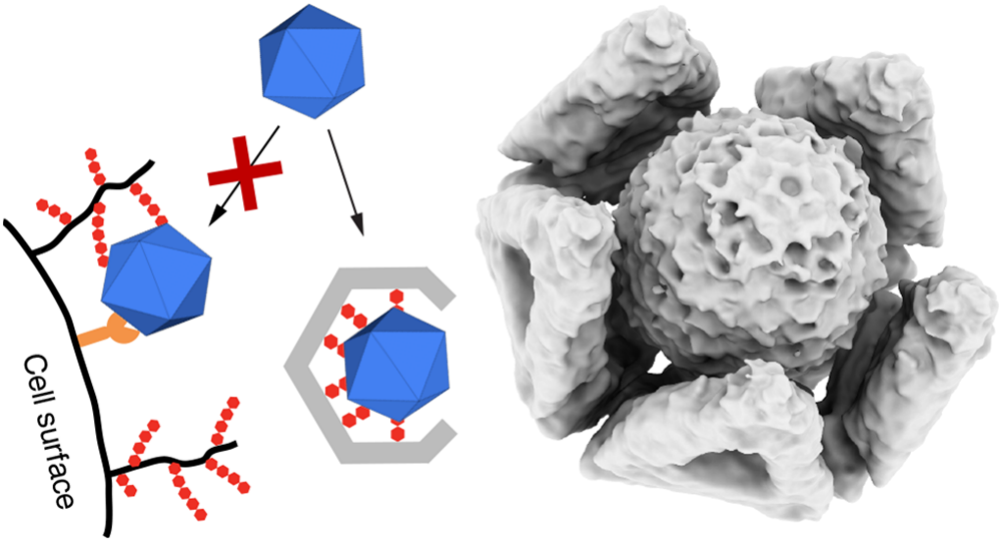

Effective broadband antiviral platforms that can act on existing viruses and viruses yet to emerge are not available, creating a need to explore treatment strategies beyond the trodden paths. Here, we report virus-encapsulating DNA origami shells that achieve broadband virus trapping properties by exploiting avidity and a widespread background affinity of viruses to heparan sulfate proteoglycans (HSPG). With a calibrated density of heparin and heparan sulfate (HS) derivatives crafted to the interior of DNA origami shells, we could encapsulate adeno, adeno-associated, chikungunya, dengue, human papilloma, noro, polio, rubella, and SARS-CoV-2 viruses or virus-like particles, in one and the same HS-functionalized shell system. Additional virus-type-specific binders were not needed for the trapping. Depending on the relative dimensions of shell to virus particles, multiple virus particles may be trapped per shell, and multiple shells can cover the surface of clusters of virus particles. The steric occlusion provided by the heparan sulfate-coated DNA origami shells can prevent viruses from further interactions with receptors, possibly including those found on cell surfaces.

At present, there are over 200 known viral-vector borne human diseases, of which few are treatable with current antiviral drugs.^1^ In the search for effective antiviral therapies, neutralizing antibodies are increasingly being considered for treating acute viral infections.^2,3^ Antiviral antibodies often achieve their virus-neutralizing function by blocking the interactions that viruses undergo with specific receptors on the surface of host cells. However, antibodies are prone to losing their function due to mutational drift, they take time to develop, and they will typically be effective for only one virus or virus serotype at a time.

In addition to targeting specific receptors, many viruses also interact with polysaccharides such as heparan sulfate proteoglycans (HSPG)^4,5^ located on the surface of mammalian cells (Figure 1A, left panel). Interactions of viruses with HSPG are conserved across virus families. The specificity of binding of HS to viruses depends on the distribution and conformation of β-d-GlcA and α-l-IdoA residues, relative amounts of N-acetyl or N-sulfate groups in the GlcN moiety, and on the relative amounts and the position of O-sulfation of the uronic acid and GlcN units.^6^ Specific sequences of disaccharides can favor the interaction of the molecule with certain proteins and not to others.^4,6^

**Figure 1:**
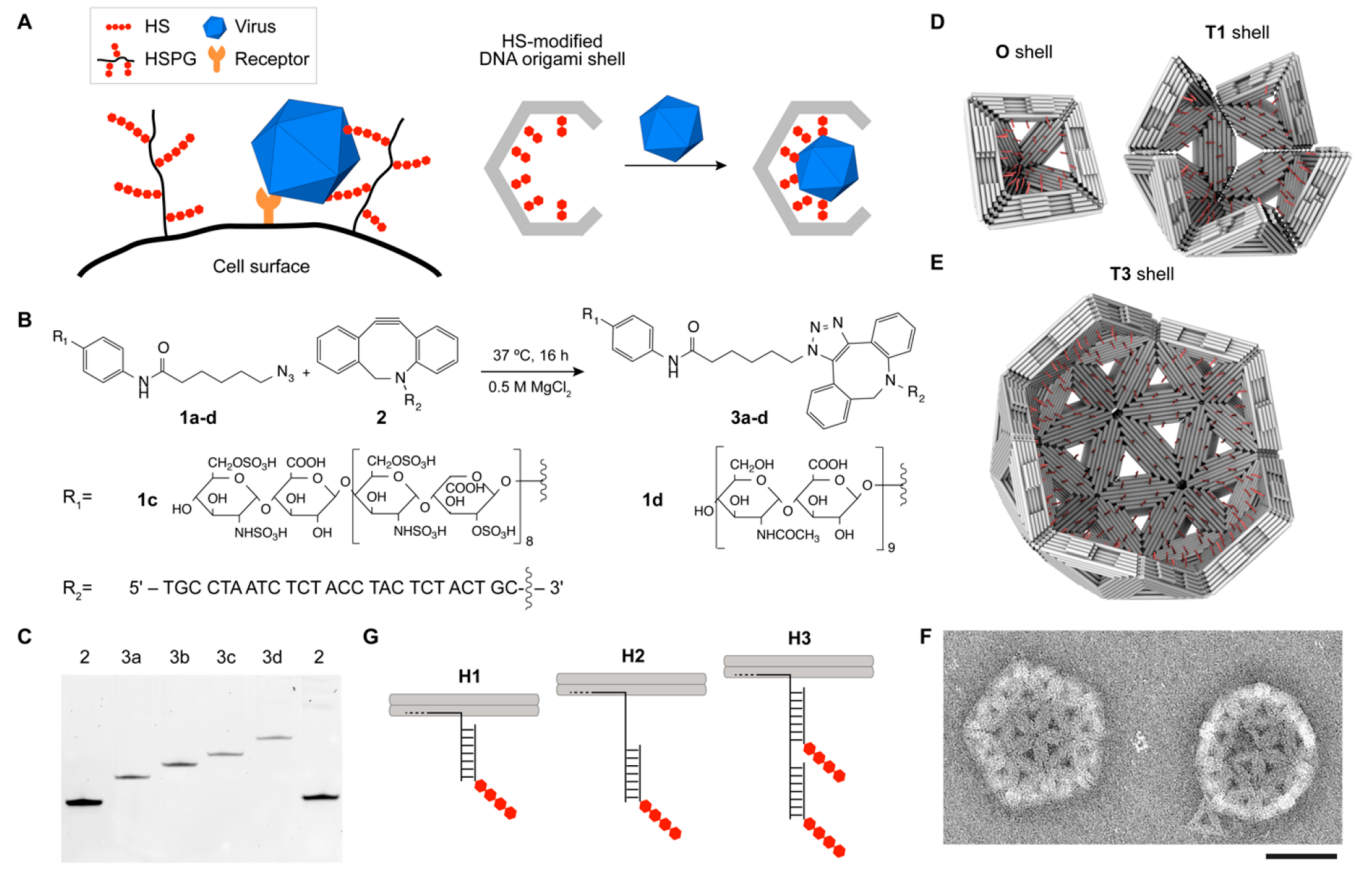
DNA origami shells and functionalization with HS derivatives. (A) A heparan sulfate proteoglycan (HSPG) interacts with a virus pathogen and mediates its cellular uptake (left). DNA origami shell schematic, with HS modifications in its interior, capable of binding and sequestering a viral particle (right). (B) SPAAC reaction in between azide-modified heparin and HS oligomers and a DBCO-modified DNA oligo. The DNA sequence is complementary to the handles of the DNA origami shells in (D, E). (C) PAGE characterization of the HS-modified DNA oligos. Products containing HS with sulfate and sulfonate groups (**3a** and **3c**) migrate at a faster rate through the gel than the analog negative controls (**3b** and **3d**) due to their increased anionic character. (D) Cylindrical models of **O** and **T1** shells made of 4 and 10 triangle subunits respectively, containing single stranded protruding oligos (termed handles, shown in red) decorating their interior. Each triangle subunit contains nine handle positions. (E) **T3** shell design consisting of 30 triangle subunits and featuring an inner cavity of 150 nm. Each triangle subunit also contains nine handle positions. (F) Negative stain TEM micrograph of **T3** shells. Scale bar is 100 nm. (G) Schematic representation of three different handle designs. **H1** contains one HS modification per handle placed as close to the origami surface as possible. **H2** also contains one HS modification per handle but has a polyT extension of 20 bases, allowing the handle to reach further than **H1**. **H3** mimics a branched polymer containing two HS modifications per handle unit, therefore doubling the local HS density.

The interactions of heparin and heparan sulfate derivatives with viruses have already been exploited for medical purposes, for example, in virus-sequestering coatings of condoms that are based on HS-decorated dendrimers.^7−9^ Other investigations have considered the surface functionalization of nanoparticles and polymers with HS derivatives to create virus-binding complexes with antiviral activity.^10−13^ Commonly, multivalency is required to increase the strength of binding between the HS nanoparticles and viruses to prevent the undesirable release of infectious viruses from the virus-sequestering coatings.^9^

Previously, we presented a concept for neutralizing viruses by encapsulation in macromolecular shells fabricated with DNA origami.^14^ The shells mechanically prevent interactions between trapped viruses and host cells. For the previous proof-of-concept experiments, we coated the inside of the shell with antibodies to sequester virus particles in the shells. One key advantage of the shells is that the virus-binding moieties used in their interior themselves do not need to have a neutralizing function since this task is performed by the shell material. Nonetheless, as discussed above, the use of antibodies in the virus trapping shells as in our previous work presents several challenges that may limit the usefulness of the virus-trapping concept. Here, we exploit the conserved background binding of HSPG to viruses to irreversibly trap viruses in HS-functionalized shells (Figure 1A, right panel).

## Results and Discussion

Using a strain-promoted azide–alkyne 1,3-dipolar cycloaddition reaction (SPAAC), we covalently attached a heparan sulfate (HS) derivative to a DNA oligonucleotide (Figure 1B, Figure S1). The HS-modified oligonucleotide can hybridize to single-stranded DNA extensions termed “handles” that are located on the inward-facing surfaces of the DNA origami shells. For the coupling, we used azide-group modified HS derivatives containing either 8 or 18 saccharide monomers (**1a** and **1c**, respectively), including monomers such as *N*-acetyl-glucosamine and glucuronic acid which characterize HS polymers. As controls, we used 8-mer and 18-mer polysaccharides lacking the sulfate and sulfonate groups (**1b** and **1d**, respectively). The DNA oligonucleotide to be clicked to the different HS polymers was modified with a dibenzocyclooctyne (DBCO) moiety (**2**), and the SPAAC reaction occurred rapidly upon mixture of both components. We analyzed the reaction products (**3a**–**d**) by polyacrylamide gel electrophoresis (PAGE), which revealed different electrophoretic mobilities for the different product versions consistent with expectation (Figure 1C). Higher molecular weight reaction products had slower mobility, and the sulfate-containing products migrated faster in the gel compared to the products lacking the sulfate groups, which we attribute to the additional negative charges. We then hybridized the HS-modified DNA oligonucleotides to sequence-complementary single-stranded DNA handles protruding from the target DNA origami shell’s interior surface.

To capture differently sized viruses, we fabricated three DNA origami shell variants and functionalized their interior with the same HS derivative. We used the previously described octahedral and *T* = 1 icosahedral half shell designs (**O** and **T1**, respectively) featuring 40 and 85 nm wide cavities, respectively (Figure 1D).^14^ We also developed a *T* = 3 icosahedral half shell design, termed **T3**, for the encapsulation of larger virus particles that do not fit into **O** or **T1** shells (Figure 1E). The **T3** design is a finite-size higher-order assembly consisting of a total of 30 triangular subunits, partitioned as five copies of six different full-size DNA origami triangle designs with specific edge docking rules (Figure S2). The resulting shell has a cavity diameter of approximately 150 nm. Negative stain transmission electron microscopy (TEM) images validate the successful assembly of **T3** shells (Figure 1F and Figure S3).

In experiments with adeno-associated virus serotype 2 (AAV2), we explored three different DNA handle designs: proximal (**H1**), distal (**H2**), and branched (**H3**) to determine the type and density of the HS modifications required for trapping viruses (Figure 1G). These experiments showed that **H1** and **H2** designs were not as efficient for virus trapping as the **H3** branched handle design. Blind TEM quantification of particles revealed ∼96% of shells to be occupied with AAV2 when **H3** was hybridized to the 18-mer HS derivative (**3c**), improving from the ∼30% and 84% of occupied shells achieved with **H1** and **H2**, respectively (Figure S4). We therefore used the branched handle design **H3**, and the HS 18-mer variant (**3c**) henceforth, unless otherwise specified. We confirmed that the interaction with AAV2 is due to the sulfate and sulfonate groups present in the HS structure, as the **3d** HS derivative used as a negative control did not demonstrate any binding (Figure S5). Importantly, all AAV2 particles were trapped with **O** shell excess (Figure S6).

**Figure 2:**
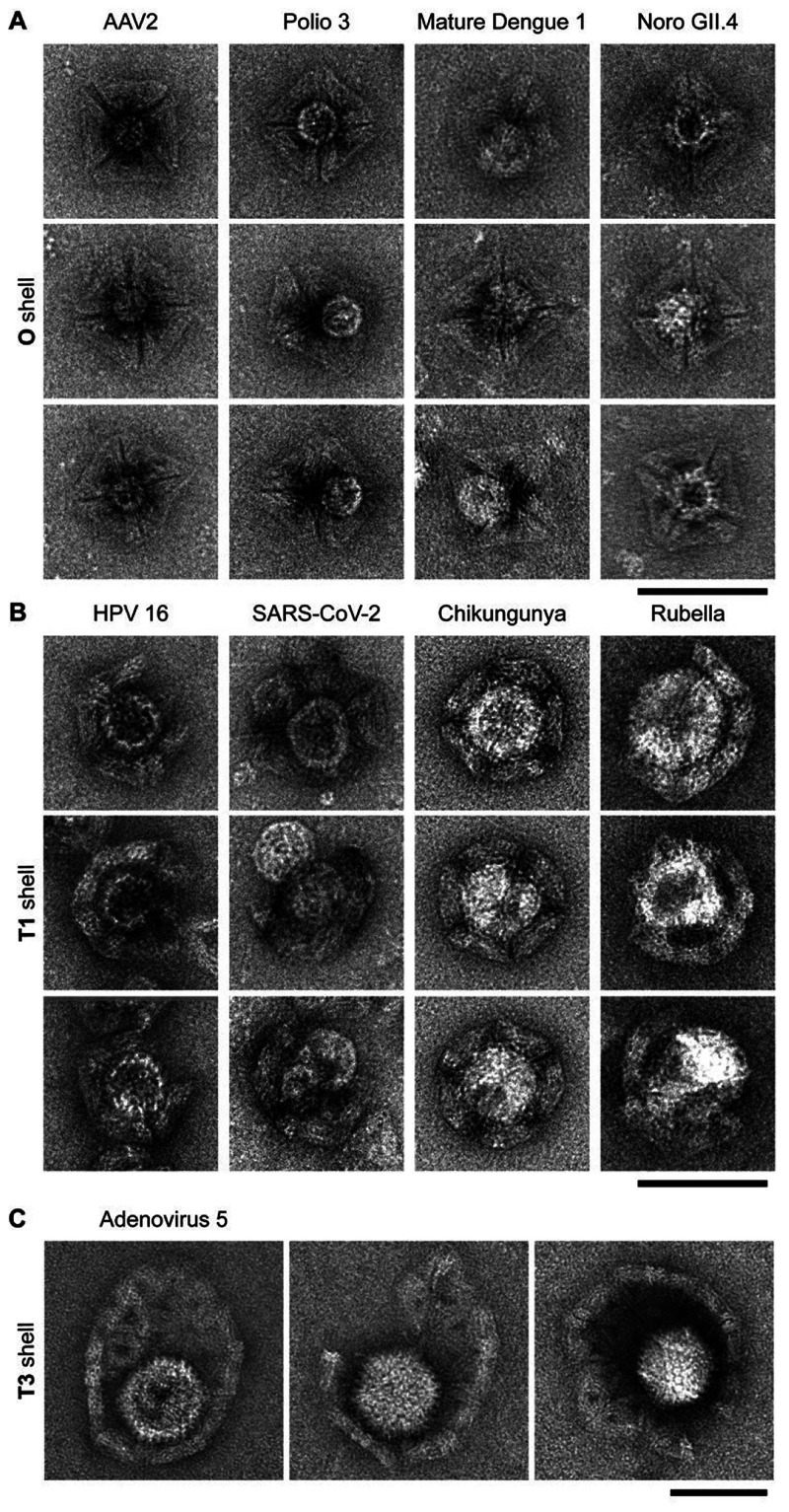
Viruses and VLPs trapped within HS-modified O, T1 and T3 shells. Negative stain TEM images of (A) AAV2, polio 3, mature dengue 1 and norovirus GII.4 successfully trapped in **O** shells. (B) HPV 16, SARS-CoV-2, chikungunya, and rubella engulfed by **T1** shells. (**C**) Adenovirus 5 captured with **T3** shells. Scale bars are 100 nm.

With the HS handle design thus established, we tested the HS-modified DNA origami shells for their ability to trap a variety of exemplary viruses and virus-like particles (VLPs).^15^ Our target virus library sampled enveloped and nonenveloped particles, particles from different viral families, and particles with dimensions ranging from 25 to 90 nm (Table 1, see also Figure S7 for TEM images). We used HS-modified **O** shells to sequester AAV2, poliovirus, mature dengue, and norovirus (Figure 2A). We used the larger HS-modified **T1** shells to trap human papilloma virus 16 (HPV 16), SARS-CoV-2, chikungunya, and rubella particles (Figure 2B), and the even larger HS-modified **T3** shell for enclosing adenovirus 5 (Figure 2C and Figure S8 for TEM tomography).

**Table 1:** Target Viruses and Virus-like Particles Tested in This Study

virus/VLP	family	enveloped	major surface components	genome type	measured diameter (nm)^c^	ref
AAV2^a^	Parvoviridae	no	3 capsid proteins	ssDNA	25	(29)
polio type 3	Picornaviridae	no	4 capsid proteins	ssRNA^b^	30	(30)
dengue type 1	Flaviviridae	yes	1 envelope protein	ssRNA^b^	30–40	(31)
noro GII.4	Caliciviridae	no	1 capsid protein	ssRNA^b^	30–45	(32)
HPV 16	Papillomaviridae	no	2 capsid proteins	dsDNA^b^	35–50	(33)
SARS-CoV-2	Coronaviridae	yes	1 envelope protein, 1 spike protein	ssRNA^b^	30–70	(34)
chikungunya	Togaviridae	yes	2 envelope proteins	ssRNA^b^	65–70	(35)
rubella	Matonaviridae	yes	2 envelope proteins	ssRNA^b^	65–80	(36)
adenovirus 5^a^	Adenoviridae	no	3 capsid proteins	dsDNA	90	(37)

a Infectious virus.

b Data referring to the infectious virus which is modeled by a VLP in this study.

c Measured using negative stain TEM or cryo-EM images.

**Figure 3:**
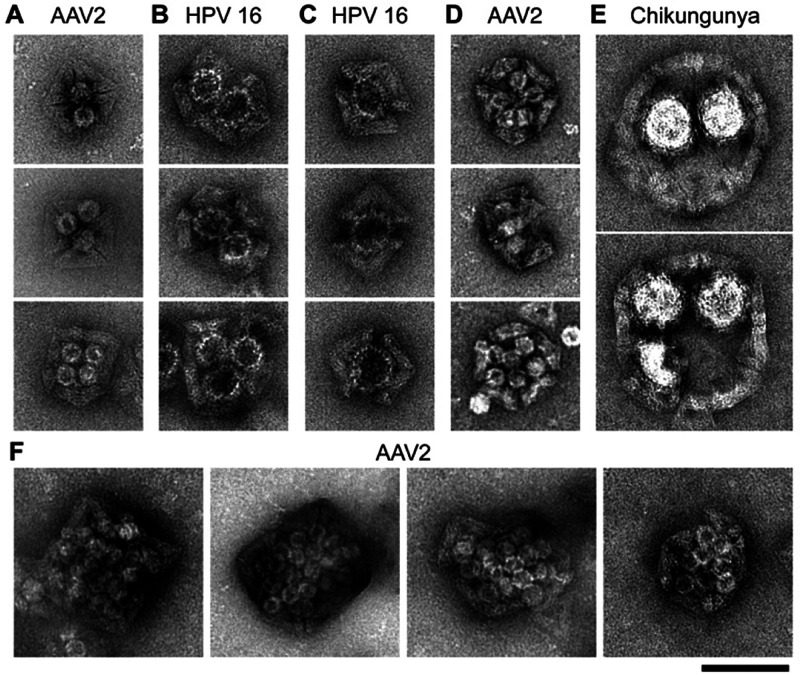
Multiple viruses and VLPs trapped in HS-modified **O**, **T1**, and **T3** shells. Negative stain TEM images of (A) up to four AAV2 in one **O** shell, (B) up to three HPV 16 in one **T1** shell, and (C) one HPV 16 coordinated by two **O** shells for complete occlusion of the virus particle. (D) Up to six AAV2 per **T1** shell. (E) Up to three chikungunya VLPs per **T3** shell. (F) Cooperative effect of multiple **O** shells capturing numerous AAV2 particles. Scale bar is 100 nm.

Interestingly, the multivalent interactions between the HS coating on the shell interior and the virus particles was sufficiently strong to support elastic deformations of the surrounding shell. For example, the **T3** shell material deformed from spherical to elliptical around adenovirus particles, presumably driven by maximization of the number of molecular interactions between the HS moieties on the shell interior surface and the viral surface, at the expense of elastically deforming the shell. The **O** shells deformed occasionally so that up to four AAV2 particles were accommodated in its cavity (Figure 3A), even though by design the **O** shell has room for only one AAV2 particle if it were completely rigid. The **T1** shell also flexed to fit up to three HPV 16 copies (Figure 3B). Depending on the relative stoichiometry between shells and virus particles, we also observed sandwich-like structures where two shells coordinated one virus particle (*e.g*., with HPV 16 and **O** shells, Figure 3C). If the shell diameter was substantially larger than the target virus dimensions (*i.e*., by a factor of 2 or more), multiple target particles could be sequestered. For example, we observed up to six AAV2 per **T1** shell (Figure 3D) and up to three chikungunya in **T3** shells (Figure 3E and Figure S9 for TEM tomography). Furthermore, multiple copies of HS-modified shells could also partition over and cover the surface of clusters of AAV2 particles (Figure 3F).

In the negative staining TEM images, we saw that the chikungunya VLP particles appeared to completely fill the **T1** cavity. Presumably due to the high surface contact area formed, chikungunya particles were trapped within **T1** shells using any of the different handle designs described in Figure 1G in high yields (**H1**, **3a**, 90% full shells). In fact, we could trap chikungunya even with the **3b** negative control, which are shells with a coating lacking the sulfate and sulfonate groups, albeit at a lower yield (**H1**, **3b**, 54% full shells, see also Figure S10). We attribute the finding that chikungunya particles can be trapped in **T1** even without specific shell functionalization due to cooperative amplification of weak electrostatic interactions between the negatively charged DNA shells and the chikungunya particles as they interact over extended surface areas.

Dengue virus, as well as some other viruses, present two distinct “mature” and “immature” conformations. In brief, dengue virus infectivity depends on the ability of envelope proteins to target cell heparan sulfates.^16^ The surfaces relevant for the interactions with HS are presumably hidden in the immature dengue virus conformations, which undergo conformational changes to become infectious, allowing them to move between vector and host, and/or infected and healthy cells.^17−20^ While the usage of VLPs is highly convenient for safety reasons, we do acknowledge some limitations. For instance, initially our dengue VLP samples contained a high percentage of immature particles which did not bind to our HS-functionalized shells. By contrast, when we induced enzymatic maturation of the dengue VLPs, to mimic the mature dengue as would occur *in vivo*, we did observe binding of the matured particles (Figure 2A dengue and Figure S11).

The unsuccessful trapping of immature dengue particles serves as a test for understanding the selectivity of our system. We performed further binding experiments with other viruses and similarly sized nanoparticles as additional negative controls. For instance, **O** shells showed no binding toward hepatitis B core particles as expected (Figure S12). Gold nanoparticles with diameters comparable to whole viruses (∼30 nm) conjugated to carboxylic acid functional groups (AuNP-COOH) showed no binding to our **O** shells. In a trapping competition study with both AuNP-COOH and AAV2, the AAV2 were selectively trapped within the **O** shell cavity, while the AuNP-COOH remained unbound (Figure S13). These experiments suggest that the electrostatic binding properties of the HS modifications can discriminate between functionalities and protein composition.

**Figure 4:**
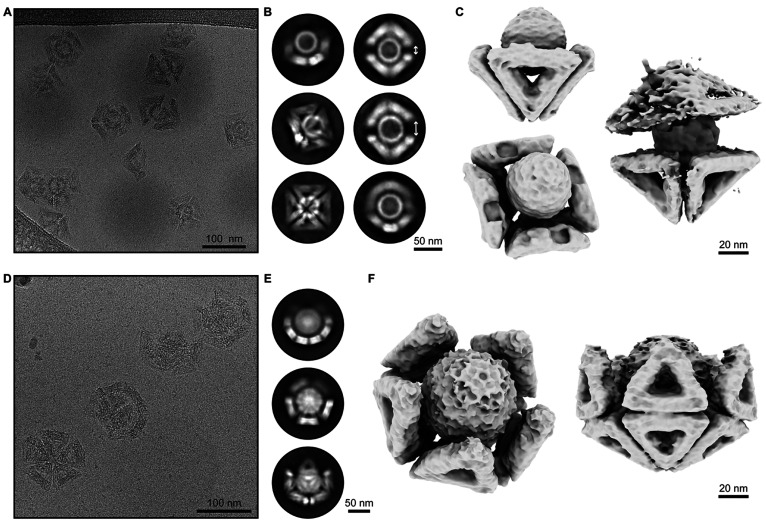
Cryo-EM analysis of virus-like particles trapped in DNA origami shells. (A) Cryo-EM micrograph of **O** shells binding to HPV 16 VLPs. (B) 2D class average images of one or two **O** shells binding to one HPV 16 particle, demonstrating different orientations of the complexes. The white arrows indicate the gap difference in between the two **O** shells, confirming the capture of differently sized VLP particles. (C) 3D reconstructions of HPV 16 bound to one and two **O** shells. (D) Cryo-EM micrograph of **T1** shells binding to chikungunya VLPs. (E) 2D class average images of **T1** shells binding to chikungunya particles showing different orientations of the complex. (F) Two different views of the 3D reconstruction of a **T1** shell engulfing a chikungunya virus particle.

We also performed cryogenic electron microscopy (cryo-EM) studies of HPV 16 and chikungunya VLPs trapped inside **O** and **T1** shells, respectively (Figure 4A,D). Two-dimensional (2D) class average images and 3D cryo-EM reconstructions confirmed that the VLPs were successfully trapped within the respective shell’s cavities (Figure 4B,E and Figures S14–S15). While one **O** shell is not sufficiently large to encapsulate an entire HPV 16 particle, two **O** copies can coordinate and completely cover an entire VLP (Figure 4C). 2D class averages of free HPV 16 showed a variation in particle sizes within the VLP sample (Figure S16). Consistently, we also found that the gap distances in between **O** shells (indicated by the white arrows in Figure 4B) varied depending on whether a smaller or larger HPV 16 particle was trapped. The cryo-EM map that we determined for the complex consisting of a chikungunya VLP in a HS-modified **T1** shell reveals the near-perfect fit between the two particles (Figure 4E,F). The cryo-EM maps provide compelling illustrations of the extent of relative dimensions of the artificial DNA origami shells relative to their viral targets and the extent of surface occlusion that can be achieved by sequestering viruses in shells. Quantification analyses of trapped viral population were performed from cryo-EM micrographs. For the HPV 16, 91.1% of the particles were found to be trapped within **O** shells, and 74.4% of chikungunya in **T1** shells (Figure S17).

To test the stability of virus trapping by the shells, we subjected exemplarily a sample consisting of AAV2 encapsulated in **O** shells to a dilution series. The fraction of occupied shells remained comparable within margin of error prior to and after a 100-fold dilution and incubation for 14 days in the diluted sample relative to the nondiluted sample (Figure S18). This observation suggests that the spontaneous dissociation rate for the complexes formed between AAV2 particles and the surrounding HS-modified shell is on the scale of weeks under the conditions tested. The high stability is presumably avidity-driven and can be understood by considering that a spontaneous dissociation of AAV2 from a surrounding shell requires simultaneously breaking of dozens of bonds formed between HS chains on the engulfing shell and the virus surface. The likelihood for such event to happen decreases exponentially with the number of HS bonds formed. Furthermore, we also performed virus trapping experiments in the presence of serum to demonstrate the capabilities of the system to bind to virus particles in biologically relevant conditions. Exemplarily, AAV2 were successfully trapped with **O** shells in the presence of cell culture media containing 10% FBS (Figure S19).

To examine the capacity of our shells to prevent trapped viruses from undergoing interactions with surfaces, we performed *in vitro* virus-blocking ELISA assays with AAV2 (Figure S20) as previously established.^14^ We quantified the extent of AAV2 binding to antibodies immobilized on a solid surface through the binding of orthogonal AAV2-specific reporter antibodies coupled to horseradish peroxidase (HRP). Residual AAV2 particles that are bound to the surface are detected by HRP-catalyzed production of a colorimetric signal. The AAV2 trapping abilities of HS-modified **T1** shells were tested and compared to an unfunctionalized **T1** shell control. The HS-modified **T1** shells demonstrated a significantly lower concentration of AAV2 particles available for binding when compared to the unmodified **T1** shell control. In addition, AAV2 particles directly incubated with an equivalent concentration of HS **1c** (without any shells present) were also blocked from binding to the surface but with reduced blocking capacity compared to when using HS-modified shells. This finding indicates that the HS by themselves do not fully passivate the virus surface and that the DNA origami material shields the virus from its exterior by steric occlusion and block potential binding interactions.

## Conclusions

We presented a viral trapping system that targets features of viruses that are conserved across many families through the usage of HS derivatives. Overall, we achieved encapsulation of nine different virus and VLP test samples, each representing a different viral family, and different sizes and surface complexities. Our modular shell system creates a locally curved environment within the cavity that enables multivalent binding between HS and viral surfaces and that can be optimized according to size and ligand density/type. Our shells can flex and adapt to a certain degree to the shape of trapped virus particles, suggesting that the shell system can also adapt to pleomorphic virus particles. We envision that our HS-modified DNA origami shells can act as a cellular surface decoy, sequestering the viruses and preventing interactions with cell surfaces, and thus reduce the effective viral load in acute infections. Testing the therapeutic potential of this system to reduce viral load *in vivo* remains an important task for the future. Beyond potential virus neutralization, our system may also serve as a sink for trapping associated viral proteins (Figure S21) and other side products such as subviral particles that could potentially overwhelm the immune system.^9,21^

## Methods

Staple strands for origami folding reactions were purchased from Integrated DNA Technologies (IDT) and used with standard desalting purification unless stated otherwise. DBCO-modified handle strands were purchased from Biomers at HPLC grade. Azide-modified heparan sulfate derivatives were purchased from Glycan Therapeutics. VLPs were purchased from The Native Antigen Company, Creative Biostructure, and Creative Biolabs (catalog references can be found in Table S1).

### Folding of DNA Origami Triangular Subunits

DNA origami structures were folded in one-pot reactions containing 50 nM of single-stranded scaffold DNA (M13, 8064 bases) and 250 nM of each staple strand in a standardized “folding buffer” (FoBx) containing *x* = 20 mM MgCl_2_, 5 mM Tris base, 1 mM EDTA, and 5 mM NaCl at pH 8.00. Scaffold M13 was produced as previously described (Supplementary Note 1 for sequence).^22^ All folding reactions were subjected to optimized thermal annealing ramps (Table S2) in a Tetrad (Bio-Rad) thermal cycling device.

### Purification of Triangle Subunits and Shells Self-Assembly

All origami structures were purified using agarose gel extraction (1.5% agarose containing 0.5× TBE and 5.5 mM MgCl_2_) and centrifuged for 30 min at maximum speed for residual agarose pelleting. If a concentration step was needed, ultrafiltration (Amicon Ultra 500 μL with 100 kDa molecular weight cutoff) was performed prior to shell assembly. For shell assembly, the purified triangles were mixed in a 1:1 ratio. Typical triangle subunit concentrations ranged from 5 to 400 nM, while assembly times depended on the shell type. Table S3 summarizes and offers a comparison on the optimized salt concentrations, temperature, and self-assembly times required for all shells used in this study. The assembled shells were UV cross-linked for 1 h at 310 nm using Asahi Spectra Xenon Light source 300 W MAX-303.^23^

### Heparan Sulfate Attachment to DNA

Excess of azide-modified heparan sulfate derivatives (**1a**–**d**) were mixed in a 4:1 ratio with DBCO-modified DNA to form the respective products (**3a**–**d**). MgCl_2_ was added to a 0.5 M concentration, and the mixture was left overnight at 37 °C to achieve >90% conversion. The products were run in a preparative 10% PAGE gel for 2 h at 35 W. Subsequently, the product bands were cut away and were crushed. 1× TEN buffer (10 mM Tris-HCl, 1 mM EDTA, 100 mM NaCl, pH 8.00) was added to dissolve and recover the modified oligonucleotides, and EtOH precipitation was used for concentration and buffer exchange. The pure products were redissolved and kept in double distilled H_2_O at either 4 °C or −20 °C. Hybridization of the DNA-HS handles to the complementary handles protruding from the shell’s interior was performed at r.t. overnight in FoB40. Buffer exchange to 1× PBS containing 10 mM MgCl_2_ was then performed prior to VLP encapsulation experiments using ultrafiltration (Amicon Ultra 500 μL with 100 kDa molecular weight cutoff) or dialysis (D-Tube Dialyzer Mini, MWCO 12–14 kDa, 2 × 500 mL exchanges over 8 h, r.t).

### Viruses and VLPs Encapsulation

Preassembled and UV-welded shells in 1× PBS containing 10 mM MgCl_2_ were mixed with a VLP sample in the appropriate ratio to achieve either shell or VLP excess. The samples were incubated at r.t. for 2 h. Trapping experiments with cell culture media (DMEM, high glucose, GlutaMAX Supplement, pyruvate Catalog number: 31966047 + 10% heat inactivated fetal bovine serum (HI-FBS)) were incubated at 37 **°**C for 2 h. Usual amounts of sample for TEM analysis ranged from 5 to 10 μL total solution at ∼10 nM triangle origami concentration. Negative stain TEM grids were prepared immediately after the 2 h incubation.

### *In Vitro* Virus-Blocking ELISA Assay

A fixed amount of AAV2 particles was incubated with either 1× PBS containing 10 mM MgCl_2_ or **T1** half shells without any HS modification (positive controls), a dilution series of the **T1** shells with interior HS modifications, or the equivalent concentration of unmodified HS. 1× PBS containing 10 mM MgCl_2_ was used as a negative/blank control. Samples were incubated for 2 h prior to being diluted in 1× PBS containing 10 mM MgCl_2_ and 0.05% Tween-20) and transferred to the ELISA plate (PRAAV2XP, Progen). From here, the capture ELISA kit was used as per manufacturer’s protocol. End-point absorbance measurements (OD) were made at both 450 and 650 nm (CLARIOstar, BMG LABTECH). Final OD measurements were calculated by subtraction of individual reference wavelength (650 nm) values and blank controls. Samples were repeated in triplicate.

### Maturation of Dengue VLPs

Dengue VLP maturation was adapted by methods described by Yu et al.^17,18^ Briefly, dengue VLP sample (10 μL, 0.39 mg/mL, The Native Antigen Company, cat. no. DENV1-VLP) was added to MES buffer (10 μL, 50 mM, pH 6.00) and gently mixed. Next, CaCl_2 (aq)_ (0.75 μL 0.1 M) and furin (3.9 μL, 2000 U/mL, New England Biolabs, Cat. No. P8077) were added and mixed, and the sample was incubated at 30 °C for 16 h. After incubation, Tris buffer (25 μL 100 mM Tris-HCl, 120 mM NaCl, pH 8.00) was added to the sample, and the sample was immediately dialyzed against 1× PBS (D-Tube Dialyzer Mini, MWCO 12–14 kDa, 2 × 50 mL exchanges over 24 h, 4 °C). Matured dengue VLP sample was used immediately and stored at 4 °C.

### Negative Staining TEM

Samples were incubated on glow discharged (45 s, 35 mA) formvar carbon-coated Cu400 TEM grids (Electron Microscopy Sciences) for 90–120 s depending on origami and MgCl_2_ concentrations. Next, the grids were stained for 30 s with 2% aqueous uranyl formate containing 25 mM NaOH. Imaging was performed with magnifications in between 10000× and 42000× in a SerialEM at a FEI Tecnai T12 microscope operated at 120 kV with a Tietz TEMCAM-F416 camera. TEM micrographs were high-pass filtered to remove long-range staining gradients, and the contrast was autoleveled using Adobe Photoshop CS5. To obtain TEM statistics in an unbiased fashion, automatic grid montages were acquired. For detailed information on selected particles, negative stain EM tomography was used as a visualization technique. The tilt series were performed from −50° to +50°, and micrographs were acquired in 2° increments.

Tilt series were processed with Etomo (IMOD) to acquire tomograms.^24^ The micrographs were aligned to each other by calculating a cross correlation of the consecutive tilt series images. The tomogram was then generated using a filtered back-projection. The Gaussian-Filter used a cutoff between 0.25 and 0.5, and a falloff of 0.035.

### Cryo-EM

DNA origami shells were prepared and functionalized, and viruses were trapped as described above. Samples (**O** + HPV: 70 nM triangles; **T1** + chikungunya: 200 nM triangles) were incubated 60 s on glow-discharged lacey carbon 400-mesh copper grids with an ultrathin carbon film. Free HPV 16 VLPs were applied to C-flat 1.2/1.3 grids (Protochip). Subsequently, the grids were plunge frozen in liquid ethane with a FEI Vitrobot Mark V (blot time: 2.5 s, blot force: −1, drain time: 0 s, 22 °C, 100% humidity, 3 μL sample). Cryo-EM imaging was performed with a spherical-aberration (Cs)-corrected Titan Krios G2 electron microscope (Thermo Fisher) operated with 300 kV and equipped with a Falcon III 4k direct electron detector (Thermo Fisher). Automated image acquisition was performed in EPU 2.6 (dose: 42–45 e^–^/Å^2^, exposure time: 3–5 s, 12 fractions, pixel size: 0.23 nm (**O** + HPV) and 0.29 nm (**T1** + chikungunya), defocus: −1.5 to −2 μm). Micrographs were processed in RELION-3^25^ using MotionCor2^26^ and CTFFIND4.1.^27^ Particles were automatically picked with crYOLO 1.7.6.^28^ Extracted particle images were classified and selected by visual inspection through multiple rounds of 2D and 3D classifications. Initial models were generated in silico in RELION-3. 3D reconstructions and multibody refinement yielded electron density maps with resolutions of 26 Å for **O** shells trapping HPV (EMD-13884, 1x **O** + HPV: 7834 particles, 2x **O** + HPV: 4634 particles) and 36 Å for **T1** shells trapping chikungunya (EMD-13883, 1259 particles, C5 symmetry).

## Supporting Information Available

Methods and additional data, including further characterization of HS derivatives, handle positions, viruses/VLPs, and binding conditions. All staple sequences for each triangular subunit/shell, in addition to assembly and folding conditions (PDF)
