# Supplementary material for: Broad-Spectrum Virus Trapping with Heparan Sulfate-Modified DNA Origami Shells
Source: ACS Nano. 2022 Nov 2;16(12):20002–9. doi: 10.1021/acsnano.1c11328 (PMC9798855; doi:10.1021/acsnano.1c11328)
Supplement: Supplementary file 1 — nn1c11328_si_001.pdf [file nn1c11328_si_001.pdf]

Supporting Information

Broad-spectrum virus trapping with heparan sulfate-modified DNA origami shells

Alba Monferrer,[1,2,†] Jessica A. Kretzmann,[1,2,†] Christian Sigl,[1,2] Pia Sapelza,[1,2] Anna Liedl,[1,2] Barbara Wittmann,[1,2] and Hendrik Dietz[1,2,*]

Correspondence to: dietz@tum.de

[1] Laboratory for Biomolecular Nanotechnology. Department of Physics, Technical University of Munich, Am Coulombwall 4a, 85748 Garching, Germany.

[2] Munich Institute of Biomedical Engineering, Technical University of Munich, Boltzmannstraße 11, 85748 Garching, Germany.

TABLE OF CONTENTS

Figure S1. SPAAC reaction for 8-mer HS derivatives (1a and 1b). 3
Figure S2. T3 shell design. 4
Figure S3. TEM field of view of T3 shells. 5
Figure S4. TEM quantification of O shells for AAV2 trapping with the different handle designs. 6
Figure S5. TEM of negative control for AAV2 trapping in O shells. 7
Figure S6. TEM of AAV2 trapping with O shell excess. 8
Figure S7. TEM of free viruses and VLPs. 9
Figure S8. TEM tomography of adenovirus 5 in a T3 shell. 10
Figure S9. TEM tomography of chikungunya VLPs in a T3 shell. 11
Figure S10. TEM quantification of T1 shells trapping chikungunya VLPs with the 3a and 3b HS derivatives. 12
Figure S11. TEM of immature and mature dengue 1 VLPs trapping with O shells. 13
Figure S12. TEM of O shell with Hepatitis B core particles. 14
Figure S13. TEM of O shells with AuNP-COOH. 15
Figure S14. Cryo-EM imaging of O shells trapping HPV 16 particles (EMD-13884). 16
Figure S15. Cryo-EM imaging of T1 shells trapping chikungunya VLPs (EMD-13883). 17
Figure S16. 2D class averages of free HPV 16 VLPs extracted from cryo-EM images. 18
Figure S17. Quantification of trapped viral population in DNA origami shells. 19
Figure S18. Stability of virus trapping by the shells. 20
Figure S19. Virus trapping in biologically relevant media. 21
Figure S20. *In vitro* AAV2-blocking via an ELISA assay. 22
Figure S21. TEM of rubella protein debris trapping in T1 shells. 23

Table S1. VLP providers and catalog references. 24
Table S2. Temperature ramps and scaffold used for each DNA origami triangle subunit. 25
Table S3. Shells assembling conditions. 26
Table S4. Staple sequences used for the octahedron triangle (T_octa) folding. 27
Table S5. Staple sequences used for the T1 pentamer triangle folding. 32
Table S6. Staple sequences used for the T1 ring triangle folding. 37
Table S7. Staple sequences used for the T3 triangle 1 folding. 43
Table S8. Staple sequences used for the T3 triangle 2 folding. 48
Table S9. Staple sequences used for the T3 triangle 3 folding. 53
Table S10. Staple sequences used for the T3 triangle 4 folding. 58
Table S11. Staple sequences used for the T3 triangle 5 folding. 63
Table S12. Staple sequences used for the T3 triangle 6 folding. 68

Supplementary Note 1 73
References 77

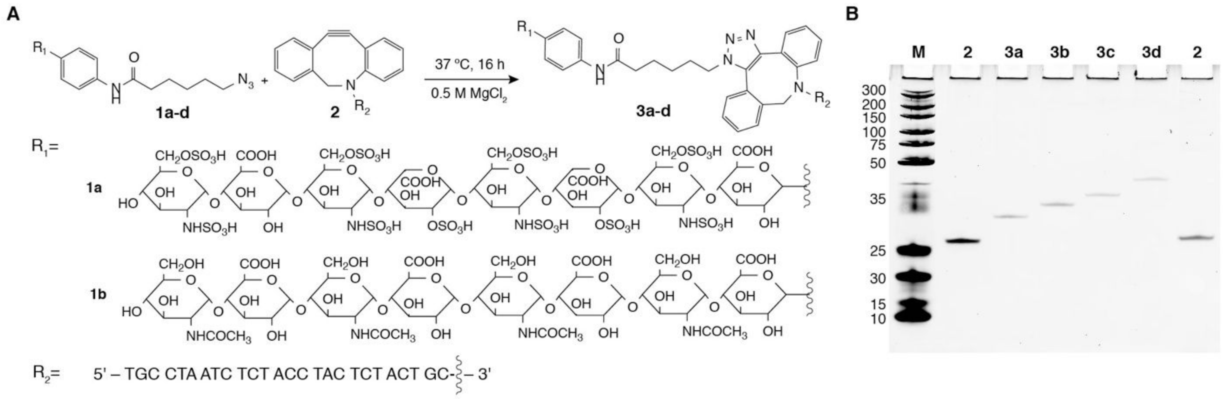

Figure S1. SPAAC reaction for 8-mer HS derivatives (1a and 1b).
(**A**) Click chemistry reaction in between azide-modified HS polymers and a DBCO-modified DNA oligo. The DNA sequence is complementary to the handles on the DNA origami shells. (**B**) PAGE characterization of all the HS-modified DNA oligos. M stands for marker.

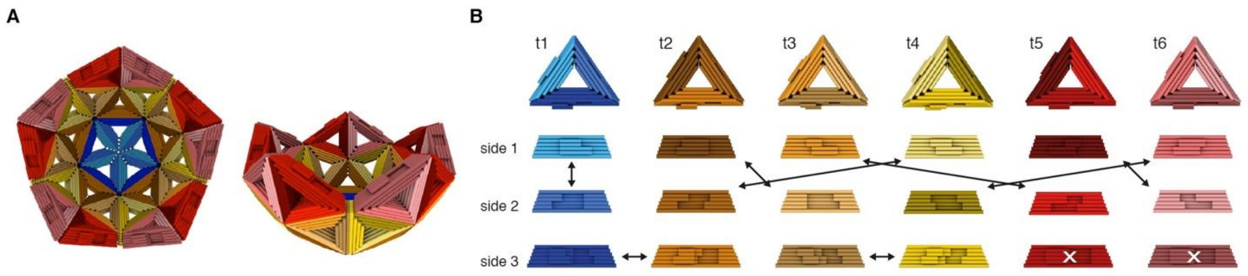

Figure S2. T3 shell design.
(**A**), Top and front view of a **T3** cylindrical model. (**B**) Cylindrical models of the triangles t1-t6 involved in the **T3** shell assembly. Arrows indicate complementary side interactions.

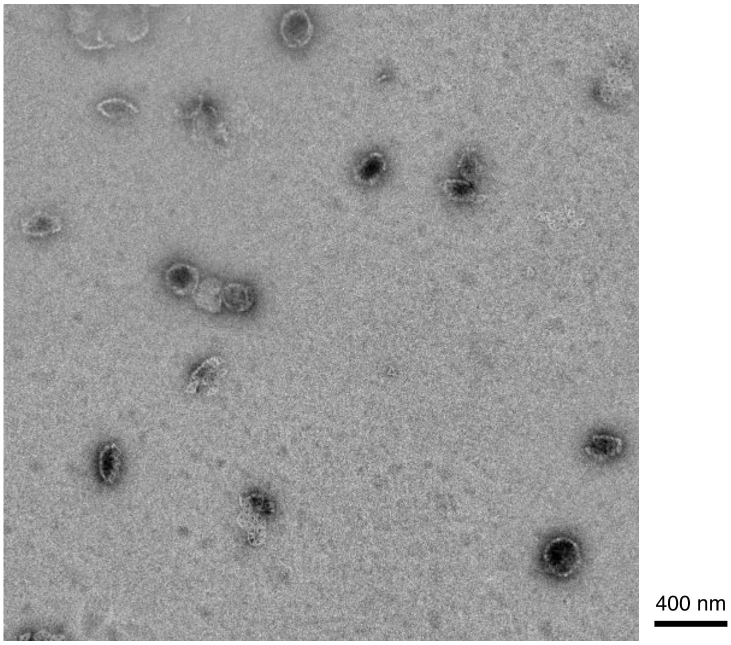

Figure S3. TEM field of view of T3 shells.
The **T3** DNA origami shells presented an inner diameter of ~ 150 nm. Due to their flexibility, some appear deformed on the grid. Scale bar is 400 nm.

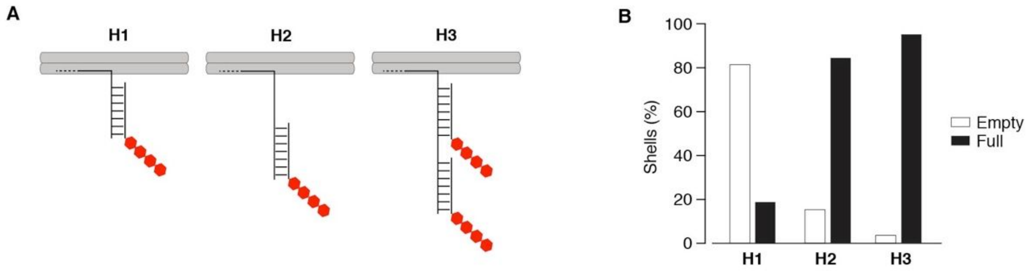

Figure S4. TEM quantification of O shells for AAV2 trapping with the different handle designs. (**A**) **H1**, **H2** and **H3** handle designs. (**B**) Blind TEM quantification of full vs. empty shells of each handle design when functionalized with **3c** HS derivative and AAV2 excess. **H1** presented ~ 30 % of full shells, **H2** ~ 84 %, and **H3** ~ 96 %.

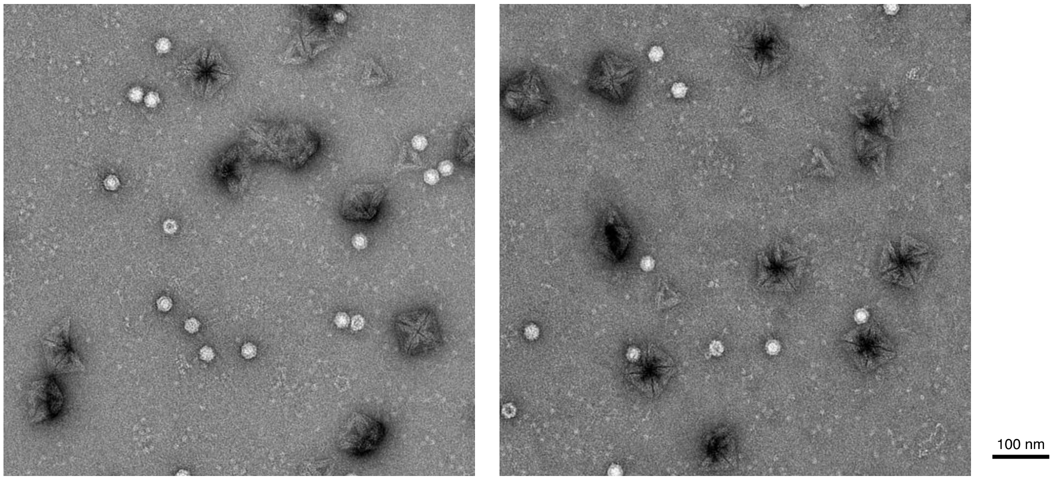

Figure S5. TEM of negative control for AAV2 trapping in O shells.
Fields of view demonstrating no binding of AAV2 viral particles when the **3d** negative control HS modification was hybridized to **H3** handle design. Scale bar is 100 nm.

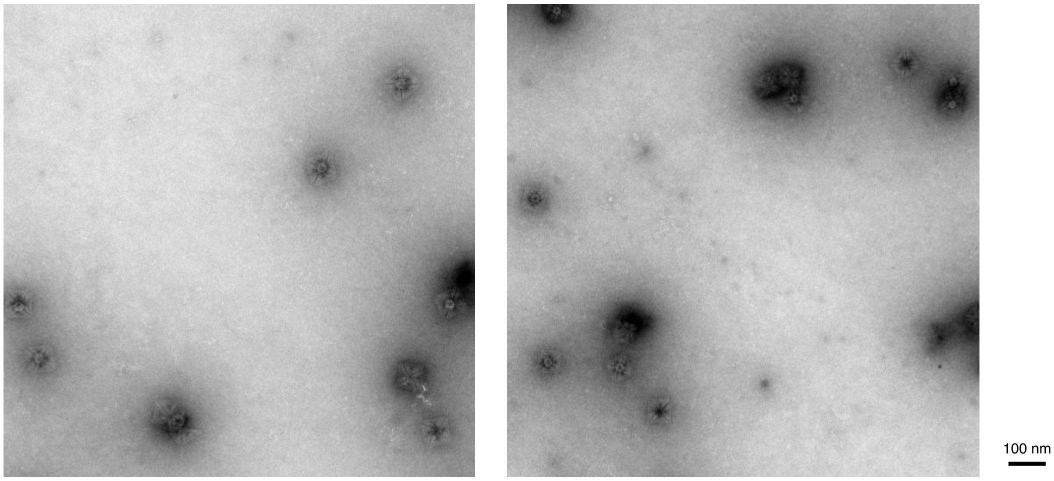

Figure S6. TEM of AAV2 trapping with O shell excess.
Fields of view demonstrating encapsulation of all AAV2 particles when the origami shell in excess. Scale bar is 100 nm.

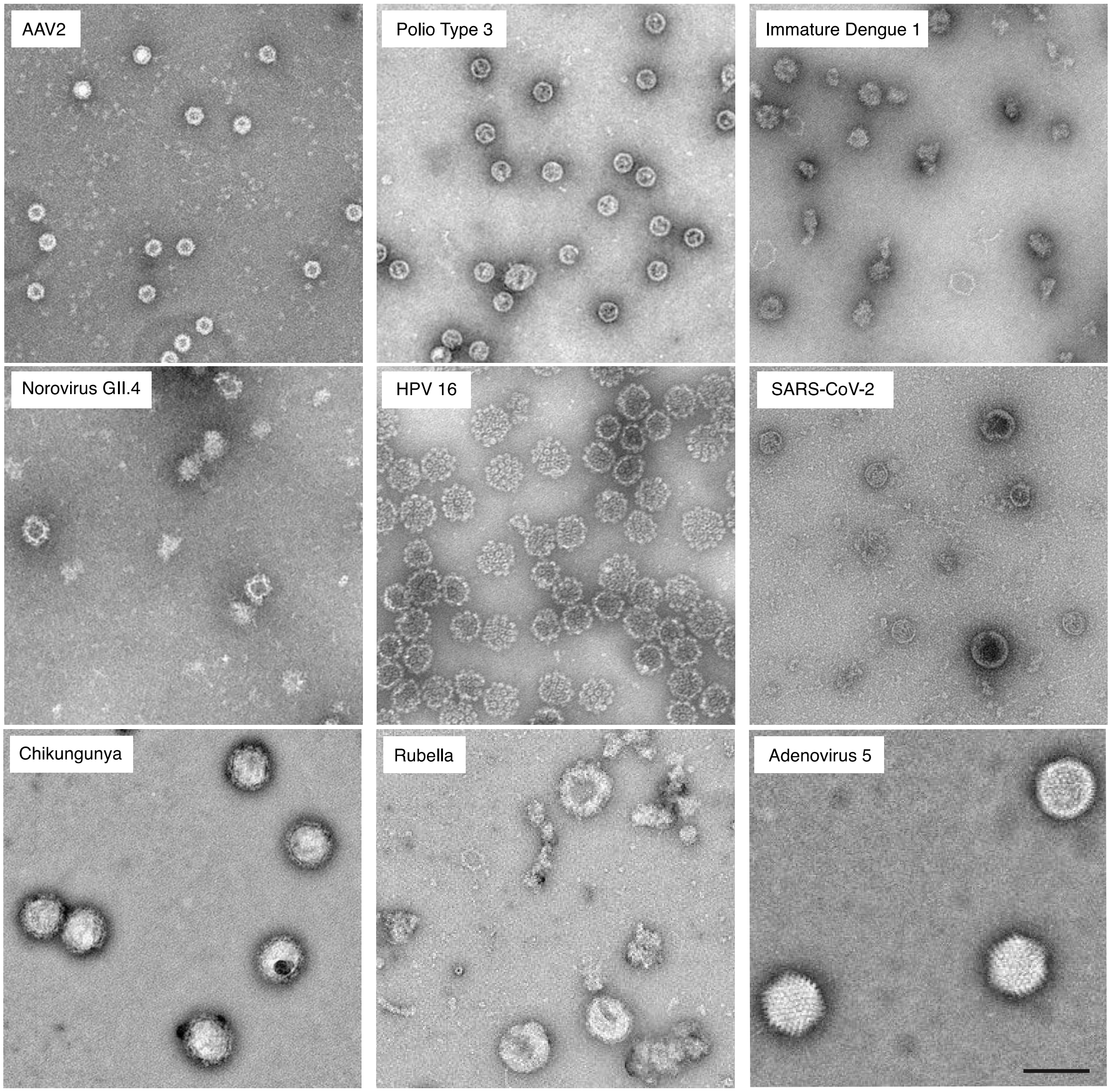

Figure S7. TEM of free viruses and VLPs.
Representative negative stain TEM micrographs of all virus and VLP samples used. AAV2, poliovirus, HPV 16, chikungunya and adenovirus 5 are the purest samples of our library, while dengue, norovirus, SARS-CoV-2 and rubella visibly contained a higher amount of protein debris, and presented a variable range of particle sizes. Scale bar is 100 nm.

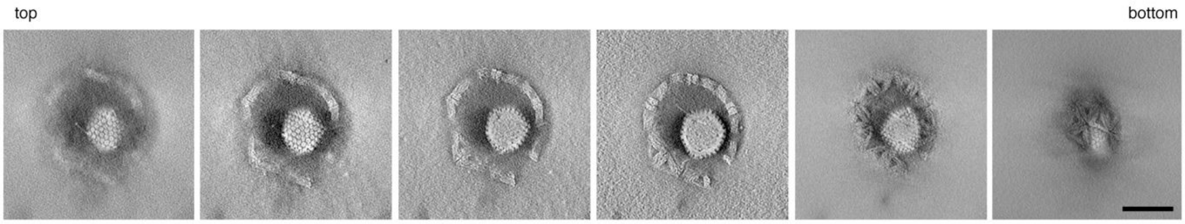

Figure S8. TEM tomography of adenovirus 5 in a T3 shell.
The slices of the tomogram calculated from an EM tilt series proved the full encapsulation of an adenovirus in the selected shell particle. Scale bar is 100 nm.

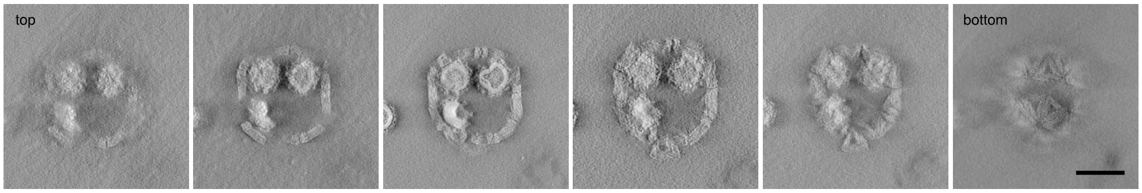

Figure S9. TEM tomography of chikungunya VLPs in a T3 shell.
The slices of the tomogram calculated from an EM tilt series proved the full encapsulation of three chikungunya VLPs in the selected shell particle. The last image slice showed a disruption of the triangles' connectivity. It was not clear if this discontinuity was due to the shell's rearrangement to encapsulate multiple VLPs or a consequence deforming on the grid during the sample preparation. Scale bar is 100 nm.

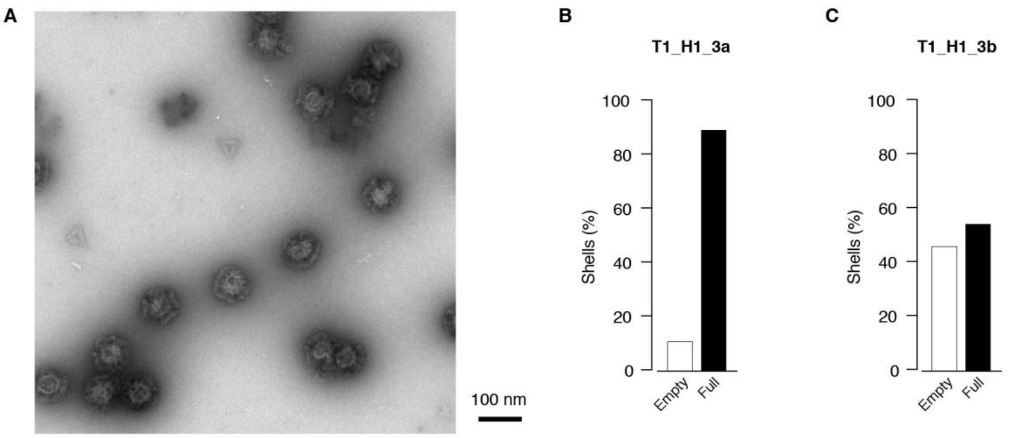

Figure S10. TEM quantification of T1 shells trapping chikungunya VLPs with the 3a and 3b HS derivatives.
(**A**) Negative stain TEM micrograph of **T1** shells functionalized with the **3b** negative control HS derivative, encapsulating chikungunya VLPs. Due to the size and shape complementarity, weak electrostatic interactions in between the DNA and the chikungunya VLP were sufficient to keep the virus particles encapsulated when the negative control handles were used. Scale bar is 100 nm. (**B**) TEM quantification of full vs. empty **T1** shells functionalized with the **3a** HS derivative on a **H1** handle design where ~ 90 % of shells were full. (**C**) TEM quantification of full vs. empty **T1** shells functionalized with the **3b** negative control HS derivative on a **H1** handle design where ~ 54 % of shells were full.

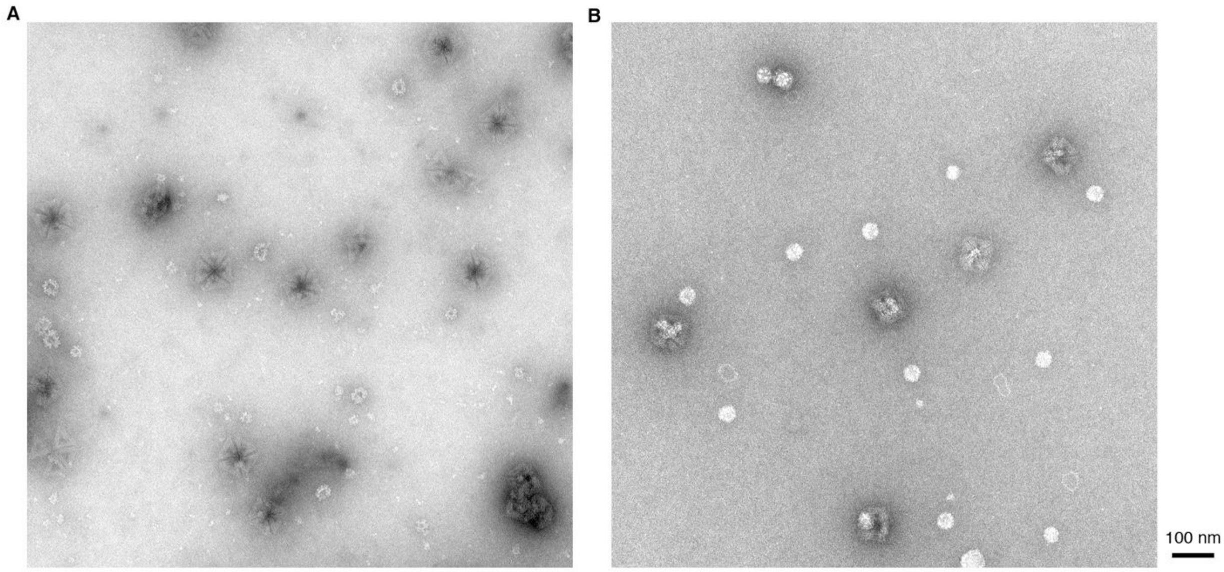

Figure S11. TEM of immature and mature dengue 1 VLPs trapping with O shells.
(**A**) The immature configuration of the dengue VLPs demonstrated no binding to the HS-modified origami shells. (**B**) Mature dengue VLPs were encapsulated by the **O** shells. VLPs were used in excess in both (A) and (B). Scale bar is 100 nm.

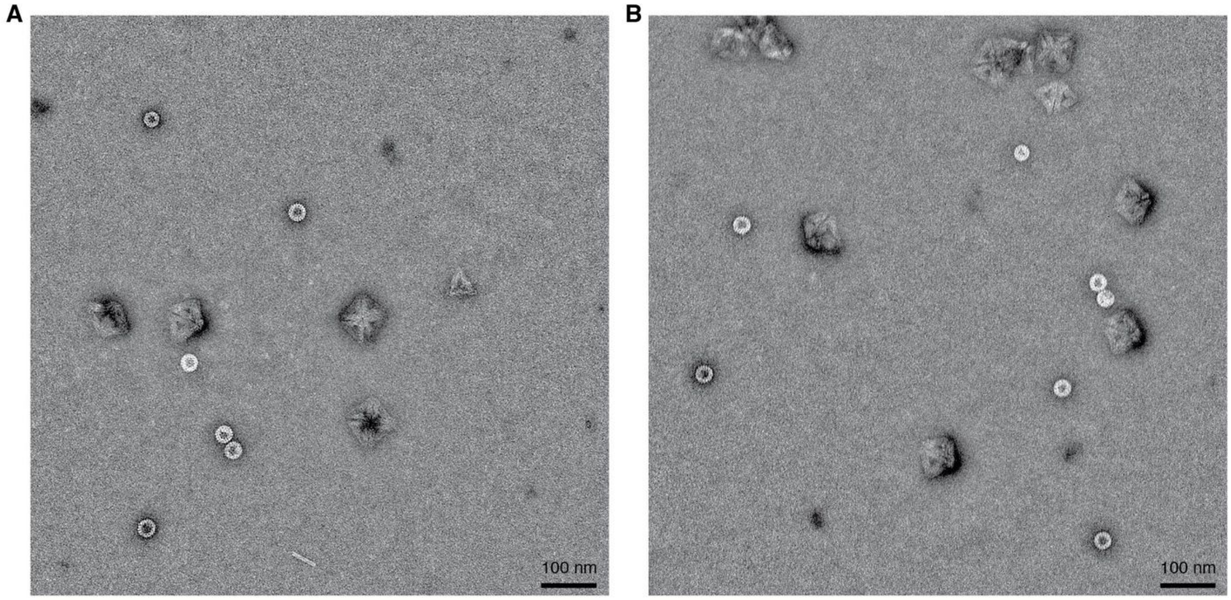

Figure S12. TEM of O shell with Hepatitis B core particles.
Fields of view demonstrating no encapsulation of Hepatitis B core particles with **O** shells functionalized with compound **3c**. These results serve as a negative control and suggest that HS can discriminate between protein composition. Scale bars are 100 nm.

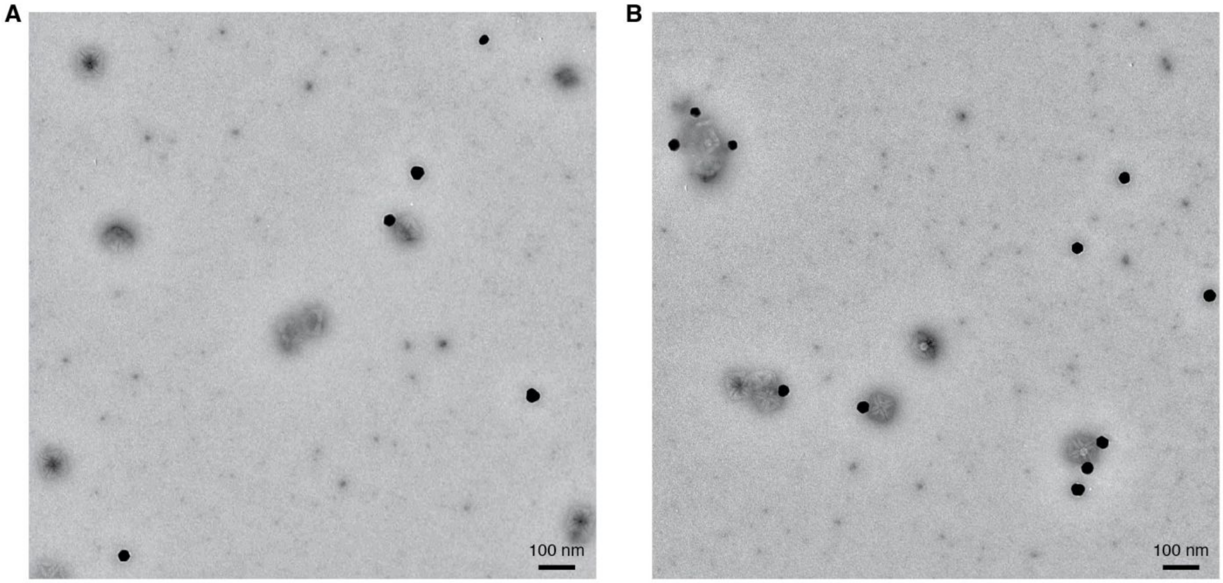

Figure S13. TEM of O shells with AuNP-COOH.
(**A**) Field of view demonstrating no encapsulation of AuNP-COOH with **O** shells functionalized with compound **3c**. (**B**) Binding competition study with AAV2 and AuNP-COOH, showing specific binding of AAV2 over AuNP-COOH. These results serve as an additional negative control and suggest that HS can discriminate between nanoparticle's surface functionalization. Scale bars are 100 nm.

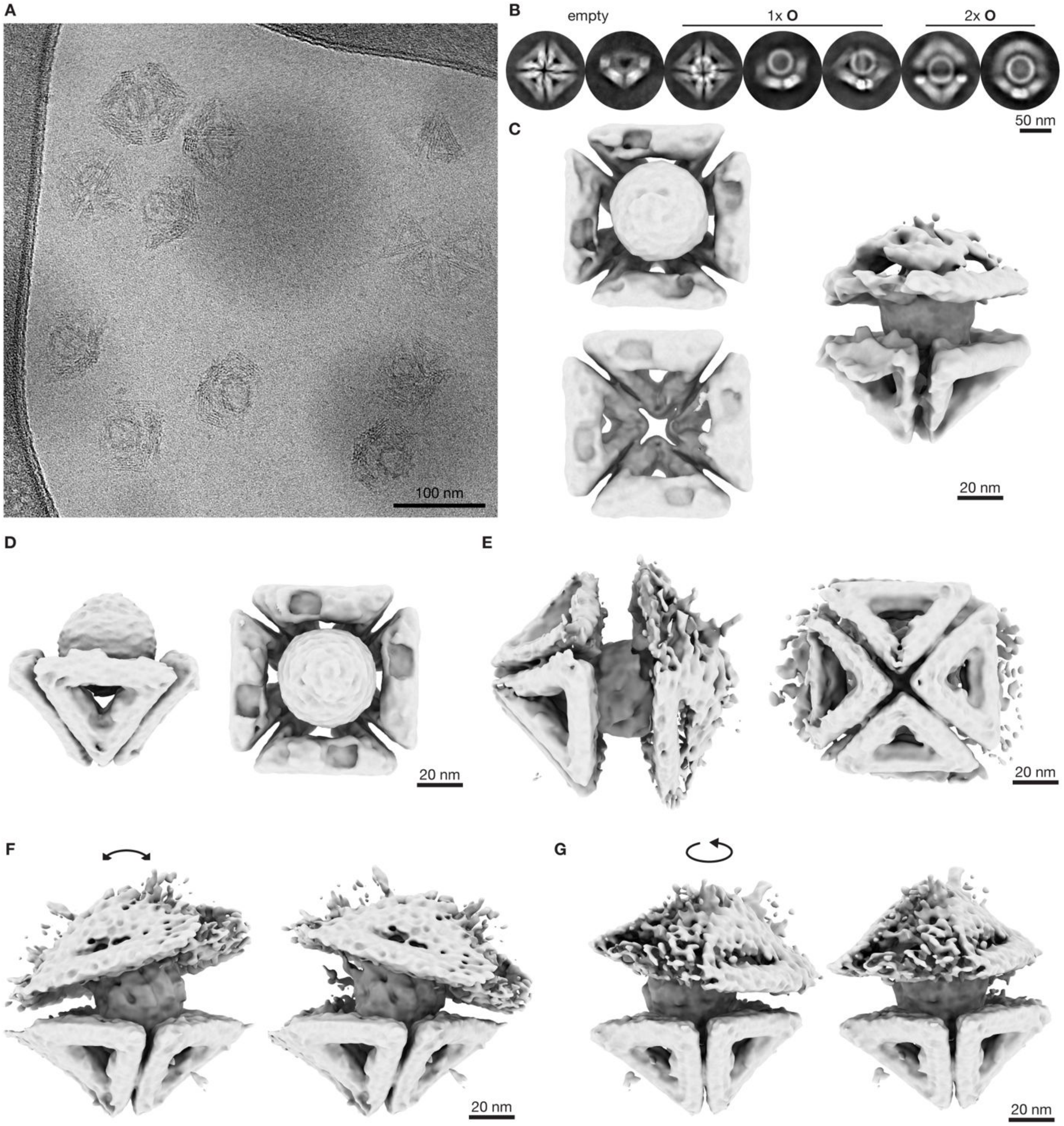

Figure S14. Cryo-EM imaging of O shells trapping HPV 16 particles (EMD-13884).
(**A**) Exemplary micrograph of **O** shells trapping HPV 16 vitrified on lacey carbon grids with ultrathin carbon support. (**B**) 2D class averages of empty shells (left), HPV 16 trapped by one **O** shell (middle), and two **O** shells trapping an HPV 16 (right). (**C**) 3D classes of selected particles showing similar particles as in B. (**D**) 3D reconstruction of HPV 16 particles trapped in one **O** shell. (**E**) Multibody refinement of HPV 16 particles encapsulated by two **O** shells. (**F**) and (**G**), Multi-component analysis of two **O** shells trapping an HPV 16.

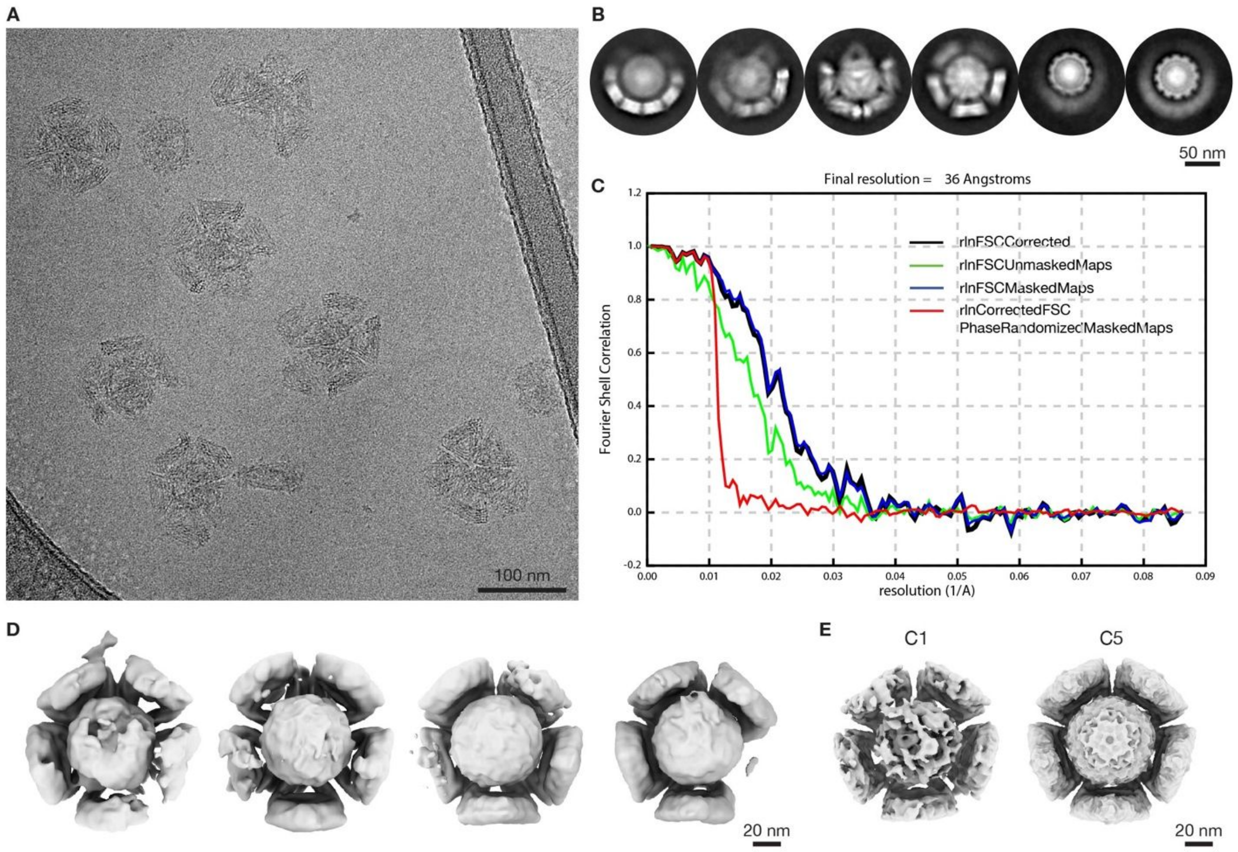

Figure S15. Cryo-EM imaging of T1 shells trapping chikungunya VLPs (EMD-13883).
(**A**) Exemplary micrograph of **T1** shells trapping chikungunya VLPs vitrified on lacey carbon grids with ultrathin carbon support. (**B**) 2D class averages of extracted particles. (**C**) FSC estimation of the reconstruction shown in e (C5). (**D**) 3D classification of extracted particles. (**E**) 3D reconstruction of **T1** shells trapping chikungunya of selected particles from multiple rounds of 3D classification without (C1) and with symmetry (C5).

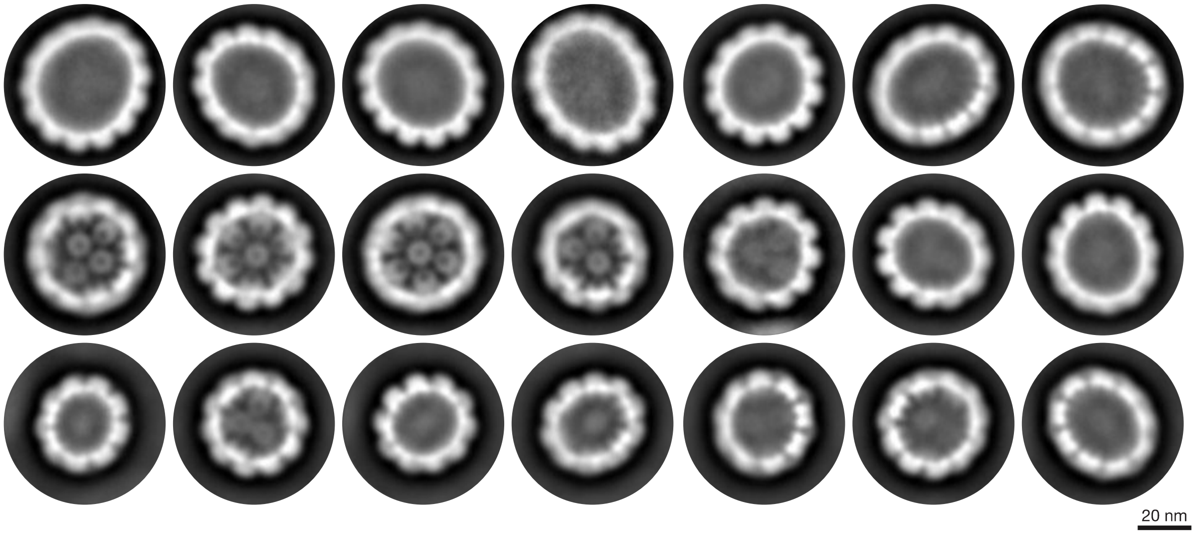

Figure S16. 2D class averages of free HPV 16 VLPs extracted from cryo-EM images.
The HPV 16 VLPs' diameter ranged from 35 to 50 nm.

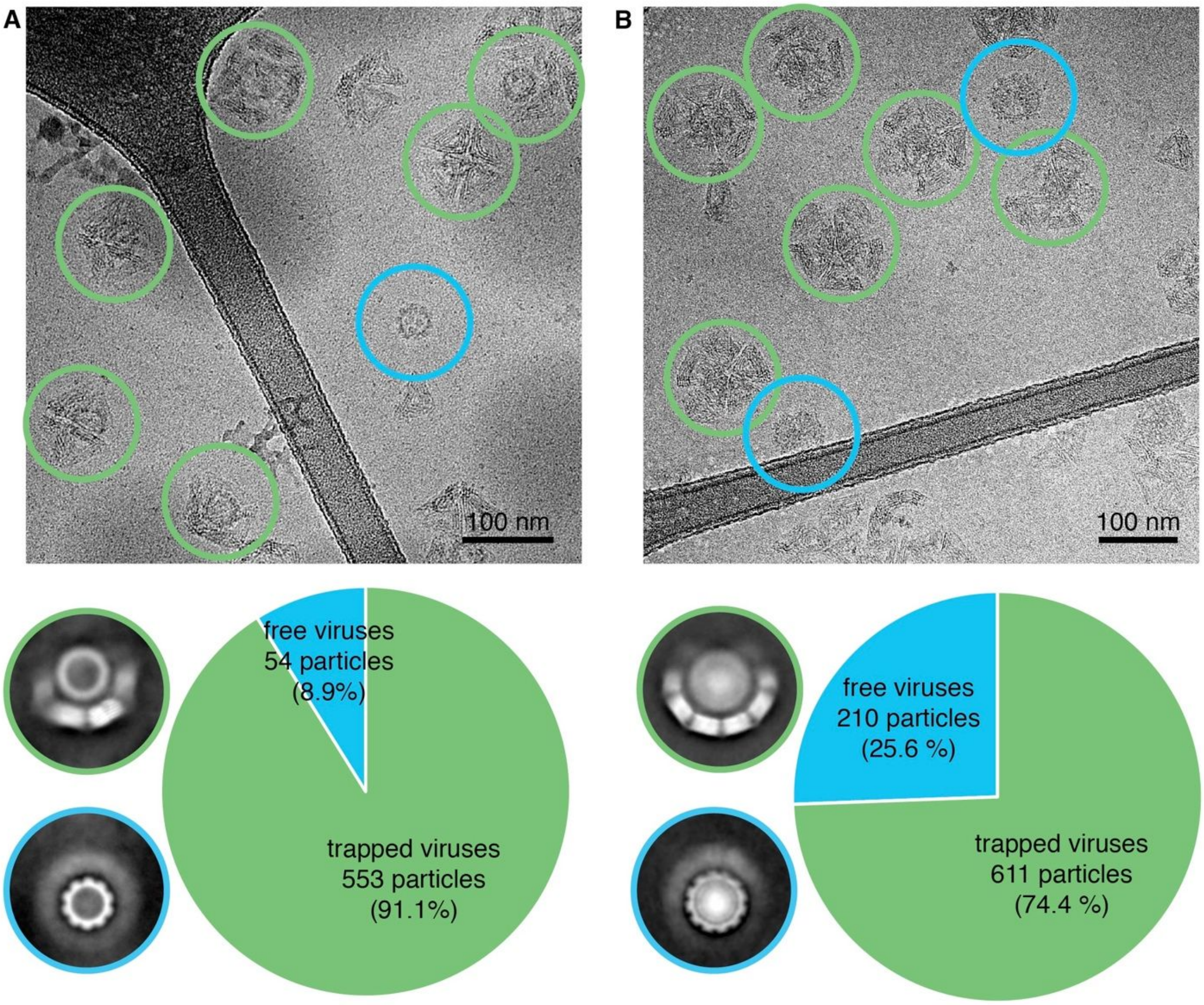

Figure S17. Quantification of trapped viral population in DNA origami shells.
(**A**) Representative cryo-EM micrograph showing free and trapped HPV 16 VLPs in **O** shells, with 2D class averages. 91.1% of the HPV 16 population was trapped. (**B**) Representative cryo-EM micrograph showing free and trapped chikungunya VLPs in **T1** shells, with 2D class averages. 74.4 % of the chikungunya population was trapped.

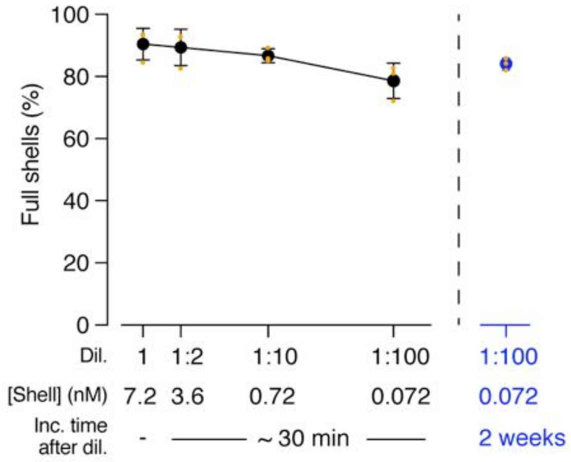

Figure S18. Stability of virus trapping by the shells.
TEM quantification of AAV2 trapping in **O** shells subjected to a dilution series. The data were obtained by automated montage collection and quantified by blind manual counting. The data is presented as mean ± s.d. (standard deviation) for n = 3 independent experiments, individual data points are overlayed in orange. One-way analysis of variance (ANOVA) with a confidence interval of 95 % was performed, comparing each of the dilutions to the undiluted sample as a control to test for statistically significant differences in number of occupied shells. No statistical significance was observed. The 100-fold diluted sample was incubated for a further two weeks at r.t. to determine if the rate of unbinding occurred, but at a slow rate. Again, we observed no significant difference in the overall level of binding.

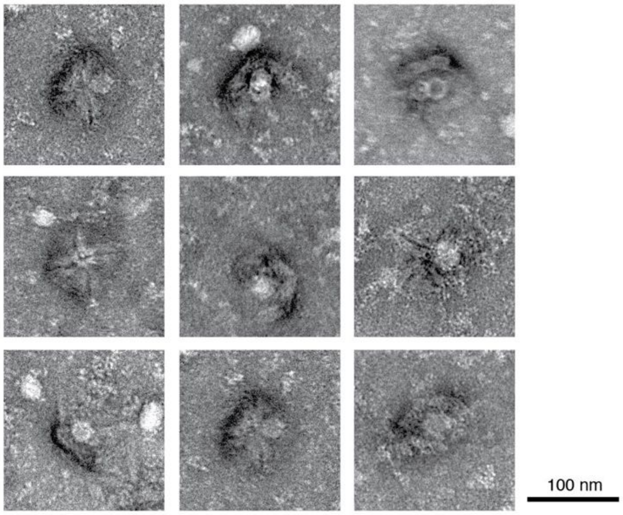

Figure S19. Virus trapping in biologically relevant media.
Binding experiments using AAV2 and **O** shells functionalized with HS **3c**, as an exemplarily system, in presence of cell culture media (DMEM, high glucose, GlutaMAX™ Supplement, pyruvate + 10 % Fetal Bovine Serum). TEM imaging of samples containing high amounts of serum is challenging, but DNA origami **O** shells trapping AAV2 particles could be observed. Scale bar is 100 nm.

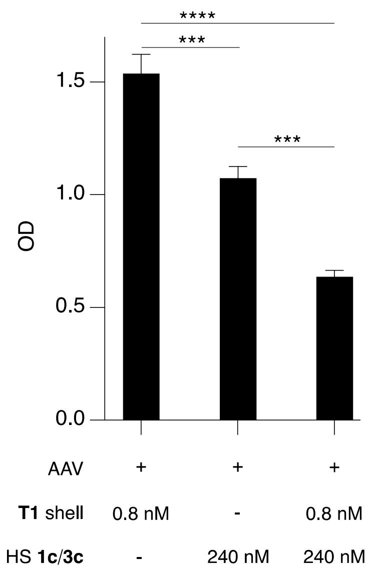

Figure S20. ***In vitro*** **AAV2-blocking via an ELISA assay.**
AAV2 particles were incubated with either **T1** shells without HS functionalization (positive control), **T1** shells modified with HS **3c** and **H3** handle design, or the equivalent concentration of free unconjugated HS **1c**. The data is presented on graph as mean ± s.d. (standard deviation) for n = 3 independent experiments, (***$p \leq 0.001$, ****$p \leq 0.0001$). One-way analysis of variance (ANOVA) with a confidence interval of 95 % was performed to test for statistically significant differences between the effective AAV2 concentration available for binding in each sample. Both free HS **1c** and HS-modified **T1-3c** shells demonstrated a significantly lower concentration of AAV2 particles available for binding when compared to the unmodified **T1** shell control ($p \leq 0.001$ and $p \leq 0.0001$, respectively). However, modification of **T1** shell interior with HS **3c** demonstrated statistically significantly lower available AAV2 concentration than free HS **1c** at the equivalent concentration ($p \leq 0.001$).

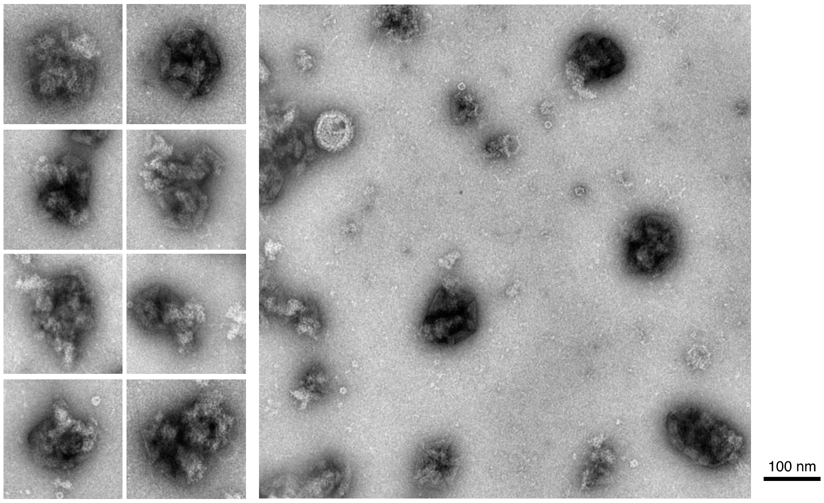

Figure S21. TEM of rubella protein debris trapping in T1 shells.
Successful encapsulation of protein debris from the rubella VLP sample.

Table S1. VLP providers and catalog references.

VLP	Provider	Reference
Poliovirus type 3	Creative Biolabs	VLP-003YF
Dengue type 1	The Native Antigen Company	DENV1-VLP-100
Norovirus G II.4	The Native Antigen Company	REC31620-100
HPV 16	Creative Biostructure	CBS-V641 HPV16
Chikungunya	The Native Antigen Company	CHIKV-VLP-10
SARS-CoV-2	Creative Biolabs	VLP-050YF
Rubella	The Native Antigen Company	REC31651-100

Table S2. Temperature ramps and scaffold used for each DNA origami triangle subunit.
For scaffold sequence see Supplementary Note 1. For staple sequences see Tables S4-S12.

**Structure**	**Denaturation step (15 min) (ºC)**	**Temperature ramp (1 ºC/1 h) (ºC)**	**Storage temperature (ºC)**	**Scaffold**
T_octa	65	60-44	20	M13 8064
T1 (pentamer triangle)	65	58-54	20	M13 8064
T1 (ring triangle)	65	65-52	20	M13 8064
T3 (6 triangles)	65	56-52	20	M13 8064

Table S3. Shells assembling conditions.

Structure	[$MgCl_2$] (mM)	Temperature (ºC)	Time
**O** shell	40	40	overnight
**T1** shell	40	40	24 h
**T3** shell	25	40	8 weeks

Table S4. Staple sequences used for the octahedron triangle (T_octa) folding.

**Core structure oligo sequences (5′-3′)**
CACGTTGAGGAATTGCGAATAATCAGATGATGAATATAC
AGGGAAGAAAGCGAAAGGAGCGGGCTGCGGCTCGTTAGATAAAGGGA
CGGGGAAAGCCGGCGAACGTGGCGGAGCTCGATTGCTTTG
CAGGCGCAGACGGTCAAACGTAACTGGCAGCCTCCGGCCA
CCCTAAAGGGAGCCCCCGATTTATTCCTGTGTGAAATT
AATTTCAACTTTAATCTTAATAAATTTTTCGAACTA
GGTTTTGAAGCCTTAAAACGCTAATTTTTGAGCGTC
ATGGAAACAGTACATATTAGATTAGGTGCTGGTAATTTTC
TAGAAAATACATACATAAAGGTGGGTATTCTAGAAGGTAA
ATAGCCGAACAAAATAGCTATCTTACCGAAGAATGGAAACAAATATTGATATA
CCAACAGGTCAGGATTAGAGAGTGTACAGACTCATTCCAAGTAGATT
TCTATCAGGGCGTAATGAGTGTTGCAGCCCTTCACCCATTTTGAAAAACGCT
ATGGCCCAAACGTGGACTCCAACGCAGCACAGACAATATT
GACTCCTTATTACGCAGTATGTTCTCCCGACTTGCGGG
CGAAAGACTTCAAATATCGCGTTGGGCTTGAGATGGTT
GAATAAGTTTATTCGAGAATGACCATAAATCAAAAATCAG
AGAAACAATAAAATTAATTTTTCGTTGTAGCAGCCTGAGTAGAAGAACTC
GCGATTATAAATGGTCAATACCGCCAGCCAGTATCGGCC
CGGAGATTTGTATTACACGAAAGAGGCAAAAGACCTCCGGCTTAGGT
AAAGACTTTTTCATGAGGAAGAAAATACCACAAAAATAGCGAGAGGCT
CCGACAATGAGCGACATTTTTAATCAAGTTTATCGGCATTTTCGGTCAT
ACTGCCCGCTTTCCAGAGCAGTTGGGAAAAAGAGACG
CATTCCAAGAACGCAACCATCCTAATTTACGAAAAGCCTGTTTAGTA
AGAACGCGCCTGTTTATATCCTGACCCAATCCATTAACTGAACACCCT
AGGCGCATAGATAAGGCTTGCCAGCAAACTAGCTTAAT
CGGTCGTTCGTGTGATAAATAAGGCGTTAAATAAGAATAAAGCCCACGCATAACCGTGA
TTTGATTAGTGCCAAGCGAAACGTACAGCTTGAGAAGAGTCAATAGTG
TGCCAACGTTTTTCAGCACCGATCAAACTTAAATTTAATCGGCC
TCAGCGTAGACGCTGAAAACATAGCGATAGCAAT
AATTTATCTTTAGTGAGTCACCTGTTTAGCTCACGACGTGGTGGAGC
AAGGCACCTTTTTACCTAAAAGAGGCTTTGAGGACTACGGAACA
AGCTTCAAAGCGAACCAGACCGGACTGACGAGAATATGCA
CGCCACGGGAACAATTCTTTTTACTAATAGTACAAGGCAAAGAATTAGCA
CAAGAAAATTTTTAATATCCATGTTCAGCTAATGCTTTCCAGA
TTACCTGAGCAGAGGCGAATTATTCAG
CTGCTCATTTGCCGCCTCGGGAAAGTTTTTCTAAAATCCTGTTTGATG
TCGTCGTATGTTTTTTAGTGTGTCCATCACGCACGGACCGAGTA
AAAATCCCGACTTTCTCCTTAGAAATCACTTATACTTC
AACTTTAATCCTTTTTATCGGAAAAGGTGGCATCGGATTTGGGG
TTAATTTCATACAGGGTAGCATTAACATCCAAACAGCTATATATT
ATCCTGAGGCTTGCAGGGAGCCGGAAACGTCGCTTGCTTATAGTTGCG
CAACATGTCGTTTTCGCCTTTAGCGTCAGAAGTAACAGCAAGTTAC
TGTAAATGGTTTGAAATACCGACCTGACCTAAATTTAATGCTG
CAGAGGCATTTTTTTTATAATCAAGGTTTAAACATCGGG
GCTCTCACGGCGGTTGGCAGCAACCGCAAGAACTTTTTA
AAGGGATAGGCAACAGTTGCGCTCTAAAGCCTGGGGTGCC

CCGTGGTGCAACAGGACGCTCAATAGTTGGCATCTAAA
GTGTTCAGCAAATGCGGTCGGTGGTGCCATCCATTTCATTTGAATTA
GGCCATCGCCTGATAACAAGACAAAGAATTTTTGCGAGAA
GCAACATCAGTTGACATTATTGAACGAGTAGTAAATTTTAATTCGGGGGTAAT
CAGCGAAAACATAACGCCAAAAGGTAACCCTCCACCATCAAATGCCGG
AAAGGTATTAAACCAACAACAGTAGGGCTTTTTTAATTGAGAATCGAGCCA
ACAATAACAGCCAGCCTAATTAGGCGTTTTAGCGAACAGCAAACGCGCTAATA
AAAGGTAATTTGTTTAACGTCAAACATAAAAATATTCACA
ACACGTTAACGGCATCAATAACCTTGCTTTTTTCTGTAAA
CAGAAGTATTGGGAACGCGCGTCATGGTCATAGCTGTGAGCTTGACTTATAAA
TTTCACCATTTACATTGGTTTTTAGATTCACCCGGTTTGC
CTGATTGCAAGCGGTCTAAGAATACGTGGAAAGGAAGGTT
GCGAACGTATAACAAAGATTGTTCATATGTACCCCGGTTGATAAT
GCTCCATGAAATGCAACATAAAGCTTTTTTAATCGGTTGTACCAAAAA
AGATTTTTTTAGGAAATCTACGATTGTGAATTACCTTAAGAAGCAA
AGCGGTGCAGTCACACTCCAGAACATTTTTTATTACCGCCAGCCATTG
TTTGCCATCAACATGTTTTAAAACACCAACAGGTAGTTACTTAG
GCAAGCCGCAGAACCACTTTTCATATTTTTTCAAAATCACCGGAACCA
GGTAATTCGATTGAGGGAGGAGAACGCGTGCCAGTTTTTTTATT
CGTTCCGGGACCCCCAAAAATCATACCGGAAACAATCGGCTT
AGGCTCCAAAAGGACCATCAAGAGAAGGATACCGCCAC
TTATCCGCTCACAATTGCCAGCTGTTTTTATTAATG
ATAATACATTTGAGGAAGCAGCAATATTAATTTTTTAGACAGGAATAC
GTCACCGAAAATTGTCACAATCAATAGAAAATGCCCGTAAACCTATT
TATCACCTATTTCGGTAAACAGTTAATGCCTTCTAGCT
AGGTGAATAATTAGAGCATAGTTAGACGTTAGTAAATTTTA
ATATCTTTAGGAGCACTTCTGACCATGGATTAGTGAGACG
GGCAAAAGCGCGTACTATGGATTCGTAAGGGAGAGGCGGTGCCC
GAGTGTTGGGGGTCGAGGTGCCGTAAAGCACTAAATCGGA
TTCCAGTTTGGAACAATCTTTAATTAGAACCCTAACAACTTTTTAATAGA
TATTAAAGCTACGTGAACCATCACCCAAATCACCGGAAGCAGCAGGCG
CGCCTTTTTGGGTGCCTGTCGTCCACACAACATACGAGAGTTTTTT
CGACCTTTTGATAAGAGGTCATTCATGTCAAATAAGCAA
TGCTGAATAGAGAATCTGCCTGAGGAGTAACATGTTAA
AGTAAAATGTTTTTTTTGACTGGATAGCGTTTTGCTTTTTAAAGAAGT
CCAATACTAGCGGATTGCATCAAAAAGATTAAGAGGAAGC
GCGGAATCGTCATAAATGAGAGATGAGAAAGGCATTAAATTTTTGTGAGC
GAATCCCCGTCTTTACCCTGACTATTATAGTCTGCGATTTTAAAAACC
CTCAAATGCTTTAAACGATAAATTATATGATAGTCTGGCC
AGTTCAGAAAGAGCAACACTATCAAATTACGAAAGCCTTTCCCTGTAA
TTTTGCGGAGCCAGTAAAACAGGAGGCCGATATCAGAG
AACAAAGATTGAGTAACAACTCGTTATTAGACTTTACAAACGAGAGGG
GACAAAAGAGTTTTAACCACCAGAGGTTTAGTTAGGAT
TCAGAGAGATAATTTTTCCACAAGAATTGAGAACATTTTTAGTCAGAG
GTTAAGCCTAACGGAATACCCAAAAGAACTGGCATGATTA
CAATAATAAGAGCAAGTCCAGTAAGGCAGGTCCACCGTATTTTTTCAGGA
AATTAAGCAATAGGGTTTTCCCAGTATATTTTCATAACCTCAGGTCTG
AGCCCTTTTTTACAGAGAGAATAAAATGAAAACCGCCACCACCGGAAC
TTAGGCAAGTGTAGCGGTCACGCGGCGGTCATGAGAGCC

TCAAAAGAATAGTTTTTCCGAGATAGGGTTGTGGTTTTTTCCGAAATC
TTTGAATGGCTATTAGGAGTCCACTTTGCCCCATAAAGTG
ATTGCCTGAGAGTCTGGAGCAAACAATAATGCTGTAGCGAG
TGTTGGGAAGGGCGTTTTTTCGGTGCAGGGGGATGTGCTG
ATGATACAGGAGTGTACTGGTAATAGGCGACATTCAACGAG
AAGCGCAGTCTCTGAATTTACCGTAAACAATGCGCATTAGACGGGAGA
CGAACCACGACCAGTATTAGAGCCAACCCT
AAACATCGCCATTAAAAATACCGAAGCGCCGCTACAGGTCC
CGTTAATAGATGAACGGTAATCGTAAAACTAGTTTGCGGATGGAAGTT
TAATGTGTAGGTAAAGATTTTTTCAAAAGGGTCTACAAAGGCTATCAGGT
AAGGCGATTAAGTTCAGCGCATTAAATTTTACCCGTCG
TTCAACCGGCGGGAGGGCATAGTCTTTTGCGGGATCGTC
GTATTAAGCGGGGTCAGTGCCTTGAGTAACAG
GCCGCCGCCAGCATTGATTTTTAGGAGGTTGAGCGTCATACATGGCTTTT
AGACGATTTCAGAGCCAACGATTTAGTAATTCTGTCCAGACGACG
AATCCTCACTCAGAGTAGCAGCCATAAGAGAATATAAAG
TGCCACGCGTATTAACACCGCCTTTAAAGCC
TCTAAAGCCAGCAGAAGATAAAACAGAGGTGATGCGCGTATGCTTTCC
ATAAAAGGGACATTCTGTTTTTCCAACAGAGAGCGCGAACTGATAGCCCT
ATACCTAGCCTGGCCGAGAGATAGTAAAAAAAAAAGAACG
GACGACAGCTTTCCGGCACCGTAACGCCAAAGGAAAGATACA
ATTTCAACACCAATATTCCTGTAGCCAGCTTTCATCAA
GCGTAACGACAAACTACGCCACCCTCAGAGCCTAATTCGC
TTAAAAGTAACCACCAGAAGGAAAGAAATTGCGGTCGGTACGCCAGAA
ATCGCACTGAATTTGTCCAATTCTACTGACCAACTTTCTAACTCACAT
CCTCAGAGTTTTGTTAAAATTTTTATTGTAAAACGGTGTCTGGCTTAG
TTTTGTCGAAAGGCCGCTTGAGCCATTTGGG
ATTGGCGCTCCAGTAGCACCATTACCATTAGCAAGGTTTCTTTCCA
TTTGCCATAGGCTGAGACTCCCAGACATGAAAGGAAATTA
TCAGCTCATTTTTTAGCAAGGATAGTCAAATGTTTACCA
GGAACGCCATCAAAAATACGCCAGTACTTTTAATAAATC
TGGTAATAATCACCTTGCTGACGTATGAAAAAGTATAACGACCACCAC
AGCGGGGTTTTGCTCCGCCACCCGGCCTTGACAGGGAAG
GCCCCCTTATTATATCGGTTTATCAACCAATGACAACCATCCACCGGA
AACTATCGGCCTTGCGTAGATTTTCGTGAGGCCATTCGCCTAACAAAA
AGTACCAAGTATAGCCCGGAATAGGTGTAT
AGAATAGAAAGGAATTTTTAACTAAAAAATCTCCAAAAAA
AATCCTGATTGTTTTTTTTGATTATAATATCAAAATTATT
AACCTACCCTTCTGAATAATGGACATTCGCCGACGGCCA
GCACGTAAAACAGAGAAACCTCAAATATCAGTCAATAG
AATCAATATCTGGTCCGTCTGAATGAAAGCGCACGCTGG
AAATCAACAGTTGAAAAGGGTTAGCATGGAAGTAATAAC
ATAGGTCATCAGGAAGAAACAGGGTTGATTC
CGTTGGTGGATTGACCGTAATGGGGGAATTGAAGCCCCAA
CCTCAGAACCGCCACCATTCTGAAACAGCCCTCCAGCA
CTCAGAACCAACGCCTGTAGCATTGCCGAATTTTCTGTATGCGGAGTG
CCCAATAGGAACCCATGTAGCTAAACAACCACCCTATCTAAAGCCCTGCC
TTTCGTCACCAGTCATTTTCAGGGATAGCAAG
GGCGGATAAGTGCCGTAATTTTTTCGCCTCCCTGTAGCG

GATTCTCCGTGGGAACATATTTAAGAGGGGACTAGTTTGACCATTATC
AAACGGCGTAGACCGTGCATCTGCGGGCTTCTGGTGCCGGTGCGCAAC
CAATTCGACATTATCAGCAACAGCGGGAGCT
TTTAGAAGATTAAATCCTTTGCCCAATAGCGGAATTATCAATCAATAT
TATTTTTTTTTATATCTTACCATCAAGATTAGTTGCTCGCAATAA
AAAACACTGCGAAACAAAGTACAACCGGAACGCGCGACCTACTAAAGT
AAATAAGAACCACCCTCATTTTTAGCCGCCACACAAAATA
TTCAACTATTAGAACCCTTTTTTATATATTTTAAAGATTC
AAATCACATCTAGGAATCTAGAAGGCTTATCCGCAACATATGCGCCAAA
CTGGCGAAGGGCCTCTTCGCTATACTTTCAAAATTTCTT
TCAAAGGGCTGAGAGAGAGGAAAGAGGACAGATTGACAAG
TTTCGCACCAAGCCATCTTTCAAACGCGGTCCGTTTTTTAAAT
TAATGGACTTGTGTTAAACGATGCTGATTGCGAGCACATAGGCGGCCGGAACCGA
TGGTCGTCTCGTCGCAAAGCTGCTCAATTT
TCTCTTCAGTGAGCTGGCTGACCTTCATCAACCCAAATCATCATAAG
TATAAACCGGATATTCATTAGAGTAATCTGAACGGTACCTTTAATTGCTAAAACCG
TCCGCCATGTTTACCATGAAGGGTAGCCGCACCCTCATAACGGAACGTGCCAATT
CGCTACCGTAATCAGTACAAAACCATCGAAACCAATCT
TTCATATGTTCATTAAATCAGATAATTACCGCGCCCAT
TATACGTAATGCCACTACGGGGTTATATAACTATA
TCGGCTGTCTTTCCTTATTTCATCGGAGAACAAATATTGAC
CCGGAGACAAAAATTTATGCAGATGACAGCATCCATTAAACGT
TATACCAGTCAGGACGTTGGGAAGTTTCGGAACGAGGGTA
AGAGGGTAGCTATTTTTATTCATTGACGACGATAAGAACTGGT
GTTTACCAAAAAGAAACGCAAAGACACCACGGCAAGCAA
TTTTTTAAGTCCTGAACATATGCGTTATACAAATTCTTACCATTTCAA
AATTTAGGAAAATCGCGCAAAAGAAGATGATGAAACAAACGCTGGTAATGGTTTTT
TTTTTCACTCTGTGGTGCGCTAGGGCGCTAGAAAAGTAAGCAG
TTTTTGTAAAGGTTTCGCGCGCCTGTGTTTTT
TTTTTAGCTACAATTTTCAACAATAGATTTTT
AAATAGCAGTTACCAGAAGGAAACCGAGGAAAATTTTGCACCCTTTTT
**H1 design oligo sequences (5′-3′)**
GCAGTAGAGTAGGTAGAGATTAGGCA<br>CAATACTATTAATTCTGGTCATGTACATCGACATAAATAATTGCG
GCAGTAGAGTAGGTAGAGATTAGGCA<br>TCAGATGTAAAACATTCAGGCAAACCAGGCAAAGCGC
GCAGTAGAGTAGGTAGAGATTAGGCA<br>AGAGACTACCTTTTTAAATACACTCGCGAGCTATTGTGTC
GCAGTAGAGTAGGTAGAGATTAGGCA<br>ATGCATTCAAATATATTTTAGACCCTCAGCATTATGA
GCAGTAGAGTAGGTAGAGATTAGGCA<br>TACCGTCGAGGTGCAGTTTCAGGGATTTTCCGTAACACTGAG
GCAGTAGAGTAGGTAGAGATTAGGCA<br>ATCATAATTACTAGAAGCATGTAGATAGCAGCGTACCGCA
GCAGTAGAGTAGGTAGAGATTAGGCA<br>TATAACCATATTTAACAACGCTACCGACAGAGCCACCCTCAGAAC
GCAGTAGAGTAGGTAGAGATTAGGCA<br>CTTTTCGTCAGATGGCAATTCTCATATTCCTGATTAT

GCAGTAGAGTAGGTAGAGATTAGGCA<br>TTAATTACATTTAACACACGCAACAAAGAGTCAGATGCCG
**H2 design oligo sequences (5′-3′)**
GCAGTAGAGTAGGTAGAGATTAGGCATTTTTTTTTTTTTTTTTTTTCAATACTATTAATTCT<br>GGTCATGTACATCGACATAAATAATTGCG
GCAGTAGAGTAGGTAGAGATTAGGCATTTTTTTTTTTTTTTTTTTTTCAGATGTAAAACATT<br>CAGGCAAACCAGGCAAAGCGC
GCAGTAGAGTAGGTAGAGATTAGGCATTTTTTTTTTTTTTTTTTTTAGAGACTACCTTTTTA<br>AATACACTCGCGAGCTATTGTGTC
GCAGTAGAGTAGGTAGAGATTAGGCATTTTTTTTTTTTTTTTTTTTATGCATTCAAATATAT<br>TTTAGACCCTCAGCATTATGA
GCAGTAGAGTAGGTAGAGATTAGGCATTTTTTTTTTTTTTTTTTTTTACCGTCGAGGTGCAG<br>TTTCAGGGATTTTCCGTAACACTGAG
GCAGTAGAGTAGGTAGAGATTAGGCATTTTTTTTTTTTTTTTTTTTATCATAATTACTAGAA<br>GCATGTAGATAGCAGCGTACCGCA
GCAGTAGAGTAGGTAGAGATTAGGCATTTTTTTTTTTTTTTTTTTTTATAACCATATTTAAC<br>AACGCTACCGACAGAGCCACCCTCAGAAC
GCAGTAGAGTAGGTAGAGATTAGGCATTTTTTTTTTTTTTTTTTTTCTTTTCGTCAGATGGC<br>AATTCTCATATTCCTGATTAT
GCAGTAGAGTAGGTAGAGATTAGGCATTTTTTTTTTTTTTTTTTTTTAATTACATTTAACA<br>CACGCAACAAAGAGTCAGATGCCG
**H3 design oligo sequences (5′-3′)**
GCAGTAGAGTAGGTAGAGATTAGGCATTGCAGTAGAGTAGGTAGAGATTAGGCACAATA<br>CTATTAATTCTGGTCATGTACATCGACATAAATAATTGCG
GCAGTAGAGTAGGTAGAGATTAGGCATTGCAGTAGAGTAGGTAGAGATTAGGCATCAGAT<br>GTAAAACATTCAGGCAAACCAGGCAAAGCGC
GCAGTAGAGTAGGTAGAGATTAGGCATTGCAGTAGAGTAGGTAGAGATTAGGCAAGAGA<br>CTACCTTTTTAAATACACTCGCGAGCTATTGTGTC
GCAGTAGAGTAGGTAGAGATTAGGCATTGCAGTAGAGTAGGTAGAGATTAGGCAATGCAT<br>TCAAATATATTTAGACCCTCAGCATTATGA
GCAGTAGAGTAGGTAGAGATTAGGCATTGCAGTAGAGTAGGTAGAGATTAGGCATACCGT<br>CGAGGTGCAGTTTCAGGGATTTTCCGTAACACTGAG
GCAGTAGAGTAGGTAGAGATTAGGCATTGCAGTAGAGTAGGTAGAGATTAGGCAATCATA<br>ATTACTAGAAGCATGTAGATAGCAGCGTACCGCA
GCAGTAGAGTAGGTAGAGATTAGGCATTGCAGTAGAGTAGGTAGAGATTAGGCATATAAC<br>CATATTTAACAACGCTACCGACAGAGCCACCCTCAGAAC
GCAGTAGAGTAGGTAGAGATTAGGCATTGCAGTAGAGTAGGTAGAGATTAGGCACTTTTC<br>GTCAGATGGCAATTCTCATATTCCTGATTAT
GCAGTAGAGTAGGTAGAGATTAGGCATTGCAGTAGAGTAGGTAGAGATTAGGCATTAATT<br>ACATTTAACACACGCAACAAAGAGTCAGATGCCG

Table S5. Staple sequences used for the T1 pentamer triangle folding.

**Core structure oligo sequences (5′-3′)**
TGCGGCGGCCGGGTCAGTCCAGCATCAGCTCGATAACGGA
TGCAGCAAGCCTGGGGTGCCTAATGAGTGAGCTTTTTAACTCACATTAATTG
ATTTCATTTGAATTACCTTTTTAAGAAGATGATTCATTT
GGAGGTCAATAACCTTTTTTTTATAGTAGTAGTTTTATTAATAGAT
CTCACAGTGTTTCTGCACAACTAAAGGATTTA
AAAAGAATTAAGTACTATGCCGTACTGGTAATAAGTTTTAACTTGCGTA
TACCTACATTTTGAAAGGGACGAATGGCTCGCTTAGGAGCACTACAGCACG
AAATAGTTTGACCATCATCCAATAA
GTTAGAACTCAAACTACCTGAAAGC
TATGACCCTGTAATACTTTATAAAGCCTCAGA
GCGCGAACTGATAGAAGATAATGCTGAACCTCAATTTTAAA
ACCACCAGAGCCGTTGATATTTGATACAGGAGTGAGTAAA
GCATAAAGCTAAAAGAAGCCTGTGAGAAAGGCCGATTGA
CGGAACAAAGAAACCAAGCCCGGATCAAGTTTCCGGTTTTGCTCAGT
CATTTTGTATCATCATATGGGTCGAGGTTTTTTCCGTTCATTTGC
AATATTTGCATTAAATTGTTTAGACTGTTTTTATAGGCATCGTA
GTGTACAGACCAGGCGCATAGGCTATGCCACTGAGGCGCAAAACAGCT
CAGCTTTCATCAACATTAAATGTGAGCGAGTCAGCTCAT
GCCGGAAACGTACCCCGGTTGATATATAAGCA
TACTCAGCCCTCAGAACAGAGAGATAATTTTTCCACACGCCAGG
GAGGTTTAGTACCGCCAAAACAGGGGGGCGCGCTTTAAAA
TTTTTAACTATTTTGTTGCTTTAAATTCACC
ACAGCGCCCACCGGAAACAATCGGAAGGTGCCGTCGAGAGTATCACCG
GTGCCAGCTGCATTAATGAATCGGCCAACGCCAGGGTGGTTTTTCTT
CGCTTTCCAGTCGGGAGTTAACGGTGGTGGTTACAAGAGTCCACAATCCGCCGGGC
CCTGTAGCCAAAAATAATTCGCGTAAATTAAATCCTTTGCAACATTAT
CAGAACGATCAACTTTAATCATTGAGCTCAACAGCTTCAA
TCACGACGTTGAGCGCTAATATCCGCAAGTCAGATTGAATA
GGAATTTGTGAGAGATCAGATGATGGCAATTCCCAGAAGGGGGGAAAGTTTGCCA
ACGGGTAAAATACGTAGGCTGACCACGTTAATGACGGTCA
ACGAAGGCGCGCCGACAATGACAAGCTCCAAAAGGAGCGCAATGAATT
AGAGCCACCACCCTCAACCCTCAGATAGCTATAGCTAGCAAGGACCAT
TGAGAATCGCTTTTTATATTTAACTACCTTTTTTTTTAACCTCCGG
CGCAACTGCGCAGAGGGAATTAACTGAACACCAAATAGCAAACCGCCA
CCAGCGGTGCTTTTTGGTGCCCCCGGTATTTTTTGGGTAAAGGTTT
TGCATCAGACGATCCATGTAAAGCGGTCCACGAATCATGG
CATGAGGAAGTTTTTTTCCATTAAATAATTTTTCGATATATTCGGT
AATAGCAAGCAAATCAGATATAGTCAAATATATCCCAATCCAAAGAT
ATCGTAGGAATCATTATATAAAGCGCCAGTTAATAGCAGCCTTAGACGCTGAGAAG
AGGCGGTTAGAAACCAATCAATACTAATTTACGAGCATG
CTGATGCATTAAAATTCTTACCAGCCGCGCCCGCCTTAAATCAAGATT
CTTAGGTTAACAGTAGGGCTTAATAGCCGTTTCCAGCTACAA
CTTTGCTCGCCGGGTTACCTGCAGCGTTGCGCCTGAGAGAGT
TTCTAAGAAGAGGACAAGAGGCAACCGCGACCTACAACGGGGCTATCA
AAGGCTTAGCGAACCTCCCGACTTACATTCAACAAACGTA
TTGCGGGAAACGAGGGTAGCAACGAGTGAATAGATTTTAAGAACTGGC

ATCGGCTGTCTTTCCTTATCATTCTTAGGCAGTAAGTCCTGTGAATTT
GTGAATAACCTTGCTTTGTAAATGAAATGAAACAAAATAAAAGGTGGC
CGTCGCTACAATAACGGATTCGCCCTATTACGCCAGCTGG
TTAATTAATTTTCCCTTTTTTAGAATCCTTGAAAACCATAGGTCTGAGAGAC
CGGTGGTGCTGGTCTGGTCAGCAGTAGCTCTCACGGAAAA
CCATCCCACGCAACCATTTTTCTTACGGCTGGAGGTCTGTTGCCCTGCGGCT
AGCCGGAAGCATAAAGGCGCAGTGGAATTCGTGGTATGAGGCCGTTTT
GAATAATACAGTTTCAGCGGAGTGGGAACAAACGGCGGAT
ATTTTTTCACGTTGAATTTTTATCTCCAAAAAAAAAGCAACCATCGCCCACGC
TTCTGTATTAGGTCACGTTGGTGTAGATGGGCCCAGGCAA
TTTCCAGATCCAGCCATCACCAGTAAACAAGAGGTCATTG
GATGAATATACAGTAAGCTGCAAGGCGATTAAGTTGGGTACGAAACGT
ACGTGCCGAGCGGATCAAACTTAAATTTCTGCCTGGCCTT
AGGCTTGCCCTGACGAGAAACACCGAAAGACCACATTCAACTAATTC
GATCGCACCGTTAGTACTGTAGCATTCCACAGGCGATTAT
GCTTTCCGGTCAATCATATGTCCAATACTGCGGA
GTAAAACGACGGCCATTTTTTGCCAAGCTTTCAGGTTTTCCCAG
ACAAAGCTGCTCATTCGCTACAGAGAAAGATTAGTAAGAGCAATGCTTTCGAGGTG
TAACTGATTGTTTGGATTTCAGGGCGATGGCAGCTTGACAGCGGAAT
TGTACATCGACATAATTTTTAAAATCCCGTAAAACGCCAGCAGT
AACGCCAACATGTAATCAAGAACGAATCTTACGAACAAGA
AGGGGACGACGACAGTTTTTATCGGCCTCAGGAAACCGTGCATC
TTAGACGGGAGAACCCGAAGCCCTTCCTTATTTGCAGCCA
GTCGGGGTCATTGCAGGCGTTTTTTTTCGCACTCTA
GTTTTTTCCACGGTCAGGCCAGAACGCCTGTGCACTCTGTTTCCACAC
TGCCGTTCCGGCAAACCTTTAGTTCGACAACTCGTAGCACTAAATCGGA
AATTTCTTAAAACGAACTAATTTTTGGAACAACATTATCCAGTCAGTCAACGTA
TGATACCGCAACCTTTAATTGTATCGGTTTTTTTATCAGCTCAC
AGATTTGTAACACTCATAGTTAGCGTAATTATGAAACA
ACCAAGTTACCAACCTAAAACGAAGATGAACGACTGACCAACTTTGAA
AGTCAATACAACGCTAACGATTTTTCGTCTTTCCAGAGTTTTGCACTTATTTTC
AATATAAAATTATTTGCACGTGCGATATTTTTCTTAGATTATAC
CTGTCCATAATGGAAGGGTTAGGGAACGGAACCAGGCGGATAAAATTGAGTTAAGC
GACGACGACAAAGCCCGAGATAGGTTAATGCGGAGAAAGG
GTTGAGTAAAGGGCGAAAAACCGTCTAATAAACGTGGC
GCGGTTGCCTGGTTTGCCCCTTTTTGCAGGCGAAAATCGCCTGGCCTCACTGCC
TGTTCTGCAGATACATAAGAAAGACTGAGAATGA
CGCCAAAGACGATAAAAACCAAAATAGACGCAGAAAAC
CAAAGAACCCTTAAGAAACGATTTATTAAGACTTTTAAGA
CCTAATTTCAACGCTCGGGTTATATAACTATACTGTAAATAGAGAGAA
TTCAGCTAATGCAGAAAGTAATTCTGAAACAGAAGGATTACCACCGG
TATGCAACTAACAGTTGAAGCGAACGAAGCCC
AAGGAGCGCGTAACCACCACACCCACGTATAAGGCAAAATGTGAGACG
CATATCCAGAACAATTTTTTTTACGATAGAACCTTTTTTTCTGATCGG
TACAGGTAGGCTTTGACTTGCAGGGAGTTAAAGGAATTGCTATCATAA
AGAAAAATCTTTCATCAAGAGTAATCTTGACATTTTTGAACCGGATATTCAT
TTTTATCCTGGGTATTAAACCAAGTACCGCACTTTTTCATCGAGAACAAGCA
CCGGAATCATAATTACATTTAATGAAACTTTT
TGTGAAATTGTTATCCATCTGGTCGAAGGTTAGTGAGGCGACAGACAA

CGCTGAGGGGACTAAAGACTTTTTACCCAAAGACGTTGGGA
TGATAAGAGGTCTTTTTTTTTTGCGGATGGTCATTATA
AATATGATGAGAGGGTAACGCAAGCAAAGAATTAGCAAA
CCAGCGCCCGGAAATTCGCAGTCTCTTTTTGAATTTACCGTTCCAG
ATGGTTTAAACATATAAAAGAAACGTTTTTAAAGACAC
TTTGTCACAATCTTTTTATAGAAAATTCATAGTTGCTA
CTGTTTGACATCAGATGTCATAAACATCCCTTAGCACCGTTTAAAGAA
GGGGTCAGAATGCCCCAAATAAATCTCAGAGCCACCACC
TGAGGCCAGTTGCTTTGTAATAACATCACGCCCCGCCAGCATTGACAGGAG
CTGAGAAGATTAACCGAGTGCCACGTTTTTTGAGAGCC
AAGGGATTTTAGTTTTTCAGGAACGGTACGCCTTCACC
AGTTGGCAATGAAAAATCTAAAGCATCACCTAACAGAG
CTTAGAGCTCCAACAGGTCAGGATTTTTTTGAGAGTAC
CTTTAATTTAGTCAGAAGCTTTTTAAGCGGATTGCATACCCTG
TAATGCCGATTCAACCTGTGTAGGTAAAGATTCAAAAGGTTATTTC
AGCTATTTTTGACAGAAATTGTGGCGTTTTATCCGGTA
TATGTTAGCCGGAGACAGTCAAATCACCATCACGCGAGTCGAAAT
CACGGAATAACCGAGGAAATTTTTGCAATAATAACGAGTTACC
AAACAGTTTGCCTTGATAAGCGTCATACATGGCTTTTGACACAAAC
CTGCCTATTTCGCGCACAACATGTTGGGCGCGCGGGGAG
GAATCAGAGTTTTTGGGAGCTAAACAGGAGGGCAAGTGTAG
TCTAAAATATCTGTTGCGTCCGTGTTTAATTGTAGTAAATTGGGCTGCTCACAA
TTAATTCGATGATATCAAACCCTCAATCAATTGAGATGGAGCCTC
AAGCAAACTTAATTGCGTCTGGAAATATTTTAATTTTTTGCAATGC
CCGCTACATTGTTGCCTGAGTAGAGAGGCAGGGCATTTTCGGT
GGTCACGCTGCGGGCGCTATTTTTGGCGCTATAGATAA
TCAAATATCCAGAACGAGTAGATTAATACCGATCGTCTGAAAT
ACTATTATCTGGAGCACAAACTAATAGCGCGAAACAAAGTGCTCCAT
GAATCGATTCTACTAAGCTATATTTTCATTTAAGATTGATCAGAA
CCGAACAAGAATACCCAAAAGAACCATACATAACAGCCATGTTTTGAA
AGAAGGACTGAGACTATATCAAAGTACCGACAAAAGGTAACGCGCCT
GCAACGACCAGTAATAACGCTCAAACGAACCAATTAGTCTTTAAT
CAGGTCTTTCAAAAAGATTAAGAGCAGACCGGGAGATTTACCTTATGC
CTAGCATGCAGCAAGCCCAATAGCGAACGATCTAAAGTT
CCAATAATAAGAGCAAGAGCAGATAGAAACAGGGAACGTCAA
TTCCCAATTCTGCGCAGCCCTAAAACATCGCCATT
CCCTCAAATAAAATTCAAATTGTAAACGTTAATTTAAAAG
ATCGTCATAAATATTCATTCAAAAATTACCAGACAGGAATTA
CGATAGCAGCACTTTTTGTAAAACCGCCTCTTTTTCTCAGAAATC
TCAGTAGCGACAGAAATAGGTGGGTTGAT
TCTTTTAATAGCCCCCTTATTAGCGGCCTTTAGCGTCA
CAGGAAAAACATTTACAAACAATGATGAAGACGCCAT
AGTCACAATTTATTTACATTGGCAGACGCTCATGGAAA
CATCAATGAACGGTAATCGACCATGTACCGTATCATCG
GCACCATTACCATTGAAAAGGTGGATTAAGCAACGGAGATCTACAAA
ACCAGTAAAAGTAAAACAATGCTGAACACACCCTCGGCGATC
CAGAGCCAGGATTAGCGGGGCGCTTCTGAA
GGTCATAATCAAAATCTTTCATCGTCAGACGACGCCACCAGAACC
CCTTGCTAAGGGAAGATTTAGCCACTACTGATTATAGACTTT

CCAACAGACGCCAGCCATTGCAATTTGAGTCCGAACG
CGTCACCGACTTGGGGTCGGTTGTACCAAAAACAT
ACATTTCCCATAAATGAATCCCAAAAGAGTTAAATAACAACC
ATCATACCCTGAGAGTGATAAATGTTACTTAGGAACCGA
AGGCAAGGGATAAAAATTTTTAGAACCTTTTTTCATGTTTCATT
TTTGGGAATTAGAGCCAGCAAGCCGCCACC
CTCAGAATTAAGAGGGCCCGTATGTTTATCAATCCCATC
CCGCCACCCCTCATTAAAGCCAGAATGTTTTTAAAGATTCATTA
GTAAGAAAGCCGTCAGTTGAAAGCCCGGGTAGTTTCCTGAACATACG
TATTTTTATTCTGGGAAGTATTAGACGTTATGCTGATCGTGCC
CTGAGTAAGTTCTAGCGCTCCTTTATCATAAGGCCGGAAC
AAGGTGAAAATATTGAAAAGACAAAAGGGCGGCGGGAG
GTCAGTATTAACACCGCCTTTTTTCAACTTGTAGCAATACTTCT
GACTGTAGCGCGTACCGGAACCTCAGAGCGGGGAACCTATTATT
CCATATAAAGTACGGTTGAATATAATGCTGT
AGAGTCTGTCCATTGATTAGACGAGCGCCGCGC
GGGAGGGAAGGTATTATCACGAAAATATGGCATG
TCACGCAATGTTTTTATAATCAGTTCACCACCCTTATAAATC
AGCAGCAAAATCAACAGCCGATTATCATAGCTCCGAGCTCTCACTGCG
TTTGTTTAAGCGCATATGTGACCAAGTTAAGTAC**T**
**T**TATACATTTCATCTTCTGACCTAATAGAAAAATCGCAAGACAATTACC
**T**GTTAAATAAGGACCCCCAACAGCCCTGAGTTTCG
ATTATTTATTTTAGAATCCAAGCCTGTTTAGTATCAT**T**
AAGAATACACTAAAGAGTTTGAAATACCGACCGTGTGATAAA**T**
**T**CAATATGAGCAAAATGGAAACCAAAATCGTTGGGAAG
**T**ACATTTAACATTGTCGTCAGCGCCATCTTCTGGT
GCGAACACTCATCTTTAATAAACAAACATCAAGAAAACAAAA**T**
**T**TAATTCAGCAAATCAACCTGTCGGCAACAGCTGATTGCCCAGAATCCCTCGTTA
**T**ATGCCAGTAATAAGAGATCAAAATATAAAAACAGAAATAA
AGGTGGAGTAACGTCAAGAAATTGCGTAGATTAAT**T**
GTAACAGTAAGTTTATAAAATAATACAATAGAAGGCATTTTCGAGCGT**T**
TGGGCGGTTGATCAAGTTTTTTGTCCGTGAACCAAGAATT**T**
ACCCTAAAGGGAGCCCCCGAAAGCGACCAACGTCGTTGTTCCGTGGGC**T**
**T**TTAATGCCAACGGCACACTGGTAGTTTGGACCGAAATCCGTGCTTT
**T**ATATTCAGGTTCCGCCACGAACCTACCCCTCAAGATGAAAGTA
AAAGCCGCACATCCTCTCGCTGGCAGCCTCCGTTT**T**
TGCCAGTTTGGTAATAGTAAAATTTTAGTTTTGCAAGCCA**T**
TGAATTAGGAATACAGCATCGGTCGTCACCCTCAGCAGCAG**T**
**T**TGCGCCAGAGCACAGGCGGCGCGGTCCTACATTTGTAGATTAGTACGTGGC
**T**ACGGGTGCTGCTACCGGGGTGAGGATCGAATTGAG
**T**TAAGAACAACTAAAGGCCGCTTCGAGGCATCATCAGTT
**H1 design oligo sequences (5′-3′)**
GCAGTAGAGTAGGTAGAGATTAGGCAAGAGACGTTTTTAGAAACGACTTGTAGAATTTTTGTCAGCGTGGTG
GCAGTAGAGTAGGTAGAGATTAGGCACGAAAGGTTTTTGGATGTCAGTACCTTTTTTTTCATCGGGAGAAA
GCAGTAGAGTAGGTAGAGATTAGGCACGTCGGATTCTCCGTGAGAATAGACAGAGGGGCCCTCGTT

GCAGTAGAGTAGGTAGAGATTAGGCACTCCGTGGTGAAGGGACAACCGCATCACCCAAC GTGGACT
GCAGTAGAGTAGGTAGAGATTAGGCAATAAGTATATCAATATAATCCCTATGTTTACCAG TCCC
GCAGTAGAGTAGGTAGAGATTAGGCATTATTAATCAATAGGAGGTAAAGTTAACGAGAG GCTTTTG
GCAGTAGAGTAGGTAGAGATTAGGCATGACCGTTTTTTATGGGAGGGATTTTGCTTTTTTA ACAACTTTCAA
GCAGTAGAGTAGGTAGAGATTAGGCAGGTGCGGGCCTCTTCGTGATTGCTGGGTAATTTA ACATAA
GCAGTAGAGTAGGTAGAGATTAGGCAAAGCCCCATTTTCAGGGATACCGTCGCCATTCAG GCTG
**H2 design oligo sequences (5′-3′)**
GCAGTAGAGTAGGTAGAGATTAGGCATTTTTTTTTTTTTTTTTTTTAGAGACGTTTTTAGAA ACGACTTGTAGAATTTTTGTCAGCGTGGTG
GCAGTAGAGTAGGTAGAGATTAGGCATTTTTTTTTTTTTTTTTTTTCGAAAGGTTTTTGGAT GTCAGTACCTTTTTTTTTCATCGGGAGAAA
GCAGTAGAGTAGGTAGAGATTAGGCATTTTTTTTTTTTTTTTTTTTCGTCGGATTCTCCGTG AGAATAGACAGAGGGGCCCTCGTT
GCAGTAGAGTAGGTAGAGATTAGGCATTTTTTTTTTTTTTTTTTTTCTCCGTGGTGAAGGGA CAACCGCATCACCCAACGTGGACT
GCAGTAGAGTAGGTAGAGATTAGGCATTTTTTTTTTTTTTTTTTTTATAAGTATATCAATAT AATCCCTATGTTTACCAGTCCC
GCAGTAGAGTAGGTAGAGATTAGGCATTTTTTTTTTTTTTTTTTTTTTATTAATCAATAGGA GGTAAAGTTAACGAGAGGCTTTTG
GCAGTAGAGTAGGTAGAGATTAGGCATTTTTTTTTTTTTTTTTTTTTGACCGTTTTTTATGG GAGGGATTTTGCTTTTTTAACAACTTTCAA
GCAGTAGAGTAGGTAGAGATTAGGCATTTTTTTTTTTTTTTTTTTTGGTGCGGGCCTCTTCG TGATTGCTGGGTAATTTAACATAA
GCAGTAGAGTAGGTAGAGATTAGGCATTTTTTTTTTTTTTTTTTTTAAGCCCCATTTTCAGG GATACCGTCGCCATTCAGGCTG
**H3 design oligo sequences (5′-3′)**
GCAGTAGAGTAGGTAGAGATTAGGCATTGCAGTAGAGTAGGTAGAGATTAGGCAAGAGA CGTTTTTAGAAACGACTTGTAGAATTTTTGTCAGCGTGGTG
GCAGTAGAGTAGGTAGAGATTAGGCATTGCAGTAGAGTAGGTAGAGATTAGGCACGAAA GGTTTTTGGATGTCAGTACCTTTTTTTTTCATCGGGAGAAA
GCAGTAGAGTAGGTAGAGATTAGGCATTGCAGTAGAGTAGGTAGAGATTAGGCACGTCG GATTCTCCGTGAGAATAGACAGAGGGGCCCTCGTT
GCAGTAGAGTAGGTAGAGATTAGGCATTGCAGTAGAGTAGGTAGAGATTAGGCACTCCGT GGTGAAGGGACAACCGCATCACCCAACGTGGACT
GCAGTAGAGTAGGTAGAGATTAGGCATTGCAGTAGAGTAGGTAGAGATTAGGCAATAAG TATATCAATATAATCCCTATGTTTACCAGTCCC
GCAGTAGAGTAGGTAGAGATTAGGCATTGCAGTAGAGTAGGTAGAGATTAGGCATTATTA ATCAATAGGAGGTAAAGTTAACGAGAGGCTTTTG
GCAGTAGAGTAGGTAGAGATTAGGCATTGCAGTAGAGTAGGTAGAGATTAGGCATGACC GTTTTTTATGGGAGGGATTTTGCTTTTTTAACAACTTTCAA
GCAGTAGAGTAGGTAGAGATTAGGCATTGCAGTAGAGTAGGTAGAGATTAGGCAGGTGC GGGCCTCTTCGTGATTGCTGGGTAATTTAACATAA
GCAGTAGAGTAGGTAGAGATTAGGCATTGCAGTAGAGTAGGTAGAGATTAGGCAAAGCC CCATTTTCAGGGATACCGTCGCCATTCAGGCTG

Table S6. Staple sequences used for the T1 ring triangle folding.

Core structure oligo sequences (5′-3′)
GCAAAGACAAGGTGGCAACATATATGGTGATGGTGGTTCCGAATAGCC
TTCCATTATAAATTGGGTCAGGACACAGGTAGAGGTCTTTA
ATGCGCCGCTACAGGGAACGTGCTGGAGGCCG
TCTCACGGTACATCGACATGGAGAGGGTAGCT
GGGATAGCGGAACGCCTCTGGAGCAAACAAGAAAAGGCCG
AGAGAGAAATTGAGTTAAGCCCAAGAGATAAC
TTTTTCAAAATTTAAGACGCTAATTTTC
CCTGACTATTATAGTCAGCTTCAATGAATTACCAACAGTT
CGGAACAAGATTTACACCAGAAACAAAGAAAACGAAAGCGCG
CGGTGCCCTCGTTAACGGCATCACCACGGGACAGCGGTTTGTTA
GCGGTGCTGTCACTCGGGCGCCCAGCATCCGCCAG
AACAACATGTTCAGCTCGAGCATGTTTTTAACCAAATATCCTAAAGCA
TCTGACCTAATATATTTGCAAATCCAATCGC
GAAATACAAATGCTTTAAAAGATTAAGAGGCGCGAGAAAAC
ATCAGTTGACATTATTGTTGGGAAGAAAAATGGGAGTT
GGAATACCACATTTACGAGCCGGAACCTGTCGTGCCAAAACGAACTAA
GCGCGCCTGTGCAAATAAGAGAATAACAATAGATAAGACCTGCAGCCA
AAATAAGGCGTTATATATTAATTGAGAAGAGAACCTACCACAAAGAA
GCGGAATCGAGAATGACCATAAATCAATTTTTAATCAAAGATTC
CAATACTTTGATAAGAGCGAACCATTTTCTGTCAGCGGACGAATAAT
TTACGGGAATCAACGTACGAGTAGAACGGGTAAAAGGCCG
TGAAATTGGCCTGGGGTGCCTAATGAGTTTTTGAGCAGACGATCCAGCGCAG
TCCTGTGCCGCCTGGGTATTGGGGGGACGACGCCAGCTT
CATGTAATAAGGTAAAGTAATTCTGTCTTTTTAGACTTTCATCT
ACGCCAACCGCACTCTAGAAACCTGAAAAATAAACCCTC
AGAATAACTTTTTTCAAGAAAGCTTTGATTGCTATTTATTTA
ACGTTATACAACTAAGAACCCATAGACGTTAGCCCTCATCCTTTAAT
ACAAACAATTCGACAACTCGTATCTGGCCAAAATTATTTGCACGAAG
ATACATTTGAGGATTTAGAAGTATGAAAGCGT
TTAATGCGCGAACTGACTAAAATAAATTCATCCTGAACCT
GGCGAAAGGGGGATGTGCTGCAAGAACCAATA
GTCTGGCCTTTTTTTCTGTAGCCAAAGGTTTTTTATCAGGTCATTG
GCTTTCATCAACATTAGCGCAACTGAATTTGTGGAAGATC
GTTGGGTAACGCCAGGGTTTTCCTGATAATCATCAAACTTAAATCTG
ACCGCCACCCTTTTTCAGAACCGCACGGTTTTTGTCAGTGCCTTGA
CACCCTCAGAGCCACCAAATCTCCAGCAACGGACAACTTT
ATAGCAAGCCCAATAGAGGAATTGGTGAGAATAGAAAGGA
TTTTGAATGGTTTTTTATTAGTCTAGTATTTTTAAGAACTCAAACT
AAAACGACGTTGGCAAATCAACAGAATCAATATCTGGTCA
CTTGCTTTCGAGGTGAATTTCTTATACTCAGG
AATTTTTTCACGTTGAACCCTCATTTTGCTAAATGATACAAACGCCTG
CCTGAGAGATCAAAAATAATTCGCCGCCAGCTCGCCATGTTT
TACGGTGTATTTTATCCATTACCAGGCGCTAGGGCGCTGCGCGCTTA
GCAAAATCTTAGCTATATTTATAACCACGG
CGAGATATCCACTATTATTTTCGTCTCTTTTTTCGCGCAA
AAAACGCTCTATAAAACAGAAATACCTTAGAATGAATTAC

TACCGCCAAGAACCCTTCTGACCTTAGACTTTACCACCAGAAGGAGCG
ATCGGCCTGTGGCACAGACAATATAATAGATACCTGATTATC
GCGCATTAGATCGCGCAGAGGCGTAGATACCAAG
TTTGCACCCAGCTACAGAGGTTTTAATTACATAAAAATTACTAGAAAA
CATCAATATTTTTGATATTCAACCGTTCTCGTGAGAGATCTACA
TAAAGGTGAAAATCCGCGACCTGCATTGATAAATCCGCCTCC
CCAAAAGAACAACGCGGTCCGTTAAGGATTGCCGTGTACCA
GTCAAAGGCAGTTTGGGTAGAACGGTAGGGGGTTTCTGCC
GACGATTGTTTTTCCTTGATATTCACAACTGGTAATAAGTTTTA
AGTTGATACCATTAGATACTCCATGTTATTTTTTTAGTTGACGGA
GTAACAGTTACCGCCACCCTCAGATTTATCAGGACAGCATCG
AATCAGTGTTTTTGGCCACCGAGTAAAAAAACATCACTTGCCTG
TTAACGTCTTTTTAAAATGAAGGGAGCCCCCTTTTTATTTAGAGCTTGGTTTTTAT
CTCAGAGCATAAAGCTTAAGAAAAGTAAGCAGATAGCCG
TAATTTGCCAGTTACAAAATAAAAAGGAGCGTTAGATTCGCCTGATT
GTCATAGCTGTGTCGACCCCAGCGTGGCTGACTTACCCAATAGCGTC
TTCATCGGCATTTTCGAGCGCCAAAGACAAAAGGGCGAC
GAGAAAGGACAGGAACGGTACGCAACAATATTTTTCAGG
GTAATTGAAAGTTTTTCAAGCAAGACCAAGTA
ACAAATAACTCTGAATTTATTTTTCGTTCCAGTTCAAGGTTGAG
ATCCTATCAGGGCGATGGCGGGTAAAGTTAAACCGGACTTAACAAGAGGGGTTGAG
TTCCGGCAGTAAAAAAAAATGCCAATTACGGCTAGCTGTT
TACTCATTTGGGGCGCGACGGAGATTTGTATTGACCAACTTAGTTTGTCCCAATT
TTATCACCGTCACCGTTTTTCTTGAGCCATTTGGAATTATTCAT
GAATTAGAAGTAGCGATAAAGCCATAGCAGCAAGTTTCGTCACAGACAGTAAATGA
AATCACCAGTAGCTCAACATGGAACGAGGCGCAG
GGTAAATAGCGTCTTTCCGTAATCGCCAGCAAACCATCGATTTGCGGATGCTCCTT
AAAATCCCTGGCATGATTAAGACTTTTTCCTTATTACGCAGTAACGGAATAC
ATTCTACTAAAATACACGAAAATCCTGTTCAGCCTCCGGCCA
TACAAGCTGATGAACGTCAGTGAAACACAACTAATCGCCAAAAGGAA
AGCGCAGTATCCTCATCAGAATCAAGTTTGCCCGATTGAGGGAGGGAA
ATAACGGCAATTTCATTTCCTTGAACCTCCGACCGTGTGAT
GTAACAGTCAGTACATATCGTCGCTTTAACA
AATATACACGGGAGAATTAACTGTTTTTACACCCTGAACAAAAAACAGGGAA
AGCAATACATGTGAGAAGTACGGGGAAAGCCGGCGAAACGATTTTTTGT
AATAAATAAATATAATCCTGTTTTTTTGTTTGGATTATATCATATT
ATTAACACCGCTTCTGCTCATTTGCAGCGGGGGTCTGGTC
ATTTTGATGAGAGATAGACTTTTTTTCTCCGTGGTGAAACGTACAG
GAACAAAACATCCAATAAATCTGATATTTTTATTAATGCCAAAGAGACAGT
ATTCAACTTTAGCGTCAGTTTTTCTGTAGCGCGTTGCAGGTCA
GGCTTTTGCTACAGAGGCTTTTTTTGAGGACTAAAGACCAGCGAAA
GTAACGATCCAGTCACACGACCGTAACACTGAGCGGTCCCAATGAA
GCGCTAATGCGCCCAATAGCACCTGAGCAAAAGA
TCACCTTGCCGAACGAACCACCAGAGGACGCAAATTAACCGTTG
CCACGCTGAGAGCCAGGTGAGGCGGTATTAACCGTTTTTGTAGGGCT
ACACCGGAATCATAGAAGTTTGCTGAATCCC
ATGGGTAAGTGGTGCCATCTTTTTCACGCAACCAGCCGGCAGC
AACCGTGCATCTGCCATGGGATAAGCTGATTGCAAGCGG
CACTACGGGGTTGCCCTGATAGCTGCATTAATGAATCGGCCTAACCGA

AACAAAGATTAGCAAAATTTTTTAAGCAATAAAGCCAAATCAC
AGATAAGGCACCAACCTCTGCTCATGTGTACAGAGCAACACTATCATGAGG
GAGGAAGTCACTAAAACACAAGCGTCATACAT
TTTTTCATAGGTTTAGGCCCGTATAAACAGTTTTGACAGGTCTTTGAC
TATGGGATTTTCAGGGCAAACTACGGAGTGTA
CCACGCAGTGCCGGAAACCAGGCTCCGGCACCGCTTCTG
GATACCGATAGTTGCGCCGACAAAGGCTGAGTAATGC
ATAATGGATTTAACGTCAGTTCTTTGATTAGT
ACTTCTGAAAGAATACTGCTGGTAATATCCAGCAGAATCC
AGATGATGGCTCTTTAGGAGCACTAACAACTATTTTTTAGATTAGAGCCGTC
ACCAGTCCCGGTTGGGAAGGGCGATCGGTGCGTTTTTGCCTCTTCGCTATTA
GAACGAGGGTAAAAAAAAAGGCTCCAAAAGGAGTTTTTCTTTAATTGTATCGG
GAGTAACATTATCATTAAGACAAATTAGATTAATGGTTT
AGGGTTAGTCAATAGTAATGCTGATTAGTTAAGACGACAAT
CTCCGGCTTAGGTTTTTTGGGTTATATAACGAATTATC
ATCCCATCATCGGCTGACCGACAATTAGGCAGAGGCATT
TCAGCAAACCTGCATCTAACTCACATTTTTTAATTGCGTTGCGCTC
TGGTGTGTGGGTCACTGTTGCCCTGTTTTTGGCTGGTA
TTGCTCGTCATATTTTTACATCCCTTACACTCGGCGAA
CGGTTTGCAGGTTTCTGCACTCCAGACAGTATCGGCCTCATCTCCGTGG
AACGCGCGTGGTTTTTAGTGTAAATTATCCGCTCACAAT
TTATACCAGCTTGAGATGGTTTAATTTTTTTCAACTTT
CTTATGCGATTTTTTTTAAGAACTGGCTCAACCCTCAG
ATCGCGTTAGCAAACTCAGAAAACGTCATAAATATTCAT
TATATGTAGAATTTATCAAAATCATTTTTTGGTCTGAG
AGACTACCGAGTGAATAACTTTTTTTGCTTCTGTAAAAATCAA
AATCAATACTAATTTAAATGCAGAACGCGCCTGTTTATCATAAAGT
TCTTTCCTTATCGCTCAACAGTGGCCAAGCTACGTTGT
TTCGCACTGTTTCCTGAACAAGAAAAATAATGGCCAGT
TTTGAAAGAGTTAACCCTCGTTTACCAGACGACGAT
CGCCAGGGGGGAGAGGACTGCCCGCTTTCCAGTCGGGAAAGCATAA
CTTTTCACCAGTGGCCGCATCGTTATATTCGACCATCGC
AATCATTGATCTTGACAAGTTTTTACCGGATATTCACTTCATC
AGACCGGATTAATTCGAGAAGCAAAGCGGATTGCATCAAAACAGTT
CCAACAGGTCAGATATTTTGTCGAAAAGTTTTTGCCCGA
GCGATAGCGAAAAGCCCGAAAGACTTCAAATTAATTTT
GGCATTCCAAGAACGGGTCAGTACCATCACCCAAATC
TCATTGCAAGTCTCTGTGGTGCTGCGGCCAGAATGCCAT
GGAGGTGTGGTTGCGGACGCAGAAAACGGATA
AACGATTAGAGAGTAAGTTAGCAACGTCAGAGCGGGAG
TATATGTATCGAGAATGGGGTCGCAGAAGATAAAACAGAGCAGCAAA
ACCGTCGCCCTGAGATAGCATTACGGCGGATTGACCGTAAGTTTGAG
AAGAGTAAGGTCATTGAATGGAATAGCATTCCACCAGTA
TGGCTTAGGGTAATAGCCAAAATAGCGAGAGTTCATTCACTAAAG
GAGCACATCCTCATAACGACCGCAAGAGCCGCACCAGTTGGG
GCTTTTGCTTAGAATATAATGCTGTAGCACCTGAATC
ACCGAACCATCGCCTTCGCAAATAGAAAATTCATATGGTTTACC
ACGGTCAATCATAAGGGAGCATAGGCATTATACCAAGAGGCA
ACAAAATTGAAGCCTTACCTCCCGACTTGCGGCTGGAAGT

AGATGATGAAACAAACATAATGGAAAACCTTTTATGCGTAGA
ATGCGTTATACAAATAGAACGCTAGAAGGCTTATCCG
AGCACGCGTGCCTTTTTGTTCATTCGTAATTTTTTATGGTGGCGG
AAAATAAAATGTTTTTTTAGACTAGGCATAGTAAGACCAGGC
GCCGTTTAGCAGCAGAACGTGCGATGCTAACGTGGAGCTATC
AGCCTGTTTAGTTTTTTTCATTAATTGAGATTTTTTCGCCAAATA
CTCAACAATTTTCATCGTAGCGCTACGTGA
TCACGGTCATACCTAAAGCCAACGTTCGAGCC
GGGTACCTCCACGCTGGTTACAGAAAAGGTTTGGTGT
GAGCTCGATTCGCGTCCGTGAGCATCAAAAGAAATCG
ATCCCCTCCACACATAAGAGACGGGCAACGGTCACGGGCATCA
CAGAGGGAGCTTAATTGCTACAAGGCCGGA
TTTTTAAACAGGAAGATTAACTAGCATGTCAATCATATTTTT
TTTTTCAATGCCTGAGTAATGTGTCTTTAGTGATGAACCATGGTGCTG
TTTTTAGCACTAAATCGGAACCCTAAAAATAGCAGCCTTTAC
TTTTTCATCGCCATTAGAGTCTGTCCATCTGCCGTAATTTTT
TTGAAAGGAATTGAGGAAGGTTATTAGCCCTAAAATTTTT
TTTTTTGTACCCCGGTCAGTCACGTTCAGAGGTGGAGCCGGATGCCGGCAATCCGC
GCGAAAAACCGTAGATAATAAGAGCAAGAAACAATGAATATTTTAAATGTTTTT
TTTTTTAAAGGGTGAGGAATCGATCGGTTGTGAAAAAGAGTATGAGCC
GATTCAAATACTTTTGAAATATTTAAATTGTAAACGTTTTTT
GAACGGTAAATCAGCTAAATTCGCATTAAATTAGAAAAGCCCCAATTTTT
TTTTTTAATATTTTGTTACATTTTTTGCGATTAAACCTCACCGGAAACAA
TTTTTCGCAAGGATAAAACCCTCATAATAGCAATACTCCAAC
ATCGTAAGTATAAGCCGGGAGAAGCCTTTATTTCAATTTTT
AGGCGGCAGGTAAAAAATAGAAATTTTACATTATGACCCTGTTTTT
TTTTTGCCACCACCCTCAGAGCCGCCTCTGAAACATGTTTTT
AAAGCGCCATTCGCCATTCAGGCTAATGTGAGCGATTTTT
TTTTTGTAACAACCCGTAGCATACAGGTTTTT
TTTTTCAAGGCAAAGATTACCAGAAGGAAACCGAGGAAACAAAGAAAC
TTTTTAAAGTATTAAGTGACAACAGTCGCTGAGGCTTGCACTACGTTAACGAGAAA
CACCACCCTCAGAGAGAGCCACCACCGGAACCCCTTATTAGCGTTTTT
AGAGCCGCCATAATCATTGCTCAGTACCAGGCGGATATTTTT
TTTTTAGGATTAGGATTACCTATTATACCAGAACAAACAAAG
TTTTTACCCCGCCACCCACAATCAATGGTCAATAACCTGTGAGTAGAT
CTGCCTATAATAGGTGGGGTTGATATAAGTATACTCCTCAAGAGATTTTT
AAATACGAGCCCGGTTCGGAAGCGGGGTTAAATCACCGGATTTTT
TTTTTTTTGCCATCTTTTCGCCAGCAAATGCCCCAAAGAATA
TTTTTAGTGCCGTCGAGATATCACCGAACAGCTTCTTTTGCGGGATCGTC
TTGCGGAATATCAACAGAGATGCCATTGTTTTG**T**
AAGAAATCATCGGGGATTTTAGAAGGGAAATGGT**T**
CTAAACATTCCTCGTTAGAATCAACGCTGCGCG**T**
TCTTTCCGTACCAGTAATAAAATACATTGG
CTCAATCGTCTGAAATTACCTACACAACAGGAATTAAAGGAGAAACA
**T**ATTCACATGGAAAGGATTATTGGGACATTTAAATCCT
**T**CCACACCCGCGCAAGTGTCCAGAGCCTTACCAAC
**T**GACGAGCACGTATCGCGTACTGAAAGCGACAGCCATA
**H1 design oligo sequences (5′-3′)**

GCAGTAGAGTAGGTAGAGATTAGGCACCACAAGATTAAGCAAATCAGATAGAGGCGTTTTTTTTAGCGAAAATCAA
GCAGTAGAGTAGGTAGAGATTAGGCATAATATGTTAGCAAACGTAGAATAGTAGGAGTTGCAGCCCTTCA
GCAGTAGAGTAGGTAGAGATTAGGCAAATAAGTTTATTTTGTCTCAGAACCGCCACCCTCAGATTTTT
GCAGTAGAGTAGGTAGAGATTAGGCATCCCAATCCAAATAAGAACGTGGCTTACAAAA
GCAGTAGAGTAGGTAGAGATTAGGCACTGCGAACCCTTATAACTCCTCACAGGTGCCCCAGCAGG
GCAGTAGAGTAGGTAGAGATTAGGCATTACCGAAGCCCTTTTAAATCGGT
GCAGTAGAGTAGGTAGAGATTAGGCAGCTAACGACCGTTTTAAATATGCACATATAAC
GCAGTAGAGTAGGTAGAGATTAGGCAGATAATTATTCATTTTTTTCAATAACATAAGTCAGAGG
GCAGTAGAGTAGGTAGAGATTAGGCATGTTGTTCGTATTCTATCTTACCATCAGGAATCATTACC
**H2 design oligo sequences (5′-3′)**
GCAGTAGAGTAGGTAGAGATTAGGCATTTTTTTTTTTTTTTTTTTTCCACAAGATTAAGCAAATCAGATAGAGGCGTTTTTTTTAGCGAAAATCAA
GCAGTAGAGTAGGTAGAGATTAGGCATTTTTTTTTTTTTTTTTTTTTAATATGTTAGCAAACGTAGAATAGTAGGAGTTGCAGCCCTTCA
GCAGTAGAGTAGGTAGAGATTAGGCATTTTTTTTTTTTTTTTTTTTAATAAGTTTATTTTGTCTCAGAACCGCCACCCTCAGATTTTT
GCAGTAGAGTAGGTAGAGATTAGGCATTTTTTTTTTTTTTTTTTTTTCCCAATCCAAATAAGAACGTGGCTTACAAAA
GCAGTAGAGTAGGTAGAGATTAGGCATTTTTTTTTTTTTTTTTTTTCTGCGAACCCTTATAACTCCTCACAGGTGCCCCAGCAGG
GCAGTAGAGTAGGTAGAGATTAGGCATTTTTTTTTTTTTTTTTTTTTTACCGAAGCCCTTTTAAATCGGT
GCAGTAGAGTAGGTAGAGATTAGGCATTTTTTTTTTTTTTTTTTTTGCTAACGACCGTTTTAAATATGCACATATAAC
GCAGTAGAGTAGGTAGAGATTAGGCATTTTTTTTTTTTTTTTTTTTGATAATTATTCATTTTTTTCAATAACATAAGTCAGAGG
GCAGTAGAGTAGGTAGAGATTAGGCATTTTTTTTTTTTTTTTTTTTTGTTGTTCGTATTCTATCTTACCATCAGGAATCATTACC
**H3 design oligo sequences (5′-3′)**
GCAGTAGAGTAGGTAGAGATTAGGCATTGCAGTAGAGTAGGTAGAGATTAGGCACCACAAGATTAAGCAAATCAGATAGAGGCGTTTTTTTTAGCGAAAATCAA
GCAGTAGAGTAGGTAGAGATTAGGCATTGCAGTAGAGTAGGTAGAGATTAGGCATAATATGTTAGCAAACGTAGAATAGTAGGAGTTGCAGCCCTTCA
GCAGTAGAGTAGGTAGAGATTAGGCATTGCAGTAGAGTAGGTAGAGATTAGGCATAATAAGTTTATTTTGTCTCAGAACCGCCACCCTCAGATTTTT
GCAGTAGAGTAGGTAGAGATTAGGCATTGCAGTAGAGTAGGTAGAGATTAGGCATCCCAATCCAAATAAGAACGTGGCTTACAAAA
GCAGTAGAGTAGGTAGAGATTAGGCATTGCAGTAGAGTAGGTAGAGATTAGGCACTGCGAACCCTTATAACTCCTCACAGGTGCCCCAGCAGG
GCAGTAGAGTAGGTAGAGATTAGGCATTGCAGTAGAGTAGGTAGAGATTAGGCATTACCGAAGCCCTTTTAAATCGGT
GCAGTAGAGTAGGTAGAGATTAGGCATTGCAGTAGAGTAGGTAGAGATTAGGCAGCTAACGACCGTTTTAAATATGCACATATAAC

GCAGTAGAGTAGGTAGAGATTAGGCATTGCAGTAGAGTAGGTAGAGATTAGGCAGATAA TTATTCATTTTTTTCAATAACATAAGTCAGAGG
GCAGTAGAGTAGGTAGAGATTAGGCATTGCAGTAGAGTAGGTAGAGATTAGGCATGTTGT TCGTATTCTATCTTACCATCAGGAATCATTACC

Table S7. Staple sequences used for the T3 triangle 1 folding.

**Core structure oligo sequences (5′-3′)**
TATCAGGGACGGGGAAAGCACTAAAGCGGGAGTAGACAGG
AGAACGGGATCCGGTAAGCAGCCTTAACGTCAGTGAGAGA
AAAGTGTCTGCCCGCTTTCCAGCGGTGCCGGGGGTTTCTGCCAGC
TATTAGCTGGTAATATAATACCTATCCTCGTTCAGGGCGC
GGAAACAATCGGCGAAGATAGCTCAAGAAACACGGAATTT
AACAAAATTAATTACATAAGTATACCACCCTCAGACTCCT
GCAGAGGCATAACGGATGAGTTTCTCCACAGACCTTGAGCAAATAA
CCACCCTCTAGGTGTATCACCGTAAAACAAACTTACCTGA
TAACAGTGCCCGTATAGGTCAGTGCAGCCCTCATAGGAGCGTAGATT
CATACTGGTAATAAGTTTTAACGGAACAGTTACATGTACGGATAGC
TTCTAAGAGAGAAACAGAATTATTCAGAGCCAGCCCGGAA
AATAATATCGTTTTAGCGAACCTCACGATTTTTCTTACCAACG
ATCGTCGCGATGCAAATCCTTTTTATCGCAAGACAAAGATGATG
AGCGGTGCTCGGGAAAGATCCCCGACACAACAGCAGCAAGCGG
AATAAGAACCGACTTGCGGGAGGTAATAGATAAGTCCTGA
CAAGTACCGCACTCATTAGGAATCCTCACATTGTGTCACT
TTCGCCTGTTCAGGTTTTACAGAGCGGCAATAATAAGAGCTCACGGAATTCTCCGT
GTAGAAGAACTCAAATCTTTGATCAAATTAACCGTTGTA
GAATGCGGCGGGCCGAGGCGGATGTGTTTTTCTTTTCTGAAACATGA
CCGCGCTTAATGCGCCGCTAAGAATCAGATCGGAACATCCGCTC
CCAGAACAAACGGTACTGAGGCCAGCTCGAATGTCATACCCCCTGCAT
CCAAGCTTTCAGAGGTGGAGCCGCCATTTTTGGGAACGGATA
CAAGTTACTGCGGCCAGCGCGCCTGTGCACTCTTTAACAAAGCATGTA
GCAATAGCTATCTTACACCTCACCCGGAAATTTTTTATTCATTAAA
AAGTATTAATTAGCGGTCGAGAGGGTTGATATGTGGTGC
CAGACGATCCAGCGCAAATTGCGTATGGTCATAGCTTACAAGCCCAA
GTACATAAATCAATATATGTGAGTGATTTTTTAACCTTG
AATGAGTGGAAGTTTCCTGTGTGCCTGAGAAACGTGCTT
CTTTCAACAATTTACCGTTTTTTTCAGTAAGCGTCATACATGG
AAGTGCCGGGTTTGCTCAGTACCGCAATACTCTATCGGCATAATCAG
CAGTTGAGCCTGTCGTAGAGAGTTTACGAGCCCCGTGGTGGTTCCGAA
AAAATCGCGCAAGCAAATCAGTAAATTACCGCGCCCAATAGAAACCAA
GATGAATATGTAGCATGTCACCAGTACAAACTGCCACCCTCATTTCAA
AGAGCCGCAAGAGAAGGATTAGGAGAGGCTGATTTTCAGCGTAACAC
AAGGTAAATATTGTTTACCAGTCCATGAAATACGTCAAAAATGAAAAT
GAGATAAAGAAATTGCACGTATATAGGAACCTCGGAACC
TTTAGCACAACGCCTACAGTAACAGTAGAATTGTTTAA
ATTCACAAACCACCAGAACCATTTTTCACCAGAGCCGC
TTTGATTTTTAGCCTTAAATCATTATTTATTTTTCCCAATCCA
CCGATTTACTTCTGAATTTCTGCTCATTTGCCGCCAGC
TAACGAGCGTTAGATTTTGCACCTTTTTAGCTACAGAACGCGA
CGAAGCCCTTTTTATTTTTGAAAAGTAAGCAGATAGCCGA
GTAACGATAGCAAAATTATTTGCGCATTAGAAGAATAACGATAACCC
AGCGGATCAGGGAAGCACGTAAAACAGAACCCGTACTATGTAATCCTGTCAGATGA
ATCAAGAAGAATTACCTTTTTAATGGAAACAACAAGAAAGCAAAAGA
CCCTCAGAACCGTCAGACTTTTTTTAGCGCG

GATACAGGAGTGTTTTGACGCTCACCTGATTAATTGTTTGGATCTGGCA
ATAATGGAAGGGTTACATCAATAGTTGCTTT
GGGATTTTGTTAGTAAATGAATTGAACCTACCATATACTAAAGAGACGCAGAAAC
TTTGGGGTCGAGGTGGGAAGCATACAATTCC
CTTCTGTAACAGAACGCGCCTGTTTTTTTATCAAC
AGGGCGAAAAACCGTCAGTTGGGCAAAGGAGC
GTCACCTTTTTACTTGAGACGTACAGCGCCATGA
ATCCCGTAGAAGATTTTTAAGCGGGTTGTGTACATCGAC
TTAGAAGTGCCATTGGTGAGGCGGTGCCTG
AGGAATACCGAACGAACCACCAGCCCACGCTGCACCTTGCTGAACCT
TCATATTATCGTCTGATTTTTATGGATTACAACAGGAAATTTTTACGCTCATGGA
AGAACCCTTTACATTGGCAGATACCAGAAGATCATTTTGCGGAACAATGCTGAT
CATCGCCATTAAACAAACTGATAGTGGCTATTAGTCTTTA
CAGCAGGCTTTTTAAAATCCTGTTTGATAAAGCCGGCGAACGTGG
TGGTCAGCAGCAACCGAGCACATCAATTTTAAAAGTTAACCACCACACCC
ACGTTATTCTCATAACTGCCGTTCAGGGTAAAGTTAAACGAGTTTGAG
AATCATACTGACCATTTTCTGCGAGCTTGCCC
GTTCAGCAAATCGTTAACTTTTTGCATCAGATGCCGGGTTACCTGCAGCC
GGTACCGACCGAGTAAAAGAGCAGTTGTTTTTAAGGAATTGA
TCCTCACTGTTCTTTTTTTGCGTCCGTGAGCCGGGTCACTGT
CCAACGGCAGCACCCCAGCCCGAGGAGTCCACTATTAAAG
GCTCATGCAAATAGATTTAAAGCATAGAGCCAGCAGCAAATGGGCGCGA
CTTATAAATCGCTGATTGCCCTTTTTTTACCGCCTGGCCCTG
TTTTAATGACAGTATCGGCCTCAGGAAGATAATATTC
CTAACGGAACAACATTATTACATTTCAACTTTAATCCAGCGATT
GCCAGCTGCATTAATGATTTTTTCGGCCAACGCGCGG
ATATTATTTTTCGCCAATTAGACTGGAAGGTTAATCCGCCTGCCCTGC
CCGATTATTTTTAGGGATTTCTAAACTTTTTGGAGGTTACAAAC
GAACTGACCAACTTTGCCGAATAACCTGTTTAGGTGGCATC
AGACAATATCTGGCCATAAGAATACAATAGAAACAACT
GCTATATTTTCATTTGCAATCATAAACGTAACAAAGCT
AGGTCTTTCAAAAGGATGTTTTAAAACAGTTGATTCCCAA
AAAAAAGCCGCACAGGCGGCCTTTAGTGATGACGGCAAAC
TGTTACTTGTCATTGCAGGCGCTTCAACCAGCTTGAGGATAACTCGTA
CTTTTGATGCTCAACACAGTTCAGTGACGAGAAACACCAGATTCATTA
AATCAATATCTGGTCAGTTGTTTTTCAAATACGCGTGC
AAAACGAGAATACGTCAGCGTGGTTAATTGCAGAGCCGT
AGATACATCCCAAATCAGGGAACCTTTGTATCATCGCCTGATAAATTG
TCAGCGGGAGCCGGAATCCGCGACCTGCTCCAAGGTTTCT
GAGGAAAGCCAGCTTTCCGCAATACACTATCATAACCCTCGT
GGAACGTGCCGGACTTGTAGAGACATTTTTGCGCGCATCGGATAGGTC
ATGCGCGAGGCAAAGAGTACCAAAATGCTGTAAAGAGGTCCATAAAT
TTGCTCGTGGCTGGTAATGGGTAACAAATATCTATCTTTA
TGTCGAAACGAGGCGCAGACGGTGAAAAATCATTTAGTT
AGTGTAGCGTTTTTTCACGCTGCTTTTTTCGTCTCGAGG
GGGACGACTAACCGTGCATCTGCCGTAGATGGGGATGGCTCACACGAC
CAGATGAAATACCAAGCGCGAAACAAAGTACAACGGAGA
ACCGCCTGCAACAGTGAGAAGATAGGAGCACTTAATACATTTACGGCT
TAAAAAAAACGTGGACTTTTTCCAACGTCAAATCGGCAAAATC

CAATCTGTCCATCACGTAGTAATAACTTTTTCACTTCAG
AAACAGAGTTCTGACCTGAAAGCGACAGAGATTTAAATCCTTTGAGC
AAGAGCAACTGCGGAATCGTCATACGCACTCCGCCCGAAA
GGGCGCGGGGTTTGCGAGTGAGACGGGCAACAAAAAGAAT
TGCTGGTCGGAGGTGTCCATCAGTGAATAAGACGAGTAGTGAATATA
CATAAACATCCCTTACACTGGTGTGGAGAGGCTTGCGGTATGAGCC
AACATAAAAGGGACATTTTTTGAACCCTAAAACGTGGCAC
ACCCTGACTATTATAGTTTTTTAGAAGCCCACATTCATTTTTCTAA
TCAAAAGGATTCGCAGTAAACGCTCAGCAGAATCATAGAAGAGTCA
ATATGCTTTTTACTAAAGTACGGAGAGTACTTTTTTTAATTGCTC
CTCCGGCTAACATAGCGATAGCTTAGATTAAGTTAATTGATTGAAAT
ATTCGCGTCTGGCAAACAGCTTGGCGGGATCGTCCCGTGTGATAAA
GGGCGATCGGTGCGGCGACAATGAACGCCATTCAGCTCAAAGCCTC
TCTTTGACCCCATTGTGAATTTTTTACCTTATGCGATAATAAAACGA
ACCGAACCTTAATATTTTGCCTGAAGATCTACAAAGGCTACGCCACCC
TCCTCATTAAAGCCCAGACGATCGGTCATAATCAAAATACCTAAAT
TTCTAGCTGAAGTAGTAGTTTTTATTAACATCCAATAGCTGAAAA
GTGAGAAAGGCCGTTGAGGCAGGTAGAATGGAAGCACCGTTACCATTA
ATAATTACCTTTCCAGAGCCTTTTGACATTCAACCGATTGAGGGAGG
CAAAATAAACAGCCATAAGATTAGTTGCTAAAACATGTTCAGCTAATG
ACGACGACTTAATTTCCCGGAATCTTTTCATCATCAAGTTTGCC
CGCCAGCATCAGAACCCCGCCTCCCTCAGAGCTTTTTAACTTAATGGT
AATCGGTTATTAGCAAAATTTTTTAAGCAATATTTTTTAAACAGGAAG
AAGAGAATAGCAAATATTCAACCGATGTGTAGGTAAAGATTGCAATGC
CAGTAATTATGACCCTGTAATTTTTTCTTTTGCGGGAGAGCATAAAGCT
GAAACCGAAAAAGGGCTGTCACAAGCGACAGAGGCATTTTTGGCCTTGA
AAAACTTTTTCAAATATAAATGCTTATTAATTAATTTCCCTTAGAAT
GCTACAGAGGCTTTGACCGATATAAAGTTTTTTTTATTCCATAT
AACTGTCATGCCATTCGCCATTCAGGGGGGGATGTGCTGCAAACGCCAGC
TCAGTTGTTTTTAGATTTCTACGTTTTTAAGAACTGGCTCACGAAAGAG
CGGTGTTTTTACAGACCAGGGCAAAAGAATACACTAAAACACTC
AAAACCAAAAGAAAAATAGGAATACTGGCTGACCTTCATTTTTTAAGAGTAATC
TCTGGTGCCGGAAACCATAATAGTAAAATGTTTCGACGATA
GCCAGTTAACAAAGTTACCAGAAGGGCCAGTGGGTGAATT
ACAAACGGCGGATTTTTTTACCGTAATGGTCGAGCTT
CGCATAGGGGACTAAAGCATGTCAATCATTTTTATGTACC
CTGAGGCTTGCAGGGAAACGAGGGATTGTATACGATGAAC
ATTCTACTAATTAAGGTAATCGTAAAACTAGACTTTTT
GTTAAAGGTTGTTAAACAAAAATACTGAGTAAGAAGCC
TTGCTTTCGAGGGTTGCAAGGCCCTAAAGGACGGAGTGA
CACCCTTCCTGTAGCGCTTCGGTTTATCAGC
TTCGCAAATGGTCGTTGATATTTTTTCAGAAAAGCCCCAAAA
GAGTCTGGAGCAAACTTTATCAACGAAAGACAGCATCGG
AATAAACAAAGCCTGTTTAGTATCTTAAATTT
TACCAGCGCCAAAAAACAGTCACGACGTTGTAAAACGAC
GTTATATACACGCCACCACCGGAAGCCACCCTCAGAGCC
ATTAATGCCGGAGAGGGTAGCTAATATGATATTTAAATT
AGCACCATAATCAGTATCAATAGATAAATAAGTATACAA
CAGCTTTCATCAAGGTCAGGATTACCAATAGGACAACAAC

TTGCCATCAGGGAGACAGTCAAATCACCATCATTTTTGAG
TTAAATTTCCGCTTTTATACCGATAGTTGCGCGCCTCTTC
TCTAAAGTTTTGTTTTGGGAATTAGATTTTTCCAGCAAAA
TACCAGAAGACTTTTTTGGATAGCGTCGCACCGC
ATTAGCGTTCGATAGCAAGAGAACCCTCATATATTTTAAA
ATAGTGAAGTAAAATACGTAATGCAGTTTCCATTAAACGG
TTGACAGGTTTTCATAGCCCCCTTTAAGGCGTAAATTCAT
ATCTTCTGCACCGGAACCAGACCTCAGAACCGCC
AGGAACAAGGAAACGTGATAAAAATTTTTCGCAGTCTCTG
AGTTTCAGATTGCGAATAATAATTTTTTCACCTCACCAGT
TTTATTTCCCGTGGGAGCGAGTAACAACCCGTGCCTTT
TGGTGTTTTTATTTTCATCGCGAGAACAGGGTGCCTCACCCAAA
TTTTCACGTCGTAATCTGCGCTCAAAAGCCTGAGCAAGCCGGTT
ATTGCTTTAGCATATAGAAGGCTTTATTAAACACAAGAATTGACAAT
AATTTTATCCTCCTTTTACATCGGACGCGAGGCCCATCCTCGGCTGTCGGCT
TTTGTTTCCTTATCATTCCATCAATAATAATTTACGTTTCATTT
TCCTGTTAAGCCGAGAATTAACTGAACACCCATCAGAGAATAAAAAC
TTTGGTAATTGAGCGCTAATTGAACAAATGAACCATAAACTTAA
GCCAGAATAAATTGTTCCTAAAGGTCAAGTTTCACTACGGTCAGAGGTTCT
AAAGAGGATTGACAAGATGGTTTAAGGTAGAGTAAAT
GAGAAAGGATCGCTGGCAGCCTTGGAACAAATAGGGTTGAGTGT
TTTGAGAACCGGATAACGAGTAAAGATTCATGCAGATA
CCACGCTGGTTTGCGTCGGTGGTGCCTTTTCACCTATTGGGCGCCAGT
CAAAGCGAATATCGCGCAAAAATCATTGAATCCCT
TCAGTTCCGGCCAGCAAGAATGAATTCGAC
TTTTTCATCCCACGTCGCACTCATCTAAAAAAACCCTCTATTAAC
TATGCTTTAAAATTACGAGGCATAGTCATAACGC
TAGCAACGCATGAGGACACTACGAAGGCACCAACCTAAAATTATACCAT
TACGTTGGGAATAGCGAACGCATAAAAAGCGGATTGTGGGAA
GTGTCTGGTTCGGTCGCATCGCCCGAGGCTTTTGT
TAAGTTTTGCCAGAGGGGGGGCAAAGCCAAAAAGATTA
TAGTAGGGCACGCTGAGGTCTGAGAGACTACCTCAGGTCA
TACATAACAACGCCAACCAGTATAAAGCCAACGT
TCAACATATAAAAGAAAATGTGAATTTCTTAATGAAACCATTGTTAAA
AATTGTAATTGGCGATTAAGTTGGGTAACGCCAGATACATAAT
AGTAATTATATAAAGTACCGACAGAATCGCCATATTAATT
AAAGGTAAATTCTTACATGTAATTTGGCATGATTAAAGT
TAGCAGAGAAAATACGGTTTTCCAAGGCTCCAAAAGGA
TCATCAGTAATAAGAGACTGTCCAGCCTTGAATAGGTTGG
ATGTTAGCCACCACGAAAAGAACTAGGCAGAGGCATTTTCGT
TCGGAATACCCGAATAAGTATATGCGTAATAAACA
CGCAAAGAAAACGTACTCCTTATTACGCAGTGGAAACGCAATAAAGTT
CTCAGGTTTTTGGTTTAGTACCGCACTATATGTATTTTAG
**H1 design oligo sequences (5′-3′)**
GCAGTAGAGTAGGTAGAGATTAGGCATGGCAATTTTCTGTATCTTTTGATGACTTAGC
GCAGTAGAGTAGGTAGAGATTAGGCAGGTGAAGGCCACGTCTTTCCAGACGCTAAACA
GCAGTAGAGTAGGTAGAGATTAGGCAGGGCGCTAGGGCGTATAGAGCTTGCGATGGCC
GCAGTAGAGTAGGTAGAGATTAGGCAACGTTGGTAAGAAACCTCACCAGTTAGCCCGA

GCAGTAGAGTAGGTAGAGATTAGGCAGCGGTCCGGCGTTGAGTAACATTGAGCGGAATTATC
GCAGTAGAGTAGGTAGAGATTAGGCAGACTTCAAACCAGACCTTTTTGAAGCAAACTCCAGCTGCGC
GCAGTAGAGTAGGTAGAGATTAGGCAGAATAGAACGGATTCTAACGCAAG
GCAGTAGAGTAGGTAGAGATTAGGCATGGCGAAAACACATTAAATGTGA
GCAGTAGAGTAGGTAGAGATTAGGCAATCACGTTGAAAATCTCCAAAGAC

Table S8. Staple sequences used for the T3 triangle 2 folding.

**Core structure oligo sequences (5′-3′)**
GTGCCCCCTGCCCGCTAGAGAGTTCCGGGGGTCCGTGGTGGTTCCGAA
AGAAAACAAAGAAGATGATGAAACCCCTGCCTGTACCAGGGATACAGAGCCAGA
GAGCAAAATTATTTGACGTATAATGATATAACTGAATTT
ACGGATTTCTTTGCTGCGGCTGGTAATGGGAAACATCAGAAAAATATGCTTTGA
GAATTATTAGTACCTTTTCAGGTTTAACATTGAAGCCT
TAGCAAGCAATAAAAGCCGTTTTTTTTTATTTTCTGCTGATGC
ATTTGAATTACCTTTTTTAATGGAAATTTTTAGTACATA
ACCCTCATAAATGGAAGGGTTAGAAATGAAAAAACGATTATTATTTA
GCACGCGAGCATTAATTGCGTTCCGGGCGAGCAAATCGTTAACGG
CGCGCTTAATGCGCCGCTACGAATCAGAATCGGAACCGGCGGGC
TGCGTAGAGCCACCCTACCGTACTCAGGAGGTAGCGGGGTTTACAAAA
ACATGTGGTGCTGCGGCTGAGAAGCGTGCTTT
TTTACATCACAGAAATCAAATAAGATAGCAGCTCTTACCA
TAAAAGAAACGGGGATGTGCTGCAACATAAAATAAATCAAGATTAGTT
CAGTACCTGGTGTATCCAGAACCGTCATTAACGCCACC
TACCGTAAAGCAAGCCCAATAGGATACTTCTGAATACGCACGGCCAGTGCCAAGC
ATCGTAGGAATCGTCAGATGAATAATCGGCTGGATAAGTCCTGAACAA
GCCATTCAGGCTGCGCAACTGTTGGGTTTTTAGGGCGATCGG
TTTCAGAGAACGTCAAAACCTACCATATCCCTACTATGGATCAGATGGGAATTAT
TGCCCGTACGGGGTCAGTGCCTTGCAATACTTTATCGGCCTAATCAGT
CATGAATTTTTGTATTAAGAGGCTTAACCTCCAAAGAACG
TCTTCGCTATTACGCCACGACGTTAGAGAATAAGGCGATT
CCGATTTAATATAATCGCCACGGGAACGGATAACCTCAC
AGGGCGAAAAACCGTCCGGAAACAAAGGAGCG
ATTGGCCTTGATATTCACAAACAACGCAGTCTGTATAGCTGCCGTC
TATCAGGGCGGGGAAAAGCACTAAGCGGGAGCAGACAGGA
CGATGGCCTTTGGGGTCGAGGTGTTCTGCCACGTTTTCA
ACAGGGAAGCGCATTATGCGGGCCAGACACTTTTTCACGGAATAAGT
CCCTCAGAACCGTTTGCCATCTTTTTTTTCATAATCAAAA
ATGGAAAGATAAATCCCCACCCTCAGAAAGCCACGTAAA
ACCGTCTGGTAATATCATACCTACCCTCGTTAAGGGCGCG
CAGAACAAACGGTACGGAGGCCACGCGCAGTGGTGTGTTCCGGTTGCG
GACGGGAGAATTAATTTTTTGAACACCCTGAACAAAGTCA
TCAATATTTTTAAAATTCAGCTGGCGAAAGGCAA
GACTTGCGCAAGAACGGGTATTAACTAATGCAGAACGCGC
ATACATGGAGTTTTAATAAACAGTTAATGCCTAAAGGTTCGCCTGAT
TAGAAGAACTCAAACCTTTGATTAAATTAACCGTTGTAGTTACACTG
CTGATTGTTTGGATTTCCTGATTTTGCTTTG
ACGCTAACGAGCGTCTACAGCCATTTTTGTTTCCGTGAGC
AATCAATATACAGTAAATACCAAGTTTGCTCAATTTCGGA
ACCAATTTTTGTACCGCACTCCGTTTTAGTTTTTGAACCTCCC
GGATTAGGATTGAGCCAGCTTTTTAAATCAC
CAACAATATCTTTCCTTATCATTCGGAGGTTTTACCGCGCCCA
TAGCCCCCACCTATTATTCTGAAATCAATTACTCGCGCAG
TTATTAGGAGTGTACTGGTAATACTTTTGATCGGATAAGCCGGAATA
AATCAATATCGACAATAAACAACTTTTTTGTTCAG

ATGTGAGTTTGGGTTATATTTTTACTATATGTAAATATTCATT
TCAATCCGGCGCTCACTGCATCAGCGGTCATAGCAGCAAGCGG
GTATGAGCCGGGTCACGGATCCCCCGCCTGTGCACTCATAGAGAGGGT
TCGTCACCCCACCAGAGCCTTTTTCCGCCAGCATTGACAGGAG
AGGTCAGACGATTTTGACGCTCAAGAAGGAGCATGGCAATTCATGGCAA
CTGAGCAAAAATTAATTACATTTAACAATTTCCTGTTTATAGGCGAAT
CGGAAGCATAAAGTGTCATAGCTGACAGTTGATGTTGCCC
TCGTCATAAACATCCCAGTAACAGTGTTTTATTGTCCAGTAAGCGTC
AACTGCATATAACAGTTTTCGCAAAAAAAGATGGTCTTTACAAAATA
AATAGGAATGTAGCCAGCTTTCATCAACATTGGCATAGT
AGACAATCTGGCCAAAAGAATACCTAACAACTATCTAACGGCCAGA
CTATTAGTCTTTGTGGCAC
CTGGAGGTGTCCAGCATCTTTTTGCGGGGTCATTGCAGGCGCTTTCGCAC
CCATCCCACGCAACCAGCTTACGGTAATGAATCAGCGTGGCCAGCG
CCACTACGAATACACTAAAACACGCCACGCTTAGAGAGT
TTGCTCCTCGCAGACGGACCCCCAATTAAACGGGTAAAATACGTAATG
ATCCTGTCCATCACGCAGTAATAACATTTTTCACTTGGAA
AGTACAACGGACTAAAGACTTTTTCATGAGGAAGTTTCC
CGATTAATTTTTGGGATTTTTAAACATTTTTGAGGCTTTGAGGA
GCGATTATACCAAGCGGTCCATTTTTGCGGATTAGCTCAAC
GGCTTAGAGCTTAATTTCATCTTTGTCAATCATAAGGG
TTCCAGTCGGGAAACCTTTTTTTCGTGCCAGCTGCAT
AGAGGCGGCGGTGGTGCAAGAATGCCAACGGCTCTAAAGCAGGAATTG
TTGCGGAACAAAGATTTTTACCACCATCGTCTGAA
CGCCATCAAAAATAATATTTTTGTATTATAGTACACGACC
AAAAATCATAAGAGGATTTACCAGGACAGATGAACGGTGTGAACGAGG
ACGAAAGAGCACATCCTCATAACGCCGTTTTTTTAGAGCCTATTAGAC
AACCGACTCAATATCTTGAGAGCCACACCGCCTGCAACAGTGCTGAATA
TAGAACGTCGGCCAACAGTGAGACGGGCAACAAAAAGAAT
ATTGCATCATGTAAAAGGGACATTATTTTTGAATGG
AAGTTTCAACCTTTAATCCAACAGTGAAAGAG
TTCATAGCCCTAAAACATCGCCATAGTATTAAGCAGCAAATGAAAAA
CCGAACGATCTGACCTGAAAGCGTCAGAGATATTTACAAACAATAAG
CGAGTAGTAAATTGGGCTTGATCAAGAGTAATCTTGGGCTTTGA
AAACGATGCTGATCCCAGCCCGAGGAGTCCACTATTAAAG
ACGATCCACGAGTAAAAGAGTAATATCTTTTTGGTCAGTTGG
CTTATAAATCGCTGATTGCCCTTTTTTTACCGCCTGGCCCTG
CAGCAGGCTTTTTAAAATCCTGTTTGATAAGCCGGCGAACGTGGC
GTGCCGGCCGGGTTTTTTTCCTGCAGTGCTGGTCTGGTCAGC
GCTGAACCTCAAATATCAAATTTTTCCTCACATCAGAT
TCGTCTCGCCTTTAGTGATGAAGGTTGGGCGGATTAAATCCTTTGAACCACCACACCCG
TGGATTATAACAGGAAAATTTTTCGCTCATGGAA
CCACGCTGGTTTGTGCCGTTCCGGCATTTTCACCGCGCGGGG
ATCACCTTGATAAAACAGAGGTGAGGCGGTCTAAAAATAAGGAAGGTTAATAGA
TATTACTTTTTGCCAGATAATACACAAATCAACGGACTTGAGCAACCG
CGCCATGAACGTGGACTTTTTCCAACGTCAAATCGGCAAAATC
ACGACGATAAAAAAAAAAAGCCGCCAAAGCGGTAGGAGCAAATGCGCG
GAACCCTTTACATTGGCAGATTTATCATTAATTTTAAAAGTTTGCTCACGGA
ACAGGCGGTCGCTGGCAGCACTGACCAACTTGTCAGGAT

TCCCGGAATTTGTGAGAGATAGACTTTCTCCGCGCAGAAA
GTGTAGCGGTTTTTCACGCTGCGCAAACTTAAATTTGGG
TTGTGTACAAAAGAGATGGTGAAGGGATAGCTTCGCGTCTGGCCTTCC
GTCAATAGCCATTGCACCACCAGCACCTGA
TTTACCAGAAGAATTTTTAGCGAATCGGCGAAACGTACA
CTTTTGCATACCAGTCAGCCCGAACAGACCGGAAGCAAAC
AAAATCTACCAAAAGGAATTACGAAAATGTGATACTGCGG
GCGAGTAACAACCTAACGCGTTAATAAAACGAACTA
ATCGACATAAAAAAATCCCGTAACCCCTGACTTAAATCAGTTAAAATT
ACTTTAATTTTTTCATTCTGACGAGCCAAATCAACGTAACAGCAGCGAA
GCGCATCGTAACCGTAATTGCGACGATGAACAGAGTCTGGAGTAGA
AAAGAAGTTTTGCCAGATTTTTGGGGTACCTTATGCGTTTTTTTTT
TTTAATTCAAAAAGGCTTTTTCACGTTGAAAAATAGTAAAATGAGATGG
CGATTGAGCAGTATGTCTTTCCAGCTGGTGCCGGAAACCAGGCAAAG
ACCAGAGCCATCGATAGGCCGGAATGGTTTGA
GCTTAGATCAGGGAGTTAAAGGCCCGGTCGCTGAGGCTTG
CTTTTTCAGTAATCAGTAGCGACTCCTCAAGAGA
AATCCAATCGCAAGACGGCTTAGGGAATAACCTTGCTTCTGTAAATCG
ATCAGGTCCGGTTTATAAGGAACAACTAAAGGGCATCTGC
GTAAACGTTTTTTAATATTTTGCTCATTTTTTATATTCATTGAATC
TCTCCGTGGGAACAAACAGGAATACCACATTCAACATTATT
AACAGTACGGGAGGGAAGACAGCCCCTACAACG
CATTCAACATCACCGTATGATTAAAATACCGAGAAAAAG
AGTACAAATCATAGTTAGCGTAACGATCTCAAAAGGGCGA
AGTAAGTCAATAACCTGTTTATTTTTCTATATTTTCTTTAGTTTGACCA
GCGCGAGCGCAAATATCCAAAAACAGGAAGATTTTCT
TAATATCAGTAGAAAAGAACTGGCCACCGACTCCATTACCAGAGCCGCC
GAGCCACCACCCTTGGTTTACCAGCGTTTTTCAAAGACAA
TCACCGGACGTTTTCATGCCTTTAGCGTCAGAATCAAAATTTAGTTAA
TAGATACATGATTCCCAATTTTTTCTGCGAACGAGCAAACCCGTTCTA
TTCTAAGAACGCGAGGATCGAGAACAAGCGAAAATTCTGTCCAGACGA
TAAAACTAGCATACGAGAATAGACAGCTTGCTTTACCTAAATTTAA
TACCCCGGTTGATACAGTTCAGAAAAGAGAATATAATAAT
TCCAAAAGGAGCCTTTACCGATAGGCTGATAAAATGTGTA
AATTGTATATTGCCTGGGTAATCGAATCATAATTTGGG
GCGTTAAAATCAGATATAGAAAAATACATACATAAAGGTGGCAACAT
AGACTTTTTTAAATATCGCGTAACGAGAATTTTTGACCATAAATC
CGGAACAACTAATTTTTTGCAGATACACGTCGGA
TTAGCAAAAAAGGCTGTAGCTAGGTGAATTTGAGAAGATAGCGATA
CATTATGACCTGCAACTATTTTTAGTACGGTGTCTGGTAATGCTG
ACGTCACCTCATTAAACGCACTAATAGTAGTAGCATTAAC
GGTAAAGTTAAACACCGGAATCATCCAGGCTT
CGACAAAACGAGAAAATAAATAAGCAGTAGCATGAGCCATTTGG
ACAATGACAACAACCATCTCCAAAGAGCTTTTTTTAAAGCGAAC
TGTTTTAAATACTGGGTAAAGATTCAAAAGGCATAACC
TTCTGCGATTTTTGAGCCCTCATAACGCAAGGATAAAAATCTGTAGCG
TCAGAGCCACCACCCCTCCCTCATTAGCAAGCAGCACCAATATATT
ATCCGGTAGAGGGTAATTGAGCGCCGCCATTCTTATTTG
CATAGGTCTAATTAATTTTCCCTTAGAATCCTGTATAAAGTTTCATC

GCCTGATATCGCCCACGGTGAGAAAGGCTTTTTGGAGACA
TGGGATAGTATCGGCCTCAGGAAGGAGGGGAC
ATTCCACAGGTAAATATGGCATCAATTCTCACCAGAACCA
TAATACTTTTGCGGGAGAAGCCTTCGGTTGTCGGAGAGG
GTATGGGTTTATCGCACTCCAGCCAGCTTTCCGGGTTAAGCCACTTTC
ACGGCTACAGAACAAGAACTTTTTGGATATTCATTACAAACACCAGA
GGAGATTTTTTTGTATCATCAGACAGCATCGGAACGAGGGTAGC
ACAGGTAGGGCTTGCCGTGAATTAAATTGTGTCGAAATCTTTTTGCGACCTGCT
GAAATTATAGATCTACATTAAGCAATAAACACCGGAACCGCTCAGAGCGGTGAATT
TACCTTTTGAGACAGAATCAAGTTTCGGCATTTTCGGTC
AATGAAACCACGCCTCAGAGCATAAAGCTAAATTATTTCA
TTTGATAAGAGGTAAATCACTTTTTATCAATATGATATTCAA
CCCTCAAATGCTTGTCACGTTGGTGTTTTGACCGTAA
GCCTGAGTATTAATGCACCAAAAACAGGCAAGGCAAAGAAATCCAATA
TATTTTAAATGCAATTAAGACGCTCTTAAACAGCTTGAT
ATATCCCATCCTACTCTTTCCTGTGTGAAATTGTTATCCGCTCACCTGT
TGCCAATTCCACACAACTAGAAACCGCTATTTTAAAGAAATAAGTTGGG
GGGAGAACTCATTTACGAGCATGATACGAGCTCCCAATCGCACCCAGCTACAACTT
TTAGATTTTATCCTGAACTTTACAGGTAAAACGCAGGGTTT
TTTTTAACTCACTCGAATTCGTAATCATGGTAAAGCCTGCTTCGCGTCACCCAAA
TCACTGCGGGGTACCGTGCCTGTTGGGTGCCTAATGAGTGAGCTCAT
TTTGGCCAGTTACAAAATAATTCCAGAGTGAACCATGTGGAGCC
CCAGAATCCCAGAATGCCTAAAGGTCAAGTTTCACTACGCCTAATTTTTCT
TCTTGGCTGGCTCTTAGCCGACAGACCATAATTTCAAAGAACTG
CGAAACAACCATGTTAGACCTTCAGATGGTTGGCGCATATCCT
TGAGATCATAACCCTCGAGGACGTTGGGAAGAGCTCATTA
GGCAAAAGAAGGCACCAACCTAAAAGCACCGTTTTGCGTATTGAAGT
TAACGGCGCCAGGGTGGTTTTTCAACGCGGTGAACGTGCCAGTTGAA
AGAAAGGAACTGCTCATTTGCCTGGAACAAATAGGGTTGAGTGTTGACCT
TAACTTCCAGTTGCCAGCAGGTAAAGTTTTTAGAAG
TGTCTGTAATTTAGGCATAAAGTACTCGCTATTGAGAGAC
GAGAGATAATAATAACCAGAAGGCCATATTTAACAACGCCAACAAGGT
TCAATTACGGAATACAATTACTACCGTGTGA
GACTCCTTGAAACGCAACCCACAAGCAGATAGCCGAACAAAGAAGT
GAGAATAGAGGCATTTTCGAGCCCCAACGCTCAACAAACT
TCTCGTAGGGCTTAATTGTATCATATGCGTTATT
AGTAATAACCTGTTTAGAGAATCGAAACCGAGTACCGAAGCCCTTTGGT
TAGGTTTAAGAAAAGTAAGAATTGACACCGCTTACGTTAGTAAATGAA
TTCTTACCATGAAAACAGTCAATAGTGAATTTTTTTAGAA
TAAACAATGAAATAGCAATAGCTATCTATTTTCAGCGGAGTG
TTGGTCAATCATATGCAGATTTTGCTAAACACAATAATAAGT
TTGCGCCGGATATATTGCTTTTGCGGGATCGTCACCCTCAAAGCTGCTT
TAGCTCAGTGAATAAAAAGATTCATCTAAT
TTGAGATTTGGCGGATTAGACTGGATAGTTTTTGTCCAA
**H1 design oligo sequences (5′-3′)**
GCAGTAGAGTAGGTAGAGATTAGGCACATCATATAACCCATGGTTGAGGCACGCCGCC
GCAGTAGAGTAGGTAGAGATTAGGCATCCCAGTCATACATTTTCAGGGATCACTGAGT
GCAGTAGAGTAGGTAGAGATTAGGCAGGCGCTAGGGCGCTCAGAGCTTGA

GCAGTAGAGTAGGTAGAGATTAGGCACGCATTAAAGTAACATCACCAGTCCAGTCGACAACTCGT
GCAGTAGAGTAGGTAGAGATTAGGCACAGCGGATCGTCCCGAACGTTATT
GCAGTAGAGTAGGTAGAGATTAGGCAAATCGTCATAAAACCGCGAGAGGAAGAGCAACACT
GCAGTAGAGTAGGTAGAGATTAGGCACCTGTAGCTTGTATAATGAAAAGG
GCAGTAGAGTAGGTAGAGATTAGGCAGACGACAGTAAAATCAGAAAAGCCTTAAATT
GCAGTAGAGTAGGTAGAGATTAGGCATCAAAAGTTTTGTCGTTAGCAAAC

Table S9. Staple sequences used for the T3 triangle 3 folding.

Core structure oligo sequences (5′-3′)
AAAATCCTGTTTGAAACAGTGTAGCGGTCACG
AACAAAATTTTCAATTTTTCAGGGACACTGAGATGATACAGGAGGT
ATCAAGAAACGCGAGGCGTTTGCTATCCGGTATTCTAAGAGCACTCAT
GCGCATCGGGAGAAATTTTAGACACCCTCAGTGAGTAAC
TTCCCTTAGAATCCTTGAAAACATAGTTTTTGATAGCTT
ATCAATATATGTGAGTTTTGCTCACACCCTCATTTCGGAA
TTGAATACGTACCGTAATAGCAAGCCCAATAGGGAGGTTTTTAACAA
AAACAAACGTTTTCACGCTGCGGCCAGAATGCGAATAACCCAAGTACC
AGAAGGATATTAAGAGGCTGAGACGCTGGTAACATGGAAATTCTTTGA
CACCGTACTCAGCAGCACCTTTTTTAATCAG
TCGATAGAACCCATCAAGTTACAAAATTGCAATAACAT
GGAGCGGGAAGAAATTCAGTTGGGCGGTTGTGTACATCG
GCCACGGGAACGGATAACCTCACCGGTTTTTAACAATCGGCG
AAACAGCCATAACCTAACGAGCGTTTTTCTTTCCGTTTGAAAT
CGCCTGTAGCATTATTATTCATTAAATTTTTGTGAATTAT
AAAAGAAGTGCTAGCGAACCTCCCATTACCGCGCCCAATAGCAGGT
CCGGGGGTTCCTCAAGAGCAATACTACCCGTATAAACAGT
GCGTAGATTTTCAGGTCAAAATTGCCGATTA
TTCTGCCATTCCTGTGGTCGGGAATGAGCTAACTCACATTCACCCAAA
GGTAAATTTTTATTGACGGTCCCGGAATTTGCAA
ACCTGAGCGATTCGCCGGAGAATTTTGAGCGCTAATATCAACAGCGGATCTCACGG
AGACGTTACAAATAAATCCTTTTTCATTAAAGCCAGAATGGAA
CTCTGAATTTACCATAAAAGGGACATGGAAGGGTAAAACAGAAGGGCGC
GTAGTTCCAGTAAGCGTCATACATTAAGTTTTAGCCACCACCCTCA
GCTCATTTCAAAGTCACAGTACCTTTTAAAAAACAGGAGATTTGCACGTTAGAAC
AGCGCCATGTTTACCAACGCAGAAGAGAGATATTCTCCGT
AGCATTGACCACCCTCAGAACTTTTTGCCACCCTCAGA
AGGAGTGTACTGGTAAGGCTTTTGTTTCGTCACCAGGGACAATAACG
AGTGCCTACATTTTGACACGACCATACGCCAGGGGAGCTA
AGCGCCAAAGATGAGAGATAGACTACCCACAAAAAAACAGGGAAGCGC
AGGGCGAAAAACCGTCACATAAAACGCGCTTA
TATCAGGGCGCTGGCAGTGGCGAGGAAGTGTTAGAGTCTG
CGATGGCCTGACGGGGAAAGCCGGGGGTGCCCCGGAAGC
GAATTGAGTTAAGCCCAAACGTACAAGGGCTTTTTGACATTCAACCG
AATAATAAGAGCAATTTTTAAACAATGAAATAGCAATAGC
CGCTCAATTCCATCACTTAGTAATATAGCTGTGCACGCGTCAGTGTCA
TAATGCCCATGAAAGTTAGGATTAGCGGGGTGGCGGGCC
AACAGGAAAAACGCTTATCCAGATCAAACTATCGGCCTT
TTCCTCGTTAGAATCAGAGCAATCCTGAAAAGGAAGCATACGAG
CTCCCTCACGGATAAGTGCCGTCGTGGAAACATTTCATTT
GAGCCGCCCTATTATTCTGAAACCCTGCCTAGAACCGCCACCCTCAT
TAATGAGACCTGTCGTGCCAGCTCCAGCGGCCTGTTCTTCGCGTC
TAGGAATCGACTTGCGATTAGACGTGATTGCTGGTGAAGG
ACCCTGAAAATTGCGTATGCCGCTCACAATTACCGTTGTAGGAACGG
CAGCCTTTGCTATTTTGCACCCAGTCAATAATCGGCTGTC
AGTTTTGTTCATAGTTAGCGTAATTTAACGTCAGATAGCTCAAACTTAAATTTCT

GTACATAAGTAAATCGTCGCTATTAATTAATTTTTCCTTAGAATTACC
TGAATCGGAGAGAGTTTAAAGCCTGCGTGGTGGTTCCGAA
TCAGACGATGCATTAAATTCGTAAATAAAGTGGCAGCAAGCGG
GGAGGTTTATTATTCATAATTACAAGTACCGCGTACCAGG
CTACATTTTTATTTTATCCTGCGTCAAAATTTTTTGAAAATAG
AGATTAAGATTTACGAGCATGTATTTTTAAACCAA
CGCTGAGATCATCTTCTGATTTTTCTAAATTTAATGTTTTTTAA
CTGCGCGCCTGTGCACGCTTTCCATGAAATTGTTATATGGAACCGCC
TCATTCCATAAATCAAGATTAGTTACAGAGAGCAGTTACAAAA
CGTCAGCGTGGTGCTGGTCTGGAAGGAAGCAAGCGCATCGGATAGGTC
CTGCGAACGAGTAGATGCGACCTGTCAAGAGTAATCTT
GTTTGGATTATACTTTTTTCTGAATAATTCTGGCC
GACAAGCGCTTCGACAACAACAGTTTCAATATCTGGTCAGTTTAGTTTG
TTTGGGGCGTGTCTGGTTTTAAATATTACCCA
GTACTATGGTTTTTTGCTTTGACGCCTCCGGCCAGACCA
GCGCAACAGTGCCACGCTGAGAGCACCCTCAAGAAAGGAATTGAGGA
TTTCGCACCCTGATAAGTACAACGGAGATTTGCATCCCTT
TGCCATCCCACGCCCCAGCCCGAGGAGTCCACTATTAAAG
CAAAGCGATCCTTTTGATCGTCATAATCAACGTAACAAAGGGCTGGCT
TCAGATGCCGGGTTACCTTTTTTCAGCCAGCGGTGCCGGTGCCCCCTGCA
AAATATTCATTGTCAGCAGCAACGGATTAGACTCGTATT
GTGTTCAGCAAATCGTTAACGGCAGCGCCAGGGTTGCCCTTCCCCG
CCACGCTGGTTTGAACCAGCTTACGGCTGGAGGTGGTTGCGG
ACACTGGTCTTTGCTCGTCATAAAAGGTTATCGAAGTATT
CTTATAAATCGCTGATTGCCCTTTTTTTACCGCCTGGCCCTG
CGCAAGAAGTCATTGCAGGAACCGGATATTCATGCAACTGAGTACCT
GAAACAAAATTGTGTCGAAATCCTGGCAAATAAAGTACG
GCAGCACCTGCCGGACTTATCATCATATTCACGTATAACGTGC
TGAACGGTGTCGGTCAATGCAAAAGAATACACTAAAACACTC
TAAAAATATTTGAATGCCTAAAACAAATCCTCAAACAA
CCAACGCGCGGGGAGAGTTTTTCGGTTTGCGTATTGG
AAAACAGATGAAAAGGCAGGCAAGTTAATTGCACCAGACCTCCCCCT
TATCATCGTCAATCCGCCGGGCGCGTCCAGCAACGTTATTGCGGAACA
AGAGGGTTTTTTGATATAAGTATATATATTTTTAAGGCGT
TTAAACAGACTGCGGAATAAGAGGGCTGTAGCTCAACATG
TTATGAGTAGAAGAACACAATATTACTTTTTGCCAGCTCA
AACTTTTTTATAGACGTGAGCCGAGCTCGA
AAATCTACGTTAATAAAACGATAAATTGGGCTTGAGACGAAAGAG
TATTATCTGGTGCCGGAAATTGCCAGATACATAACGCCAAAA
TTAGTGATGAAGGGTAAAGTTAAACGATGCTGCGTCTCGT
AATTTTAAATTATTTATCTAAAGCACATTG
CAGCAGCAAGACTTTATTGCCCGATCAGCGGGTGCCAACG
AATGAAAATGCGCGAACTGATAGCGCTATTAGAAGAAACCACCATCA
AGGATTTATAAAATATCCTTGCTGAACCTCAAATATCAA
AAGTTTCAGACCTTCACTCCATGTACCCCCAGCGATTATACCAAGCGC
TTAACACCGCCTGAGCGGTGAGGCACCACCAGCAGAAGAT
CGTCTGTTTTTAATGGAAGTTTGATACATTTGTATGAGCCAAAGGTTT
GGTACCTCCTCACATTTTTTTGAGGAGCGGCTGG
GAGGCCATTTTTCGAGTAAATTTATATTTTTTCAGTGTAACATT

ACAGAGATACATTGGCAGTTTTTTTCACCAGTCA
GGCGGCCTCCACATTTTTCCCGCAAATCCCGTAAAAAAA
TTGTAGAACGTTTTTTATTGCCGTTCCGGCAAATCGGCCTCAGGAAGA
CCGCACAAACGTGGACTTTTTCCAACGTCAAATCGGCAAAATC
GCACACAGACAATATTCCGAACGAGGTCAGTAATCGCCAT
TCTTTAAAGAACCCTTCTGACCCCTGATTTGATGGCAATTCATCACGCGGTC
ACACCAGAGGCGCATACTGCTCATGAACAACATAGGAATACCACAAAT
TCATGGTCAACATCACTTGCCGAGCCGTTTTTCAATAGATAA
AGGGGACGACGACAGTGTAGATGGACTCCAACTAAGAATA
GAATAAGTAACTAAAGCGGCCAGTGCCAAGCTTTCAGAG
TCATTTTTTTTGCGGATGGCTTCGCGTTTTTTTAATTCGAGCTT
CCAACCTAAAATGGTTTAATTTTTTTCAACTTTAATCGTTGGGAAGA
AAGAGGAAGCCCGCGATCGGTGCGGGGGTCAACTGTT
GCTCCAACGGGGAATTAACAACTTTATTTTCT
CCGACTTGAAGGCCGGAAAGAAACTACAAATTTTTAACA
GGGAAGGGTAAGTTGGGTAACGCCTGTGCTGC
CAGCTTTCATCAAGACTTCAAATACCAATAGGTGAATTTC
GTAAATGACAACAGTTTCAGCGGAGTGAGGAACACCGTCA
CGTGGAAGAATTAGCAAAATTTTTTTAGCAATAAAGCATTAACATCCAA
GCGGATTGTTTTTCCGTAATGGTAACCGTGCATGCATCAAAAAGAT
AAATCATATGGCATCAATTTTTTTTACTAATATTTTTTAAACAGGAAG
CACCATTATTGCATTATGACCCTGTATTTTAATCAAAAGGTTTTAGAA
ACCAGTAGTTGTTAAAGTGAGAAAGGCCGCCAGTAAAGATATGCAATGCCTGAGTA
GAGGCAGGTCAGACACCACCAGTTTGCCTTTTTTCGGTCCGGAATC
GCATAAAGCCGTGGGAGCGAGTAACAACCCGTATTTTT
TTAAATTTATAACCGACGGTTTATCAGCTTGCTGGCGAAA
TTATATAATTGAGGACTAAAGACTAACGGCTACAGAGGCT
TTTTTAAGTCACAATCAACATATAAAACGTCAGAATCAAGAGCCGCCGC
TCCTGAACTAAATAAGTATAAAGCTAGCGACACCAATGAAACCA
ACGATTTTTTGTTTAAAATCTTACCAACGAGTTAATATCCCATCCTAA
TTCCATATAACAGGTTGATATTTTTTCAGAAAAGCCCCAAAA
ATAAGAATATCTTACCGAAGCCCGTGGAGCCATTGAGGG
AAGAAAAAAGGGCTTAATTGAGAAGGTATCCAA
TTCTAGCTGATAACCTGTTTTTTTAGCTATATTTTCAACCATTAG
TTTGCTAAGAGCCAGCGTTGTACCAAAAAATATTCACAAA
TCAGGTCACGCGTTTTCCGGAGACAGTCAAATCACCATCATTTTTGAG
GAGTCTGGAGCAAACCTATATGTCAGGGAGTTAAAGGCC
AATGACAACAACCATCGCTTTTGCATTGTATACGATGAAC
TTATTACTTTTTAGGTAGTCAGGACATTGTGAATTACCTTAACGGGTAA
GCCCACGCTTGTTAAACAAAAATAGAAGCCTCCTCAGA
CAACGCTCATTATTTATCCCAGGCAATAGAAAATTCATATGGTTTAC
AAACCTTCCTGTAGCGGGGAAAATCTCCAAAAAAAAG
AGCGCCATTCGCCATTCAAAATAGCGAGAGGCTGAGGCATA
GCCTTAGCGTTTGCCACCACCACCGGAACCG
CCCTCATATAATACTTATAAAAAT
CACCCTCAGCAGCGAACGATAGTTAATTGCTTTTTGAATATAAT
ATGTGTAGCCAGAACCGATTGGCCCCATTAGCAGCCATTT
AGCCTGCTTTAATATTTTGCCTGAAGATCTACAAAGGCTAATCACCGG
AATAAACACATAGCCCCCTTACGGAATAGGTGTA

CCGACCGTGTGATAAAAGTTAATTAGAGTCAATAGTGAATTTATCAAA
AAGAGAATAGCAAATATTCAACCGTTATTTCAACGCAAGGTTGCGGGA
AGAACGCGGTCTGAGAGACTACCTTTTTAACCTACCGACATAGAAAA
CGCTATTACGCCAGCTTTCGAGGAACGCCATTCAGCTCAGTAGTAG
TTCAGAAAACGAGAATGTTTTTCCATAACATCAGTTGTTTTTGATT
ATTAATGCCGGAGAGGGTAGCTAATATGATATTTAAATT
CATAATTTTTGGAACCGAACAATACGTAATGCCACTACGAAGGC
TGACCAACAGACAGCAGCATGTCAATCATTTTTATGTACCGGCGCAGA
GTAAGAGCTTATACCAGAAAGATTTTTGAAAGAGGACAG
CAAATGGTCAATAAGGTAATCGTAAAACTATCGGAACG
GAATTACTTTGCTTTTTAAAAGAAGTTCCAGGCA
ATTCGCAGTAAACGGAGGCTTGAAATGCTGAGGTTGGG
GCCACCACAACCAGAGTCTTTTCATAATCAAACAAGACAAATAATTAC
CCTCAGAGCATCGGCATAGCGTCAATGCGTTAGCAAAGAC
AGAGCCTAATTCGCGCAGAGGCGATGAAGCCTAGAACGGGAGCAAGCCAGGT
TCATGTTTTTATTTTCATCGCGAGAACATATTAAACTTGCTTCT
ACTACAAGATGAATATACAGTAAGAGGGTAAAACTGAACCGAGGTGCTCGT
TCCCCGTAAAGCACTAAATCTTTGGAGCAAATCAGGGCT
TTTGATATAGAAGGCTTCACTGCCCTCTGTGGTGGTCATA
TTTGCCCCCGATTCAAGTTTGGAACCCTTGAACCATGCCGCCAG
GCAAATTACCACACAAGGAAGAAATTAGAGCTCACTACGAAAGGGAGTTCT
TACGAGAAATCTTTGTACTTAGCCGGAACGACCGTTGATTCCCAATTATACATTTC
ACAGACCAACGAGTAGACTAACGTCAGTGAATAAGGCTTT
TGGATAGCGTCCTTCAACTAATGCAGAGGGGGTAATAGTACCGCTTAGTCAGA
CCAGTTTGTCGCACTCCAGCCAGCTTTCCGGCAAAATGTTT
CTTTAGGAGCACTAACTAATGGGTGGGTCACTGTGGTTTTTCT
TCCAGTGAGACGGGCAACAAAAAGAATCAGCAGGC
TGCGCGTAAGCACATCCTCATATGGAACAAATAGGGTTGAGTGT
TCAGTTACGGAACGGTCGGTGGATCATTTT
GGGATCGTAGGGTAGCTTTTCATGAGGAAGTTTCCATTAATGCGATTTT
TCTGGCTCAAACACTATCTTGATACATCAAAAATCACCTCTT
TAGAGCTTGCGCCGACTTAAACAGCATAACCCTCT
TCAGACGACGATAAAAACCAGGCTGCGCTTTACCCTGA
TTAATAAGAGAATATAAAGTCCGGCTTATGCAAATCCAATCG
ATTCGCGTCTGGATCAATTGTATTATATTCGGGCATTTTCGT
TACGCAGTATAGGCAGAGTCGTTTAGTATCATGACTGTAG
TCACGTTGGAAGGGTTTTCCCAGTCACGACGTTGTAAGACTCT
TCAACAAGCTAATGCAGAACGCGAAAGGTAAAGTAAAATT
TTGTCCAGACGATGTAATTTGTTAGCAACCAAAGGAGCCTTT
CCTGTTTAACGCCAACACGACAATAGGAAACCT
TTACGAAGGCATGATTAAAACGAGAATTGCGAATAATA
TACATCATAGATAAGATCATAGAGAAAACTTTTTCAAA
ATACCCAAAAATACATTACCAGAAAACAACAT
TCCGAACAAAGTACATAAATCGCCATACTTACCAG
AACGTAGAAAGAACTACGCAATAATAACGGAAAAAGTAAGCAGAAACT
**H1 design oligo sequences (5′-3′)**
GCAGTAGAGTAGGTAGAGATTAGGCACTACCATACGATCTAAAGCGCAGTAAGTACAA
GCAGTAGAGTAGGTAGAGATTAGGCAAAAAAGAGGAACCACAGACAGCCCCGTCTTTC
GCAGTAGAGTAGGTAGAGATTAGGCAATGCGCCGCTACAATACGCTAGGG

GCAGTAGAGTAGGTAGAGATTAGGCAACGTTGGTAATATAATTGAAAGCGAGGGAAGGA GCGGAA
GCAGTAGAGTAGGTAGAGATTAGGCACGCTGGCAGAGCCTGATTATCAGA
GCAGTAGAGTAGGTAGAGATTAGGCAAGCAAAGCGGATTCTGCAAATGCT
GCAGTAGAGTAGGTAGAGATTAGGCATATGGGATCGGATTCTCTAAATCG
GCAGTAGAGTAGGTAGAGATTAGGCAAAGGCGATAAACATTAAATGTGAACAAACG
GCAGTAGAGTAGGTAGAGATTAGGCAAGGAATAGAAAGGAACTTATTTTG

Table S10. Staple sequences used for the T3 triangle 4 folding.

**Core structure oligo sequences (5′-3′)**
GTGAATAACCTTGCTTCTGTAAATCGTTTTCGCTATTA
CCGGGGGTAGGCGGATTGTTTTATTGTTCTGAAACATGA
ATATTATTGAGGCGTTTTAGCGAATAGATAAGTCCTGAAC
AACGGGTAAAATCAGATTAACGTCAACAGTACGGTGAAGG
TATAGAAGTGATTGCTGAGCAAAACAGAGCCAGCCCGGAA
GAGCAGGTTTAACGTACGTATAATAGGAACCTCGGAACC
TATCAGGGCGGGGAAAAGCACTAAGCGGGAGCAGACAGGA
AATAGCAATAGCTATTTTTCTTACCGAAGCCCTTTTTAAG
CCGATTTAGAACCTACCAGTTGGGCGGTTGTGTACATCG
CGATGGCCTTTGGGGTCGAGGTGGGGGTGCCCCGGAAGC
CCACCCTCTAGGTGTATCACCGTAATTAATTATGAAACAA
CAAGTTACTATACAGTAAAAATGAAAACACAAGAATTGAGACAGCGGATCTCACGG
AAGTATTAATTAGCGGTCGAGAGGGTTGATAGGCGGGCC
TAGAAGAACTCAAACCTTTGATTAAATTAACCGTTGTAG
GAGCTCGATGAATCGGAGAGAGTTTAAAGCCTCCGTGGTGG
AATTGCGTGCACCGCTCACAATTCTGAGAAGCGTGCTTT
TATTACTGGTAATATCATACCTACCCTCGTTAAGGGCGCG
TTCCGAAAGGGCGAAAAACCGTCACATAAAAAAGGAGCG
ATTCACAAACCACCAGAACCATTTTTCACCAGAGCCGC
TTTAGCACAACGCCTCGGGAGAAACAACCCTCCAAATA
GCCACGGGAACGGATAACCTCACCGGTTTTTAACAATCGGCG
ATCCTGAATCATATTAAATCAAGTTTTTTTAGTTAATATATTT
CTTTCAACAATTTACCGTTTTTTTCAGTAAGCGTCATACATGG
TAATATCCGGTATTCTAAGAACGCTATCCCAAAGCTACAATTT
TCAGACGATGCATTAAATTCGTAAATAAAGTGGCAGCAAGCGG
CCTCCTTTTTCGACTTGCGGGGTTACAAATTTTTTAAACAGCC
GCTCATTTAGAGAGAATGCGTAGATTTTCCCTACTATGGCTTCTGAAATAATCCT
CTGCGCGCCTGTGCACGCTTTCCATGAAATTGTTATGAGAAGCCCAA
TAACAGTGCCCGTATAGGTCAGTGCAGCCCTCATAGAGCCAGATGAA
AGCGCCATGTTTACCAACGCAGAATTAAGCCCTTCTCCGT
CCCTTAGAAAAGAACGCGATTTTTAAAACTTTTTCAAAAACAAA
AATTTCATTTGAATTATAAGTATACCACCCTCAGACTCCT
GGGATTTGTTAGTAAATGAATTTGCACGTAAAACAAGCTCAAACTTAAATTTCT
ATTAATTTTGAACGCGCCTGTTTTTTTTTCAACAA
CGCGCTTAATGCGCCGCTACGAATCAGAATCGGAACCATACGAG
GTCACCTTTTTACTTGAGGTCCCGGAATTTGTGA
AGAGCCGCAAGAGAAGGATTAGGAGAGGCTGATTTTCAGCGTAACAC
CCCTCAGAACCGTCAGACTTTTTTTAGCGCG
TTCTGCCATTCCTGTGGTCGGGAATGAGCTAACTCACATTCACCCAAA
TTCATTTCAATCATTACCGCGGCTTTATTTTCATCGTAGGAACCAATC
TAATGAGACCTGTCGTGCCAGCTCCAGCGGCCTGTTCTTCGCGTC
AATTACCTTTGAATACTGAGTTTCTCCACAGACCTTGAGCAAATAA
ATTACTGGTAATAAGTTTTAACGGAACAGTTACATGTACGGATAGC
GAGCAAGAAACAATGAAAACGTACCGGAAATTTTTTATTCATTAAA
GATACAGGAGTGTTTGACGCTCAATCATCAATTAATGGAAGGGTGGCAA
CATATCAAAATTATTGGATTATATTGCTTTG

GTAACGAGATGAAATAAAGAAATTAACATAA
GCGAATTAGTTTTCACGCTGCGGCCAGAATGCCCTTTTTTCATGTAGA
AAGTGCCGGGTTTTGCTCAGTACCCAATACTTTATCGGCCTAATCAGT
CTTTTACATGTAGCATGTCACCAGTACAAACTGCCACCCTGAAGATGA
CAGAACAAACGGTACGGAGGCCACATAGCTGTGCACGCGTCAGTGTCA
AAGGTAAATATTGAGAGATAGACTAATAATAAAGAAACGATTTTTTGT
CATTTAACCAGTACATAAATCAATATATGTGAAAGAAAAAACATCAAG
TGGATTATAACAGGAAAATTTTTCGCTCATGGAA
GCAGCAAAGGTCAGTAAGAGCCGTTATTAGACTCAGCGGG
CAATTCGACCATTGCGCCTGCAACACCTGA
AAAGAGGATTGACAAGGATTTTAACGCCAAAGTAAATTGTAGACTGAGACGACG
ACATCGCCGCAAAGAATACCAAAATGCTGTAGAGAGGTCAAAAAGAA
TTTCGCACAGCCGGAATCCGCGACCTGCTCCACATCCCTT
GAACCCTTTACATTGGCAGATTCATATTCACCAGAAGGAGCGGAACGCGGTC
ACATAAAAGGGACATTAATGCGCGTACCGAACGTGGCACAGACAATATTTT
TCAGATGCCGGGTTACCTTTTTTCAGCCAGCGGTGCCGGTGCCCCCTGCA
TGCCAACGGCAGCACCTGCCGGACTATCATTTTGCGAACCACCACACCCG
ATCATACAGACCATTATCTGCGAAGCTTGCCC
TGTCGAAACGAGGCGCAGACGGTCTGAACCTTTTAGTTT
GTGTAGCGGTTTTTCACGCTGCGGCCTCCGGCCAGAGGG
TGAATGGCTATTAGTCTTTCTGGCCAAAAGAATACTTAGAAGCAATAGA
GCTCATCGCTAATACATCAAATATCCTAAAGCATCACCTTGGGCGCGAG
CTATATTTTCATTTGGCAATCATAAACGTAACAAAGCT
CTTATAAATCGCTGATTGCCCTTTTTTTACCGCCTGGCCCTG
AATCAACAGTTGAAAGGAATTTTTTGAGGACGTGAGCC
TAACAACTAGTTGGCACCACGCTGAGAGCCA
ACCCTGACTATTATAGCACCGCTTCGTTTTAA
CGTCAGCGTGGTGCTGGTCTGTGCTTTTTGCGCGCATCGTATAGGTCA
CCACGCTGGTTTGAACCAGCTTACGGCTGGAGGTGGTTGCGG
ACACTGGTCTTTGCTCGTCATAAAATCTGGTCAATAGATT
TGTTACTTTCAATCCGCCGGGCGCGTCCAGCATTTACAAAGAACGTTA
TCATGGTCCGAGTAAAAGAGTTTATCTTTTTTAAATATCTTT
TATTACTTTTTGCCAGCAACTCGTAGGAGCACTATGAGCCAAAGGTTT
GAACCACCAGCAGAAGATTAAAAAAACTGATAGCCCTAAA
CCAACGCGCGGGGAGAGTTTTTCGGTTTGCGTATTGG
AAAATCCTGTTTGATAAGCCGGCGAACGTGGC
CCGCACAAACGTGGACTTTTTCCAACGTCAAATCGGCAAAATC
CGATTAATTTTTGGGATTTTTAAACATTTTTGAGGCATTAAATC
CAGGAAGAAGGGGACGACGACAGTTAGATGGGGATGGCTTACACGACC
AACCGGATAACGAGTAAGGAATTACGTTTACCGATAGCGT
AGTAACATTTGTAGAACGTTTTTTATTGCCGTTCCGGCAAATCGGCCT
AGAGGGAAATAAAAACGTTTTAAAACAGTTGATTCCCAAT
CTGGTGCCGGAAATCTTTCCAATACTGCGGAATCGT
GATACATTCCCAAATCAGGGAACCTTTGTATCATCGCCTGATAAATTG
GGTACCTCCTCACATTTTTTTGAGGAGCGGCTGG
CAGATGAAATACCAAGCGCGAAACAAAGTACCCTTATGC
TTAGTGATGAAGGGTAAAGTTAAACGATGCTGCGTCTCGT
CTGATTATCAGATGTTTTTTGGCAATTCGTCTGAA
TTAACACCTCTGACCTGAAAGCGTCAGAGATATTAATTTTAAAAGCT

TCAACTAATGCAGATACATAAGAACTGGCTCATTATCAGCGATT
CAACTTTGCGGATAACCTGTTTAGGTGGCATCA
GGCAAGATAAAACAGAGGTGAGGCTGAAAAATAAACCCTCAATCAAT
AGGCTGTCCATCACGCAGTAATAACATTTTTCACTTGGTG
CGCAAGAAGTCATTGCAGGTCAGTGAATAAGCGAGTAGAGAATATAA
TGCCATCCCACGCCCCAGCCCGAGGAGTCCACTATTAAAG
CGAGAGGCTTTGTCAGCAGCAACTAATTGCTTTGAGGAT
GTGTTCAGCAAATCGTTAACGGCAGCGCCAGGGTTGCCCTTCCCCG
GGCGGCCTAAGAATTTTTAGCGAAAATCCCGTAAAAAAA
CGCTATTACGCCAGCCGACAATGACGCCATCCAGCTCATAGCCTCA
TAACTATAAGACGCTGAGAAGAGTCAATAGTGCCGACAAAAATAAGG
TCCTCATTAAAGCCCAGACGATCGGTCATAATCAAAATTACCGACC
GCAAAGCGGATTGCATCTTTTTAAAAGAACTATCATATTTTTCCCT
ATAAATAAAATCTTTTTAAAAATCAGGCCAGGCA
TCTAGCTGATGTAGTAGCTTTTTTTAACATCCAATAACTGAAAAG
TAAATTTTCCGCTTTTATACCGATAGTTGCGCTGGCGAAA
TACCATTATACCAGCGCCAAAAAACGGCCAGTGCCAAGCTTTCAGAG
TTAGTATCTTACCAACGCTAATTTGACATTCAACCGATTGAGGGAGG
CAAAAGGGTTCGCATTAAACGTCTCAGCAGCTTTTTAAAAAATCAT
TTCGCGTCTGGCCAAACAGCTTGGCGGGATCGTCAATAAGAATAAA
AGAGAATCGCAAATATTCAACCGTTGTGTAGGTAAAGATTGCAATGCC
GACGACAAATTTAATGAAGCCTGTTTTTCATCATCAAGTTTGCC
AGAGCCTAATTTGCCAAGGTTTTGAAGCCTGCATGTTCAGCTAATGCA
CGGTGTTTTTACAGACCAGGGCAAAAGAATACACTAAAACACTC
AGGAACAAGGAAACGTATAAAAATTTTTACGCAG
AAGCAAACTCCAACGATCGGTGCGGGCCCCAACTGTT
GGGAAGGGTAAGTTGGGTAACGCCTGTGCTGC
AAACGAACGGCTTTGACCGATATAAGTTTCTTTTTTTCCATATA
CGTTAACCTAATATTTTGCCTGAGGATCTACAAAGGCTATCGCCACCC
GTTAAAGGTGTTAAATAAAAATAATGAGTAAGAAGCCT
CGCCAGCATCAGAACCCCGCCTCCCTCAGAGCGGGTTATAGTGTGATA
TCTTTGACCCCACCAGTCATTTTTGACGTTGGGAAGAGAATACCACA
TTCTACTAATAAAAGTAATCGTAAAACTAG
TCGCAAATGGTCATTGATAATTTTTCAGAAAAGCCCCAAAAA
ATCGGTTGTTAGCAAAATTTTTTTAGCAATAATTTTTAACCAGGAAGA
CTCAGGTTTTTGGTTTAGTACCGCAATCCAAT
AGTTAATTTCATCTTCCGCAAGACATCCTTGAAAACATAGCGATAGCT
CCGAACAAAAAAGGGCTGTCACAAGCGACAGAGGCATTTTTGGCCTTGA
TTATTTCACGTGGGAACGAGTAACAACCCGTCGCCTTT
CGAGGCATTTTTTAGTAAGATTTAGAAAATCTACGTTAATACGAAAGAG
AGTAATTATGACCCTGTAATATTTTTTTTTGCGGGAGAGCATAAAGCTA
TGTCTGGATTCGGTCGCATCGCCCACGCATAATTAAGAGGAAGCCTCTT
AGCGCCATTCGCCATTCAAACGAGAATGACCATTTCATTGA
AATTGTAGGAAGGGTTTTCCCAGTCACGACGTTGAACGCAATTTGCTT
TCTCTGAGTTTCAGATTGCGAATAATAATT
ATCCCCCTATCAGTTGAGAGCAACCTGGCTGACCTTCATTTTTTAAGAGTAATC
TCGAGGGTTGCAAGGCCCTAAAGGACGGAGTGA
TGACCTAATAAACAACGTTATACAAATTCTTATTACGAGC
TGAAACCATGTTAAAATGAGAAAGGCCGGTTGAGGCAGGTAGAATGGAAGCACCGT

TTAATGCCGGAGAGGGTAGCTATTATGATATTTAAATTG
ATTAGCGTTCGATAGCAAGGAACCCTCATATATTTTAAAT
AGCTTTCATCAACGTCAGGATTAGCAATAGGAACAACAAC
TATGCATTTTTCTAAAGTACGGAGAGTACCTTTTTTAATTGCTCC
CGCATAGGGGACTAAACATGTCAATCATTTTTTTGTACCC
CGGATTGATTTTTCGTAATGGGAACCGTGCATCGCGAACCAGACCG
TTGCCATCAGGAGACAGTCAAATCACCATCAATTTTGAGA
CTGATGCACACGCCACCACCGGAAGCCACCCTCAGAGCC
GTTTGAAACACCGGAACCAGACCTCAGAACCGCC
TCTAAAGTTTTGTTTTGGGAATTAGATTTTTCCAGCAAAA
TTTTCACCTCACCAGTAGCACCATAATCAGTATCAATAGA
TAGCAACGCATGAGGACACTACGAAGGCACCAACCTAAAA
AGTCTGGAGCAAACAGAGACTACCGAAAGACAGCATCGG
AGGTCTGAGTAAAATACGTAATGCAGTTTCCATTAAACGG
CTGAGGCTTGCAGGGAAACGAGGGTTGTATAAGATGAACG
GTCTTTCCAAAAGTAAGCAGATAGGTGGAGCCGGTGAATT
TTGACAGGTTTTCATAGCCCCCTTCACCGGAAAAATTCAT
GCTATTTTGCATAACGGATTCGCCGCTTATCCCATCCTAAGCTGTT
TTTATCATTCCAAGAATAATCGTTTACGAGAATGGAAA
AAAATCGCTGCCCCAATAGCAAGCTTAAACCATAATATCAT
AGATAACCCAGGGAAGCGCATTAGACGGAGGGTAAGCCTTTAC
TCAAGCCGTTTCACTGCCCTCTGTGGTGGTCATA
AAATAGCATTGAGCGCAGTACCGCACTCATCGAGT
TCTGAACAAAGTCAGGAGAATTTGAACCATGCCGCCAG
CCAGAATCCCACACAACCTAAAGGTCAAGTTTCACTACGAACTGT
TTTTAATCATTGTGAATTAAACGGAGAGAACTGAC
TTTTGATACTCAACATCAAAATAGTGACGAGAATGGTTTAATT
TAAAATGTTGGCTTGAGAACACCAGATTCATTA
CAGTTTGTCGCACTCCAGCCAGCTTTCCGGTCAGGGTT
TAATGGGTGGGTCACTGTGGTTTTTCT
TCCAGTGAGACGGGCAACAAAAAGAATCAGCAGGC
AGAAAGGAAGCACATCCTCATATGGAACAAATAGGGTTGAGTGT
TCAGTTACGGAACGGTCGGTGGCTTTGCCC
TTAAACAGTAGGGCTTAGTCCAGACTAGATTATGTAAATG
AGTTACCACACCACGCAACATATCATGTAATAAGCCAACGCTCAGAAT
TGTTTGGGAATAAGTCCAGTATAACTAGAAA
CGCAAAGAGAAGGAAGAAAATACATACATAAAGGTACT
TCATAATTTTAGGCATAATTCTATTGAGAATCGCCATATTTCAGT
TAGCAACTTCGAGCCAGACTCCTATGTGAATTTCTTAA
AGGTAAAGGAGGCATTAACGCCAAAAAAGAAATATTACGCAGTATATTT
TCTTGTTAGCAAACGTAACCGAGGATAAAACGAAAGGCTCCAAAAGGA
TATAAAGTAAATTTATCCCTCCGGCTTAGGTTCAGGTCAT
TGAGCCAAAAGAACTGGCATGATTAAGTAATAAGAGTTCT
TGTAGCCGGGTCGGTTTATCAGCAATAACGGAAT
GACTTTTTGCTACAGATAACGGAACAT
TTGTTTATTACAGGTAGAAAGATTCCAAATGCTTTATAGT
TAGTTCAGAAGGCTGCGGAAAGACTTCATTTTTATATCG
**H1 design oligo sequences (5′-3′)**
GCAGTAGAGTAGGTAGAGATTAGGCAGATTGTTTTTCTGTATCTTTTGATACGTTAGC

GCAGTAGAGTAGGTAGAGATTAGGCAAAAAAGAGCCACGTCTTTCCAGACTGCTAAACA
GCAGTAGAGTAGGTAGAGATTAGGCAGGCGCTAGGGCGCTTAGAGCTTGA
GCAGTAGAGTAGGTAGAGATTAGGCACGTTGGTGATTATCATCACCAGTCAGAGTTTG
GCAGTAGAGTAGGTAGAGATTAGGCACGCTGGCACGTGAACAAAGAAACC
GCAGTAGAGTAGGTAGAGATTAGGCATTCGAGCTTCAAATGCGTTTTGCC
GCAGTAGAGTAGGTAGAGATTAGGCAGAATAGAAGGATTCTCACGCAAGGCACCTTCC
GCAGTAGAGTAGGTAGAGATTAGGCAAAGGCGATCAGATTAAATGTGAGCAAACGG
GCAGTAGAGTAGGTAGAGATTAGGCAATCACGTTGAAAATCTCCAAAGAC

Table S11. Staple sequences used for the T3 triangle 5 folding.

Core structure oligo sequences (5′-3′)
TACCGCTGGTAATATCATACCTACCCTCGTTAAGGGCGCG
GCGAAAGGGGGATGTGCAGTGCCATAGCAGCCCCAGGGTT
CGTCCGTGTCATTAATTGCGTTGCCGGGTGATGCCGGGTTACCTG
CAGGGAAGCGCATTTTTTTGACGGGAGAATTAACTGAACA
CGCGCTTAATGCGCCGCTACGAATCAGAATCGGAACCATACCGG
AATCGTCGTGATGCAAATCTTTTTAATCGCAAGACAAAGATGAT
CGATGGCCTTTGGGGTCGAGGTGGTTCTTCGGGGTTTCT
CCAGAATCTTCACGGTCCTAAAGGTCAAGTTTCACTACG
AGTACATAAATCAATATATGTGAGTGTTTTTATAACCTT
TGAACCATATAACCTCTAATGGAAGGGTTAGATCAATATTTGCTTTG
GAGAATAACATAAAAAGCCAGCTGATAAAATTTTTGAAACGCAAAGA
GCAAGTTTTTCCGTTTTTATTGAGGTTTTTTTTTAAGCCTTAA
CACCCAAAGAGATAAAGAAATTGACGTATAATTGATATATCTGAATT
TGGTGTGTTCAGCAAGAGTAACATGTTTTTATTGTTCCAGTAAGCGT
CCCTGCGGCTGGTAATCGAATTCGTGCGGCCAGAATATACGAGAGGG
AGAGCCACCACCCAATAGAAAATTCATTTTTATGGTTTAC
AAATAATAATTAAACCAAGTACCGTAGTTGCTATATAGAAGGC
CACCCTCCACAAAATTATTTGCATTTGTTTACCCAATCCAAAATAAA
CGCAGAGGATAACGGAAGGTGTATTCAGAACCACAAACAACGCCACC
CATACATGAAGTTTTAATAAACAGTTAATGCTAAACATC
CCGATTTACTTCTGAAACCGGAAACAATCGGCGAAACGT
ATTTCATTAAACAAAATTAATTACCCCCTGCCAGTACCAGATGATACA
TCCGAAAGGGCGAAAAACCGTCACAGCGCCAAGGAGCG
ACTTGATTTAGTACTACAGTAACAGTACAGATTTTGCA
AAAGGTGGCAAAAGTTGGGTAACGTTTACAGACCCAGCTACAATTTTA
AGCAAGCAAATCCTTTTACATCGGGAACGGGTTCCCATCCTAATTTAC
CGGTATGAGCGCTCACGTGTCACTGCCAGCACGCAGCAAGCGG
GTGCCCGTACGGGGTCAGTGCCTTCAATACTTTATCGGCCTAATCAGT
CAGAACAAACGGTACGGAGGCCACGTGCACTCCGGCATCACACTGTTG
GCTTCTGTAGCAGAACGCGCCTGTTTTTTTATCAA
TTCGTCACCCGCCACCAGATTTTTCCACCACCAGAGCCGCCGC
TATCCGGTATCTATCATTACCGCTTTTTCCCAATAAGAACGCG
ATCAAGATCACTCATCGAGAACAACAATAGATAAGTCCTG
AGGATTAGGATGCCATTTGTTTTTGAATTAG
CATTCCAAGAGAAACACGAATTATTTTTGCTCTATTTCGG
TTTATTTTTTTTGTCACACTGCAAGGCGATTCAT
CCAAGTTACCTTACACGTTTCTTTGCTCGTCAATTTAACAGAGCATGT
GATGAATACGCCACCCCACCGTACTCAGGAGGTAGCGGGGTCATTTCA
CGGAAGCATAAAGTGTAATTGTTAACCGAGCTGGGTAAAG
TTGGGAAGGGCGATCGGTGCGGGCCTTTTTTTTCGCTATTAC
TATCAGGGCGGGGAAAAGCACTAAGCGGGAGCAGACAGGA
CCCTCAGAGCTAGCCCCCTTATTTTTTTGCGTTTGCCATC
TAGAAGAACTCAAACCTTTGATTAAATTAACCGTTGTAGATCGTTAA
CATCAAGATGAATTACCTTTTTTAATGGAAACAACAAGAAAGCAAAAG
CGGCATTTAACCTATTATTCTGAAGAAACAAAATTACCTG
CCAGCGCATGCCCGCTAGAGAGTTGCGTGCCTCCGTGGTGGT

GTACCGTATAGCAAGCCCAATAGAACCTACCATATCGACGAGGTGGAGCCGCCAC
ACAGGAGGTTGAGTTGACGCTCAACTGATTATTTGTTTGGATTTGGCAA
GGGAACGGAACGATTTCGTAAAACAGAACCCTACTATGGAATCCTGACAGATGAT
TCGGTCAGGAGTGTACTGGTAATGCTTTTGGCGGATAACCCGGAATTTCGCCTG
CGTGCTTTATTGCAGGTCAGACGATTGGCCTTAGCCAGAAAGTATAGGTGCCGT
ATAAATCCTCATTAAGATATTCGCCACCCTCAGAAGCCGTAGATTATTGCTT
CTTATAAATCGCTGATTGCCCTTTTTTTACCGCCTGGCCCTG
TAGAAGTACCATTGCTGAGGCGGTCCCTGA
GACGATGGTGCCGGTTTTTGCCCCCTCCGCAAGAATGCCAAC
TCTAAAATAACCCTCAATTAACACCGCCTGC
GGGGTCATTGCAGGCGCTTTTTTTCGCACTCAATCCGCCGGGCGCGGTTG
GAACCGATAATAGATTAAAAGCATCGAGCCAGCAGCAAATGAACATGTT
GTGTAGCGGTTTTTCACGCTGCGTTGCCGCCAGCAGGGG
AACAGTGCGAAGATAAGAGCACTAAATACATTCCGGCCAG
TATAATGCTGTAGCTCCTCATCTTGGTCAATCATAAGG
CGGCAAACGCGGTCCCAGCCCGAGGAGTCCACTATTAAAG
TGATTCCCAAGAGGTCGTACCTTTTTGAAAGA
AACCTGTCCATCACGCAGTAATAACATTTTTCACTTGAGT
CTATTATACAAATATCCCAAAATAGGACAGATGAACGGTGGGAACGAG
GTCTGGTCCGGCCAACAGTGAGACGGGCAACAAAAAGAAT
CATCAACATCTGGCCTTCCTGTAGTTTTAACCATTGCATCACACGACC
GAACCCTTTACATTGGCAGATTCCAGAAGCATTTTGCGGAACAAAAACAGCG
TGGATTATAACAGGAAAATTTTTCGCTCATGGAA
ATCGCCATTAAAATCTCTGATAGCGGCTATTAGTCTTTAA
AACGAAAGTGCCGGACTTGTAGAAGGCAGCCTTGAGGATTACTCGTAT
GAGCGGAATTATCATTTTTCATATTCTCGTCTGAA
CGTTATTAAATCCCGTGATCAAACGGAAAAAGAGACGCAGCCAGCTTT
TATTACTTTTTGCCAGTTAGACTTGAAGGTTATGGTGCTGGGCAGCAC
TCTCCGTGGGAACATAACACGTTAATAAAACGAACT
ATTCGCGTTAAATGTGAGCGAGTAACAACCAGGCATA
TTCCAGTCGGGAAACCTTTTTTTCGTGCCAGCTGCAT
AAAAATCTGCCAAAAGGAATTACGCGTCGGATATTCATTG
AGAGGCGGCCAGCTTACGTCGGTGGTGCCATCAAATATCAATCTTTAG
AAAAAAAGCCGCACAGGCGGCTTGCAAAGCGGAATAGGAAGTTAAATC
AGCGATTATACCAAGCATTCTTAATTGCTGAAGCAACTAAA
GTTAAACGATGCTGATCATAAAAAATTTTAAAAGTTAACCACCACACCCG
ATTTTTGCGCGCAGACTGACCCCCCATTAAACGGGTAAAATACGTAAT
TGCGCGAAGCGAACGAGTTTAGCTAAAGACTTGTCAGAAGCAAAAGA
ATATAAAAGGGACATTTTTTGAATCCTAAAACGTGGCACA
AATTTGTAACGTGGACTTTTTCCAACGTCAAATCGGCAAAATC
CCACGCTGGTTTGCCGTTTTTTCGTCTTTTCACCGCGCGGGG
ATCAATATCTGGTCAGTTGGTTTTTAAATCCAGCCAGC
AAGTACAAAGGACTAAAGACTTTTTCATGAGGAAGTTTC
AACAGAGGTCTGACCTGAAAGCGTCAGAGATATAAATCCTTTGCAAG
GATAAAAACAGGACGTTGGGAAGGGCTCATTGTAATAGT
CAGAGGGGATACCAGTGCGTTTTAAGGTCAGGATTAGAGA
AGGGTAAAAGCACATCCTCAACTGACCAACTAATTGCTCGAAGCCCG
GCGCGCCTCGAGTAAAAGAGTAGTTGATTTTTAGGAATTGAG
AATATACCGAACGAACCACCAGCACACGCTGAACCTTGCTGAACCTC

GCCACTACGAATACACTAAAACAAAAAATCTCTTTTGAT
ACGAGTAGTAAATTGGGCTTGATCAAGAGTAATCTTAGGCTTTG
CGATTAATTTTTGGGATTTTTAAACATTTTTGAGGCTACAAACA
GAGAGATAAAGAATTTTTAGCGAATGTTTACCAGTCCCG
GACAATATCTGGCCAAAAGAATACAATAGATACAACTAACGGAACG
CAGCAGGCTTTTTAAAATCCTGTTTGATAAGCCGGCGAACGTGGC
CGGCTGGAGGTGTCCAGCATCAGCTAATGAATAGCAGCAAGCATCA
GACTTTCTCCGTGGTGAAGGGATAGCTCTCACTTAAATTT
TACAGGTAAGGCTTGCTGTGAATTAAATTGTGTCGAAATTTTTTCGCGACCTGC
TAATGTGTAGGTAAAATTTATCATTCTTAAACAGCTTGA
AGCCCCAAAAACACCATAAATCAACGTAAAACAATAATAA
TTTATTTCAACGCAAGGATAAAAGACCCTGTGAGATCTA
TTAGCGACCCTGAACAAAGTCAGCGCAACTGCACCACGG
AAGACAAACATTAAAGCCCAAAAGTTAAATAATTATACA
TACGGTGTCTGGCCAAAGGCCGGAGACAGTCGCATAAC
AAAATGTTTTTTTTGACTGGACCTTATGCTTTTTATTT
CAGTACAACTCATAGTTAGCGTAACGATCAAGCAGCGCCA
AGACTCCTTCTTTCCAAGCGCCATTCGCCATTCAGGCTG
TGGAGCAATCGGTTTAAAAGGAACAACTAAAGCGACGACA
TAATTGTAACAAGAGACAATCATAATTAGCAGTGGCAT
CAATTCTATTAATATTAAGCAAATATTTAAATATTTTC
AATAGTGAGCAGGGAGTTAAAGGCTCGGTCGCTGAGGCTT
CAGAAAACGAGAAGCATCGTAACCGTTACGTTGGTGT
CATTCCACGATTGAGGAGTAGCATTAACACACCCTCAGAG
ACGGAACAACTATTTTTATGCAGATACAAACGGC
GCCGGAAACACCGGTTGTACCAAAAACATTATATTTTTAG
CGCCTGATATCGCCCACAAATCACCATCTTTTTATATGAT
CATCTTCTATCGATAGCAGCAGACTCCTCAAGAG
CTCCAAAAGGAGCCTTTACCGATAGGGTAGCTAGGGTGAG
CAACAGTTAATTCAACCAGACAGCCACTACAAC
TTAGCAAGATTGACGGCACTCCAATAAATCATACAGGCAA
AGATGGGCACTCCAGCCAGCTTTCGCCTCAGG
GGTTATATTGACCGTAATCAGTAGTGTAGCGCGTTTTCA
ATCAAAATCGTCACCACATTACCAATAAGGCGAACTGGCA
ATATTTTACAATAAACAAAGCCTGTTTAGTATATAGGCGTT
TACCGTCGTATCAGGTTGCCTGAGAACCCTCATATATTTTGCCTTTAG
TGCGGGAGAAGAAGTTTCTTTTTTTCCATATAACAGTTTAAATAT
AGTAATTTTCATTTGGGGCGCTTTTTAGCTGAAAAGTTTCGCAAATGGT
ACATGATTTTTAGTATTAAGAGGCAACTATAT
GATTCAAAATTTTTGAAATACTTTAAATTAAGCAATAAAGGGCAAAGA
TTTTCATACGTCAGACCGACAGAATCAAGTTTCTTTTTAATTTAATGG
GATTGACCGTAATGGGATAGGAATACCACATTCAACATTAT
GAAAACTTTTTCAAATGTAAATGCCTATTAATTAATTTTCCCTTAGAA
AATAACCTGTAGATTTAGTTTTTTTGACCATTACGGTAATGCCGGAGA
AACTTTATTTTTATCATCCTGACGACCCAAATCAACGTAACAGCAGCGA
ACCTCCCGACTTGCGGTTCATCGTAGGAAGAAAACATGTTCAGCTAAT
TCAGAAAGTAGATTTTGCTAAACAACTTT
CATAATTATCTAAGAACGCGAATAAGCAAACGTAGAAAATACATACA
CCTCAGAGGAGAGTCCAAAGGCAGGTGAATAAATCATAGAAGAGTC

CATAAAGCTAAATCGGAACCAGAGCTCAGAGCAAATTATTAGGGCGAC
GGATGGCTTAGAGCAACCGTTTTTTCTAGCTGATAAATTAAT
GACAATGACAACAACCATCTCCAAGGAAGCTTTTTAACTCCAAC
TGTACCCCGGTTGGAGAGAATAGTCAGCTTGCTTACCGTGTGATAA
AACGGCTACAGGACAAGAATTTTTCGGATATTCATTAGAAACACCAG
GCCAGTTTGAGGGGAGAATTGCGTAGCATGTATCGATGAAGATACA
AATTCGCATTTTTTAAATTTTTCGCCATCAAAATGCTTTAAACAGT
GACGACGAGTTAATTTACCGGAATAGCCAGCATCACCGTCACCG
ACCAGACCAAAAAAGGTTTTTTCACGTTGAAAATAGCGTCCAAGCATCT
CGGAGTTTTTTTGTATCATAAGACAGCATCGGAACGAGGGTAG
ATTCGATTTTTCTTCAAAGCGAAAATCAGGTTTTTCTTTACCCTGA
AGGGTAATAGTATGTTACGGAATAGTGAATTAAAATCACCGGAACCGCC
CCTCCGGCAAACATAGCGATAGCTTAGATTAACTTAATTGTTTGAAA
TCAGAACCGCCACCCCACCACCAGTAGCACATGAAACCGACCTAAA
CAAAATCGAGAAACCAATCAAGGTTCCGCTCACAATTCCACACTAAT
TGAGAACTTCCTTATTCCTGAATTAACGTCATTCCCAGT
CCGTAATCGGCTGTCTATACGAGCCAGCCATACTTACCAACGCTAGTTT
TAGCGTCTTTCAATGAAAAAGCTTTCAGTTGTAAA
TGGGTAACTCACATAGCTGTTTCCTGTGTGAAAAGCCTGTCACAGTT
TGTGGTGCTAATCATGGAGCCTCCGGGTGCCTAATGAGTGAGCGAGT
TAGCTACAAATAAGAGAGGATCCTGAGCGGCGGGCCGTTCTGAGAAG
TTCAGGTTTTATTTATACGTCAAACAGAGCCTAATTTGCCAGTCCGT
TCTGGCACTTAGCCTACAGACCTTAATTTCTAAGAACT
GCGAAACATCCATGTTTGACCTTCAGATGGTAGGCGT
TTTTACCTTCTTTAGTGATGAATTAAGAGGAGCCGTC
GCGAGAGGCAGACGACAACACTATCATAAT
AGGCAAAAGAAGGCACCAACCTAACCACGCAATTTGCGTAT
TGCCAGGGTGGTTTTTCTCGTCGCTCGTCAGCG
AGAAAGGAATTGGGCGGTTGTGTGGAACAAATAGGGTTGAGTGT
TCAGTTTACATCGATGCCGTTCATTCGACA
TTTTTGAGCAAGAAACAATTTTTAAGCCAGGCAAGACGTTAGTAAATGA
TGTATGGTCGCGGCACCGCTTCTGGTGCCGGAAAAAAAGTAAGCATTTTT
TTTTTCAGTTGAGATTTAGGTCACTGCGGAATCGTTTTTTATAAAT
TTTTTTTAACAACGCCAACCAGTATAAAGCCAACGCTTTTTT
ATCTTACCAACCGAGCACAAGAATTAGGCAGAGGCATTTTCGAGTTTTT
AAGTAATAATATAAAGTACCGACAGAATCGCCATATTTTTT
TTTTTCAACAGTAGGGGACGCTGAGGTCTGAGAGACTACAAATGCAA
CAGAAGGAGAAGCCCTGAAATAGCAATAGCTTGAGCGCTAATATTTTTT
TTTTTCAGAGAGATAACCGAAACGCACATATGCGGAATAAAC
TTTTTTTCAGTGAATAGAAAGATTCATTTTTT
AAAAGGTAAATTCTTACATGTAATTTGAGTTAAGCCCAATAATAATTTTT
TTTTTCCAGTAATAAGAGTCTGTCCATCCTTGATTAGGTTG
TTTTTGATAGCCGAACAAAGTTACTGATTTCAGCGGAGTAGGTAAATCATTGCCT
GTTGCGCCCGATATATCGCTTTTGCGGGATCGTCACCCTCAAAGCTGCTCATTTTT
**H1 design oligo sequences (5′-3′)**
GCAGTAGAGTAGGTAGAGATTAGGCAGGCAATTCGAACCCATCAGCATTGACGACCGC
GCAGTAGAGTAGGTAGAGATTAGGCAACGACGGCATCTCATTTTCAGGGAACACTGAG
GCAGTAGAGTAGGTAGAGATTAGGCAGGCGCTAGGGCGCATAGAGCTTGA
GCAGTAGAGTAGGTAGAGATTAGGCAAGCTCATTAGAAACCACACCAGTCAAACCGAA

GCAGTAGAGTAGGTAGAGATTAGGCACTGCTCATCGTTGAGTAACATTAT
GCAGTAGAGTAGGTAGAGATTAGGCAAATCCCCCTCAAAATAAGTTTTGCGTAAGAGC
GCAGTAGAGTAGGTAGAGATTAGGCAGCCTGTAGTGTAAACGCTAATAGTGAGGATAA
GCAGTAGAGTAGGTAGAGATTAGGCAAAGATCGCTGAGGAAGATTGTATTTGTTAA
GCAGTAGAGTAGGTAGAGATTAGGCAAATTAAAGTTTTGTCGTATTACGC

Table S12. Staple sequences used for the T3 triangle 6 folding.

**Core structure oligo sequences (5′-3′)**
CGCGCTTAATGCGCCGCTACGAATCAGAATCGGAACTACCGGGG
AGTACATATTAACCTCCGGTTTTTTAGGTTGGGTTATCGCGCA
TCCGTGAGCATTAATTGCGTTGCGGGTCACGCCGGGTTACCTGCAG
ATTAGGCAGGTCAGACGATTGGCCAAAGCCAGATAGCCCCCGTCGA
TATGAGCCGCTCACTGTCACTGCGCAGCACGCGCAACAGCTGA
ATCATTACCGATAACTCATCGAGTTTTACAAGCATATAACTA
TAACGGATTCAGATGATGTATCACGAACCGCCCACAAACGCCGCCA
GTCACCAGAGCCGCCACCATTTTTAACCACCACCAGAGCCGCC
AGAACGCGCAATCAATAATCGGCTGAACCTCCTTCATCGTAGG
CGACTTAGTACCGCAATAAAGAAATTGATTCGACTTGC
CCAAGTTTTAACGGGTATTAATTCTAAGTTTTACGCGAGGC
TGCGGCTGGTAATGGGAATTCGTAGGCCAGAATGCGTTTGAGGGTTG
CCTCAGAGACTGGATTATACTTCTTTTTGTTATCCCAATACAAAATA
CGTTCCTGGTAATATCATACCTACCCTCGTTAAGGGCGCG
ACATCGGGTACACTGGTCTTTGCTCGTCATAAAAAGAAGAAGATAAGT
GCCACCACCCTCATCAATAGAAAATTTTTTATATGGTTT
GGAAGGGCGATCGGTGCGGGCCTCTTTTTTTGCTATTACGCC
AATGGAAACTAATTCTGTCCAGATTTTTGACGACA
GAGAGAATAACATAAAAGCTGGCGATATAATTTTTAAGAAACGCAAA
TTACATTTAACAATTTCATTTGAATTTTTTTCCTTTTTT
GCGCAGTGCCCGCTTTTGAGACGGGTGCCTGTCCGGGTCCAC
TGTGTTCAGCAAATCTAACAGTGTGTTTTTATTGCAGTAAGCGTCAT
AGTTTATTTTTTTTGTCACAAGGCGATTAAGAAC
CAGAACAAACGGTACGGAGGCCACCACTCTGTCATCAGATTGTTGCCC
TAGAAGAACTCAAACCTTTGATTAAATTAACCGTTGTAGGTTAACGG
TCCTCATTTTGATATTACCCTCAGAACCAGCACCATATCTAACAGTA
AAGCCGTTTTTCGTAGATTTTCAGGTAGAAACCCTGTTTATCAACAAT
TTATTCATCAAACATCAAGAAAACAAAATTAAGCTAATGCGTTACAAA
ATAAAGGTGGCTTGGGTAACGCCACCTTTACAGGGAGGTTTTGAAGCC
CCCGTATAGGGTCAGTGCCTTGAGCAATACTTTATCGGCCTAATCAGT
AACAGGGAAGCGCATTTTTTAGACGGGAGAATTAACTGAA
GCTGGTAGGGCGAAAAACCGTCGCGCCATGAAGGAGCG
CTCCCTCAGACATAGCCCCCTTATTTTTAGCGTTTGCCA
GGAATAGGATATACAGAAAATTATTATTATTTAACGTCA
TACAATTTTATCCTGAATCTTACCTGCCAGTTCCAAATAA
CGATGGCCTTTTGGGGTCGAGGTGTCTTCGCGGTTTCTGC
ACGAGCATGTTTAACGTCGCCTGATGCTCAGTTTCGGAAC
TATCAGGGCGGGGAAAAGCACTAAGCGGGAGCAGACAGGA
GCAATTCATCAATATAGCGGAATTTGCTTTG
ATCGGCATCTATTATTCTGAAACAGAGGCGAAAATACCAA
AACGGATAGAAACGATTGAATAATGGAACCCTACTATGGTATCATCAAGAAACCA
CCGTAACACAAGCCCAATAGGAAAATCCTGATTGTTGTTGTGGAGCCGCCACGGG
GGATCCCCCCTGCGGGCCGTTTTCTGAGAAGCGTGCTTT
GTTTTAGCGTCTTTCCTTATCATTATAAACAACATGTTCA
CCGATTTAAGATGATGGGAAACAATCGGCGAAACGTACA
AAAACAGACACCCTCACGTACTCAGGAGGTTTCGGGGTTTTGCTTTG

TGATGAAATTCAATTACCTGAGCACTGCCTATACCAGGCGTACAGGAAAATAAA
TTTCGGTGTGTACTGGTAATAAGTTTGATGAGATAAGTG
GAAGCATAAAGTGTAATTGTTATCCGAGCTCGTAAAGGTT
ACATGGCTTTTTAACGAACAGTTAATGCCCCACATCCCT
TGACAGGAGGTTGTTGACGCTCAACGGAACAATATTCCTGATTTGGCAA
AAAGGGGGATGTGCTGTGCCAAGCAATAGCAGGGGTTTTC
GAGGGGTTAGAACCTACGTATAAATATAAGTGAATTTAC
ATTAGGATTAGGAGCCATTTTTTTGGGAATT
GTAAAGTTACATCCTCATATGACCAACTTTGATTTTTGCGCGTTTTA
GAACCCTTTACATTGGCAGATTTTAAAAGCCTTTGCCCGAACGTCAGCGGAT
GTCATTGCAGGCGCTTTCTTTTTCACTCAATCCGCCGGGCGCGGTTGCGG
CTTATAAACTGATTGCAAAAAAATAACAATTCGACAAACCACCACACCCG
CAACTAATCCATTGCCATTAAAAATCCTGA
CCGAACACGATTGAGGACCTGCAACGTGAGGCGGTCAGTATAGTACGGT
AAAAGCCGCACAGGCGGCCTTCAATAAGAGGATCGCGTCTAATAGGAA
CAGCGTGGTGGTCAGTCAAATGAAGAACGAA
GAAAGAGGCGGACTTGTAGAACGTAGCCTCCGAGCACTAAACATTTGA
CTTTACACCCGTAAACAAACTTAAAAAGAGACGCAGAAAGCTTTCAT
CTGGAGGTGTCCAGCATCAGCGGGATGAATCGAGCAACCGTCAGAC
CCACCAGCAGAAGATAAGCCCTAACAACAGTT
TGGTGAAGGGATAGCTTTCCAGTTATCGACATCGTTCCGGTAGATAAT
CAACATTAGGCCTTCCTGTAGCCAAAAATAATAGCCCGAAACACGACC
AGAAAGGAAGGCGGTTGTGTACTGGAACAAGAAATCGGCAAAATCC
GAATAAAAGGGACATTCTGGCCAAAAGAATACTAAAATAGAAAGGA
TGAATGGCTATTAGACCATTAGATCGCGAGCTATTCGAGCAAAAAGAT
AGATTTAGTAGAGCTTAAGAGGTCAAAGAGGA
CAAGCGCGTAAAGCTCAACATGTTGTTTCATTC
TATTACTTTTTGCCAGAGATTAGATCAATATCTGCTGGTCAGCACCGT
TGCCCTTCACCGCTTTTTTCGTCTCGTCGCTGGC
GATCCAGCCGGTGCTTTTTCCCTGCACAAGAATG
AATTGCTGCAGACGGTCCCCCAGCTAAACGGGTAAAATACGTAATGCC
TGGATTATAACAGGAAAATTTTTCGCTCATGGAA
AACATCGCTCTGACCTGAAAGCGTCAGAGATAGGATTTAGAAGTCTT
TTTGTCTTTAATGCGCGAACTGATAAACAGAGAGTGCCACGCTGAGA
AAGAAGTAAACAGTAATGTGAGCGAGTAACAACCCGTCATAACC
TTTGAGTAACATTATTTTTCATTTTGTCGTCTGAA
ACTACGAATACACTAAAACACTCTAACACCGGGATGGCT
GACACGAACTAACGGGTAAGAGCAACACTATCGGATTCTCATTGAAT
GTGTAGCGGTTTTTCACGCTGCGCCGCCAGCAGTTGGGGTTTCTCCG
CCGTGGGAACAAAGCATAAACAACATTATTACAGGT
TTGTGAGAACGTGGACTTTTTCCAACGTCAATTGCCCCAGCAG
AAATCTAAAGCATCACCTTGTTTTTTGAACCCAGCGGT
AAACAAAGATGTTACTCCCAAATCATTTTAACGCATAGG
AGAGGCTTTTGTAGTGATGAAGGCAAATATCAGGTTATC
GCCCTGAGTTTTTGAGTTGCAGCAAGCTAAGCCGGCGAACGTGGC
CCAGTCGGGAAACCTGTTTTTTGTGCCAGCTGCATTA
TTAAATATGCAACTAAATCTTTGACAATCATAAGGGAA
CGATTAATTTTTGGGATTTTTAAACATTTTTGAGGCGCCGTCAA
TAGCCGGAAGACCAGGGAACTGGCCTACGTTAATAAAGAT

CATTGTGAATTACCTTATGCGAACGTAACAAAGCTGCTTTGAGG
AGGGGGTAAAAAACCAGAACCAGAAATTGCTCCTTTTGAT
TACAACGGACTAAAGACTTTTTCATGAGGAAATTCATTA
CGAAAATCCTGCGCCAGGGTGGTTTTTTTTTCTTTTCACCAG
CGCCTGTGCGAGTAAAAGAGTAAATATTTTTTAAACCCTCAA
GAACGTGCCAAAAGAAGGCACCAACCTAAAACCGCAACCA
GCTTACGGCGGTGGTGCCATCCCAGCCAGCAGTGGCAAAT
CAAACGCGGTCCGCTGGTGGTTCCGAGTCCACTATTAAAG
AGATAGACAAGAATTTTTAGCGATTTACCAGTCCCGGAA
CTCCTGTCCATCACGCAGTAATAACATTTTTCACTTGACC
GATTGTATCGTTTGCTAAACAACTTTCAA
GGGTGAGAAAGGCCGATAGCGATTTAAACAGCTTGATAC
TCCACAGACCGATTGAATACAGGCAAGGCACCACCCTCAG
AGAGGGTAGCAGTATGTAACGGAAAGGTGAATCAAAATCACCGGAACCG
TTTTTAGAACCCTCATATATTTTAAGCCTTTATTGCCTG
AGGTCAGGAAAGGCTCTTTCACGTTGAAAATCGCGTCCAATACTCTGCC
TCATAGGTGACCACCGTAATCAGTACTGTAGCGCGTTTT
TGAGATTTTTGAGATGTCAGGACGTTGTGTCGAAATCCGTTTTTGACCTGCTCC
AGTAAAAGGTGGCATCAATTCTTTTTACTAATAGTATTAGCTATATTTT
TCTTTTCAAGCGTCAGAGCGACAGAATCAAGTGAGTCAATACTTTTTC
TAAGCAAATATTTACCCTGACTATTGTACCCCAATAATTT
CAAAAGGAGCCTTTAACGATAGTTCAAAGGCTCAAATCAC
TCAGGTCTCGTAACCGTGCATGCGGTGTAGA
TTTTAAGGGGAGCAAAATTCAAAATGCCTGAGTAATGTGTTTGCCTTT
ATGTAAATGCTGATGCCTACCTTTAATCAATATATGTGAGTGAATAAC
TCATTATTTTTTACCAGGTTTAATTCCCTGACGAGAAACACAGCGAAAG
TAATCAAAAACGTCACACCATTACTTCTGACCAGAACTGG
AGAAGGCTTATCCGGTAACCAAGTACCGCAATCCGACAAAAGGTAAAG
TGAAAGTTTTTATTAAGAGGCTGACTGAGAGA
CAGATATCACCCTGAACAAAGTCAACTGTTGGACACCAC
AAAACATTATGACACCGGAACCAGCCCTCAGAGGAAATTAAAAGGGCG
TGGGCGCACCAGCCAGCTTTCCGGTCAGGAAG
CATTAGCAATATTGACGCCAAAGAATTAGCAAAATTAAGC
AGGCCGGAATCCCTGTAATACTTTTGCGGGAGAAATGCAA
ACGGTAATGTTTATCAGGAACAACTAAAGGAACGACAGTA
CTGATAAAGCCCACGCAACCGTTCTAGCTTTTTGATAAAT
CAGTTTGATACATTCAACAGCCCTCACAACGCC
CAAAGACATTCATTAATACCCAAATAAATTTACGGAATC
GAGACAGTATCAGGTCATTTCAACCATAAAGCTAAATCGGAATAAAGC
TAACATCCTAAATTTTTTAATATTTTGTTAAATTCTGT
TTGAAAACGGGAGTTAAAGGCCGCGTCGCTGAGGCTTGCA
TGTTAAATCAGCTCATTTTTTTTTTAACCTCAGAAA
GAAAGATAAAAGTTTTTGAATTACGAGCGGCGGA
TACAAACTATAGTTAGCGTAACGATCTAAATAACCAGCGC
GGCTACAGAGGCTCATTCATTTTTTGAATAAGGCTTGTCAACTTTAA
AAATCCAAATAAAGTAAAGGCGTTAAATAAGACAAGCAAAT
TGACCGTAATGGGATAGGCAGATACATAACGCCTCATCAGT
ATTTGGGGACATTTCGCAATTTTTTGGTCAATCAATCATAGAGATCTA
CAAAGAACCCATCGATAGCAGTCCTCAAGAGAAG

GCAAGGATAAGATTCCCATTTTTTTCTGCGAACGAGTGTCTGGAA
AGTTTGAGGGGACGATTGCGAATGGTTGATATAGCATGTAACCTGT
ACCGTGTGCGCCCAATAGCAATAATTAGCAAACGTAGAAAATACATA
CCTCAGAACCGCCAAGCCACCACCAGTAGCCAATGAAAGCGAGAAA
AATATAATGCTGTTGCCGGATTTTTAGGGTAGCTATTTTTGA
AGTGAATTCTGTAAATCGTCGCTATTAATTAATATACAAAAAATATA
ATATAACAGTTAAACATCAATATGATATTCATAACCGA
TTGTACCATCGATGAAGAGTCTTGAATTTCAGCTTAGATAGAATCC
CCGGAATTTTTCAAACTCCAACTATAGTCATTTTTAAGCAAAGCGG
AATGACAACAACCATCTCCAAAAAATTAGATTTTTAGTACCTTT
ATAGTAAAATGTTTAGATTTTTTGGATATTGGGAAGATTTTTAAAT
TAAGACTCTTCCAGACGCCATTCGCCATTCAGGCTGCGC
AAGAGAATTCGCAAGAAAATACCGAGAGCCAGTATCACCGTCAC
AGCCCCAAAAACAGGAATAGAAAGCTTGCTTTCGGTTAATTTCATC
TTGTATCGCGTAAAACATCAGAAACTCAGAGGTAGCAT
AGATTTTTTTGTATCATCGCACAGCATCGGAACGAGGGTAGCAA
AGAAACAACCTGAACAAGAAATACCGCTCACAATTCCACACAAAACT
TTTCCATCCTAATTTTTAAATCATTGCACGTCCAGTCAC
GGGAATAATATCCCATACGAGCCGAACAGCCAAGATTAGTTGCTAAGTT
TAATTTTTGCACCCAGCAAAATGAATTTCAGAGGTAAAACG
TTTTACTCACATAGCTGTTTCCTGTGTGAAAAGCCTGGGCAGTTGACACCCAA
GGTGCTGCATCATGGTCCTCCTCAGTGCCTAATGAGTGAGCTATCAT
TTTGTTTCCAGAGCCTAATTAACGCTAATGAACCATACCTCACC
CCAGAATCCACGGTCACCTAAAGGATCAAGTTCACTACGCGAGCGTCTTCT
TTCTTGACAAGAACCGGATGTTTCCATGATTATAC
ATTGCATCTTCAAAGCAAATAGCGCAGATGAACCTTCATCAAT
TTACCAGACCTGGCTGACGGTGTACACGAGGCG
ACGAGAATGAATGCTTTTTTGCCAGCTCACGT
CCAACGGCTGGTCAGCGCCAACGCGCT
TAGGCGGTTTGCGTATTGGGTTTGATG
TCTTTAGGGCCAGAGCAAACGATGTCAAAAGAATT
TAGATAGGGTTGAGTGTTGCTCACGGAAATTTCTG
TTTTTATCAGAGAGATAAAGGAAACGATAAACACATGGTTTG
TTTTTGTAAATTGGGCAGGAATACCACTTTTT
ACCAGAAGCCGAAGCAATGAAATAGCAATAGATTGAGCGCTAATTTTTT
TTTCGAGAACATGTAATTTAGGCTTCTTACCAGTATTTTTT
ATGGGATGCCCACCGCTTCTGGTGCCGGAAACCAAGAAAAGTAAGTTTTT
AGAGGCATATAATTACAACAGTAGAATTGAGTTAAGCCCAATAATTTTTT
TTTTTATTCAACTAATGTCACGTTGGAATCGTCATTTTTTAATATT
TTTTTATTTAACAACGCCCCAGTAATCTTGCTTTATCAAAA
TTTTTAAAGCCAACGCTCTAGAAAAAGCCTGTTTAGTTTTTT
TTTTTAAGAGCAAGAAACCCTTTTTAGGCAAAGCGTTAGTAAATGAATT
CTATCTTAGAAACCGCCCACAAGGGCTTAATTGAGAATCGCCATTTTTT
TTTTTCAGATAGCCGAACAAAGTTCATCAGCGGAGTGAGGAAGGTAACAAGAGAA
TTTTTATCATATGCGTTTTTCCCTTTAAGACGCTGAGAAAGGTAAAG
GCGCCGACTATATTCGTTTTGCGGGATCGTCACCCTCAGCCAGAACGAGTATTTTT
**H1 design oligo sequences (5′-3′)**
GCAGTAGAGTAGGTAGAGATTAGGCACCAGAAGGCCCATGTAGCCAGCATACGGCCAC
GCAGTAGAGTAGGTAGAGATTAGGCAACGGCCAGCAATTTTCAGGGATAGCTGAGTTT

GCAGTAGAGTAGGTAGAGATTAGGCAGGCGCTAGGGCGCATCGAGCTTGA
GCAGTAGAGTAGGTAGAGATTAGGCACGCCATCATATTAATTCACCAGTCAGAATTAGA
GCAGTAGAGTAGGTAGAGATTAGGCACTCATTTGCGTACTCGTATTAAAT
GCAGTAGAGTAGGTAGAGATTAGGCACCCCCTCAACCATAATTTTTTCAAAAA
GCAGTAGAGTAGGTAGAGATTAGGCATGTAGCATATTCGCATAATAAATCGGGAGGAA
GCAGTAGAGTAGGTAGAGATTAGGCAATCGCACTTTTAAATTGTAAACG
GCAGTAGAGTAGGTAGAGATTAGGCAGGAAGTTTTGTCGTCTCTTATTAC

Supplementary Note 1

Engelhardt *et al.* [1]

M13 8064 scaffold sequence:

GGCAATGACCTGATAGCCTTTGTAGATCTCTCAAAAATAGCTACCCTCTCCGGCATTAATTTA
TCAGCTAGAACGGTTGAATATCATATTGATGGTGATTTGACTGTCTCCGGCCTTTCTCACCCT
TTTGAATCTTTACCTACACATTACTCAGGCATTGCATTTAAAATATATGAGGGTTCTAAAAAT
TTTTATCCTTGCGTTGAAATAAAGGCTTCTCCCGCAAAAGTATTACAGGGTCATAATGTTTTT
GGTACAACCGATTTAGCTTTATGCTCTGAGGCTTTATTGCTTAATTTTGCTAATTCTTTGCCTT
GCCTGTATGATTTATTGGATGTTAATGCTACTACTATTAGTAGAATTGATGCCACCTTTTCAG
CTCGCGCCCCAAATGAAAATATAGCTAAACAGGTTATTGACCATTTGCGAAATGTATCTAAT
GGTCAAACTAAATCTACTCGTTCGCAGAATTGGGAATCAACTGTTATATGGAATGAAACTTC
CAGACACCGTACTTTAGTTGCATATTTAAAACATGTTGAGCTACAGCATTATATTCAGCAATT
AAGCTCTAAGCCATCCGCAAAAATGACCTCTTATCAAAAGGAGCAATTAAAGGTACTCTCTA
ATCCTGACCTGTTGGAGTTTGCTTCCGGTCTGGTTCGCTTTGAAGCTCGAATTAAAACGCGAT
ATTTGAAGTCTTTCGGGCTTCCTCTTAATCTTTTTGATGCAATCCGCTTTGCTTCTGACTATAA
TAGTCAGGGTAAAGACCTGATTTTTGATTTATGGTCATTCTCGTTTTCTGAACTGTTTAAAGC
ATTTGAGGGGGATTCAATGAATATTTATGACGATTCCGCAGTATTGGACGCTATCCAGTCTA
AACATTTTACTATTACCCCCTCTGGCAAAACTTCTTTTGCAAAAGCCTCTCGCTATTTTGGTTT
TTATCGTCGTCTGGTAAACGAGGGTTATGATAGTGTTGCTCTTACTATGCCTCGTAATTCCTT
TTGGCGTTATGTATCTGCATTAGTTGAATGTGGTATTCCTAAATCTCAACTGATGAATCTTTC
TACCTGTAATAATGTTGTTCCGTTAGTTCGTTTTATTAACGTAGATTTTTCTTCCCAACGTCCT
GACTGGTATAATGAGCCAGTTCTTAAAATCGCATAAGGTAATTCACAATGATTAAAGTTGAA
ATTAAACCATCTCAAGCCCAATTTACTACTCGTTCTGGTGTTTCTCGTCAGGGCAAGCCTTAT
TCACTGAATGAGCAGCTTTGTTACGTTGATTTGGGTAATGAATATCCGGTTCTTGTCAAGATT
ACTCTTGATGAAGGTCAGCCAGCCTATGCGCCTGGTCTGTACACCGTTCATCTGTCCTCTTTC
AAAGTTGGTCAGTTCGGTTCCCTTATGATTGACCGTCTGCGCCTCGTTCCGGCTAAGTAACAT
GGAGCAGGTCGCGGATTTCGACACAATTTATCAGGCGATGATACAAATCTCCGTTGTACTTT
GTTTCGCGCTTGGTATAATCGCTGGGGGTCAAAGATGAGTGTTTTAGTGTATTCTTTTGCCTC
TTTCGTTTTAGGTTGGTGCCTTCGTAGTGGCATTACGTATTTTACCCGTTTAATGGAAACTTC
CTCATGAAAAAGTCTTTAGTCCTCAAAGCCTCTGTAGCCGTTGCTACCCTCGTTCCGATGCTG
TCTTTCGCTGCTGAGGGTGACGATCCCGCAAAAGCGGCCTTTAACTCCCTGCAAGCCTCAGC
GACCGAATATATCGGTTATGCGTGGGCGATGGTTGTTGTCATTGTCGGCGCAACTATCGGTA
TCAAGCTGTTTAAGAAATTCACCTCGAAAGCAAGCTGATAAACCGATACAATTAAAGGCTCC
TTTTGGAGCCTTTTTTTTGGAGATTTTCAACGTGAAAAAATTATTATTCGCAATTCCTTTAGTT
GTTCCTTTCTATTCTCACTCCGCTGAAACTGTTGAAAGTTGTTTAGCAAAATCCCATACAGAA
AATTCATTTACTAACGTCTGGAAAGACGACAAAACTTTAGATCGTTACGCTAACTATGAGGG
CTGTCTGTGGAATGCTACAGGCGTTGTAGTTTGTACTGGTGACGAAACTCAGTGTTACGGTA
CATGGGTTCCTATTGGGCTTGCTATCCCTGAAAATGAGGGTGGTGGCTCTGAGGGTGGCGGT
TCTGAGGGTGGCGGTTCTGAGGGTGGCGGTACTAAACCTCCTGAGTACGGTGATACACCTAT
TCCGGGCTATACTTATATCAACCCTCTCGACGGCACTTATCCGCCTGGTACTGAGCAAAACC
CCGCTAATCCTAATCCTTCTCTTGAGGAGTCTCAGCCTCTTAATACTTTCATGTTTCAGAATA
ATAGGTTCCGAAATAGGCAGGGGGCATTAACTGTTTATACGGGCACTGTTACTCAAGGCACT
GACCCCGTTAAAACTTATTACCAGTACACTCCTGTATCATCAAAAGCCATGTATGACGCTTA

CTGGAACGGTAAATTCAGAGACTGCGCTTTCCATTCTGGCTTTAATGAGGATTTATTTGTTTG
TGAATATCAAGGCCAATCGTCTGACCTGCCTCAACCTCCTGTCAATGCTGGCGGCGGCTCTG
GTGGTGGTTCTGGTGGCGGCTCTGAGGGTGGTGGCTCTGAGGGTGGCGGTTCTGAGGGTGGC
GGCTCTGAGGGAGGCGGTTCCGGTGGTGGCTCTGGTTCCGGTGATTTTGATTATGAAAAGAT
GGCAAACGCTAATAAGGGGGCTATGACCGAAAATGCCGATGAAAACGCGCTACAGTCTGAC
GCTAAAGGCAAACTTGATTCTGTCGCTACTGATTACGGTGCTGCTATCGATGGTTTCATTGGT
GACGTTTCCGGCCTTGCTAATGGTAATGGTGCTACTGGTGATTTTGCTGGCTCTAATTCCCAA
ATGGCTCAAGTCGGTGACGGTGATAATTCACCTTTAATGAATAATTTCCGTCAATATTTACCT
TCCCTCCCTCAATCGGTTGAATGTCGCCCTTTTGTCTTTGGCGCTGGTAAACCATATGAATTT
TCTATTGATTGTGACAAAATAAACTTATTCCGTGGTGTCTTTGCGTTTCTTTTATATGTTGCCA
CCTTTATGTATGTATTTTCTACGTTTGCTAACATACTGCGTAATAAGGAGTCTTAATCATGCC
AGTTCTTTTGGGTATTCCGTTATTATTGCGTTTCCTCGGTTTCCTTCTGGTAACTTTGTTCGGC
TATCTGCTTACTTTTCTTAAAAAGGGCTTCGGTAAGATAGCTATTGCTATTTCATTGTTTCTTG
CTCTTATTATTGGGCTTAACTCAATTCTTGTGGGTTATCTCTCTGATATTAGCGCTCAATTACC
CTCTGACTTTGTTCAGGGTGTTCAGTTAATTCTCCCGTCTAATGCGCTTCCCTGTTTTTATGTT
ATTCTCTCTGTAAAGGCTGCTATTTTCATTTTTGACGTTAAACAAAAAATCGTTTCTTATTTG
GATTGGGATAAATAATATGGCTGTTTATTTTGTAACTGGCAAATTAGGCTCTGGAAAGACGC
TCGTTAGCGTTGGTAAGATTCAGGATAAAATTGTAGCTGGGTGCAAAATAGCAACTAATCTT
GATTTAAGGCTTCAAAACCTCCCGCAAGTCGGGAGGTTCGCTAAAACGCCTCGCGTTCTTAG
AATACCGGATAAGCCTTCTATATCTGATTTGCTTGCTATTGGGCGCGGTAATGATTCCTACGA
TGAAAATAAAAACGGCTTGCTTGTTCTCGATGAGTGCGGTACTTGGTTTAATACCCGTTCTTG
GAATGATAAGGAAAGACAGCCGATTATTGATTGGTTTCTACATGCTCGTAAATTAGGATGGG
ATATTATTTTTCTTGTTCAGGACTTATCTATTGTTGATAAACAGGCGCGTTCTGCATTAGCTG
AACATGTTGTTTATTGTCGTCGTCTGGACAGAATTACTTTACCTTTTGTCGGTACTTTATATTC
TCTTATTACTGGCTCGAAAATGCCTCTGCCTAAATTACATGTTGGCGTTGTTAAATATGGCGA
TTCTCAATTAAGCCCTACTGTTGAGCGTTGGCTTTATACTGGTAAGAATTTGTATAACGCATA
TGATACTAAACAGGCTTTTTCTAGTAATTATGATTCCGGTGTTTATTCTTATTTAACGCCTTAT
TTATCACACGGTCGGTATTTCAAACCATTAAATTTAGGTCAGAAGATGAAATTAACTAAAAT
ATATTTGAAAAAGTTTTCTCGCGTTCTTTGTCTTGCGATTGGATTTGCATCAGCATTTACATA
TAGTTATATAACCCAACCTAAGCCGGAGGTTAAAAAGGTAGTCTCTCAGACCTATGATTTTG
ATAAATTCACTATTGACTCTTCTCAGCGTCTTAATCTAAGCTATCGCTATGTTTTCAAGGATT
CTAAGGGAAAATTAATTAATAGCGACGATTTACAGAAGCAAGGTTATTCACTCACATATATT
GATTTATGTACTGTTTCCATTAAAAAAGGTAATTCAAATGAAATTGTTAAATGTAATTAATTT
TGTTTTCTTGATGTTTGTTTCATCATCTTCTTTTGCTCAGGTAATTGAAATGAATAATTCGCCT
CTGCGCGATTTTGTAACTTGGTATTCAAAGCAATCAGGCGAATCCGTTATTGTTTCTCCCGAT
GTAAAAGGTACTGTTACTGTATATTCATCTGACGTTAAACCTGAAAATCTACGCAATTTCTTT
ATTTCTGTTTTACGTGCAAATAATTTTGATATGGTAGGTTCTAACCCTTCCATTATTCAGAAG
TATAATCCAAACAATCAGGATTATATTGATGAATTGCCATCATCTGATAATCAGGAATATGA
TGATAATTCCGCTCCTTCTGGTGGTTTCTTTGTTCCGCAAAATGATAATGTTACTCAAACTTTT
AAAATTAATAACGTTCGGGCAAAGGATTTAATACGAGTTGTCGAATTGTTTGTAAAGTCTAA
TACTTCTAAATCCTCAAATGTATTATCTATTGACGGCTCTAATCTATTAGTTGTTAGTGCTCCT
AAAGATATTTTAGATAACCTTCCTCAATTCCTTTCAACTGTTGATTTGCCAACTGACCAGATA
TTGATTGAGGGTTTGATATTTGAGGTTCAGCAAGGTGATGCTTTAGATTTTTCATTTGCTGCT
GGCTCTCAGCGTGGCACTGTTGCAGGCGGTGTTAATACTGACCGCCTCACCTCTGTTTTATCT

TCTGCTGGTGGTTCGTTCGGTATTTTTAATGGCGATGTTTTAGGGCTATCAGTTCGCGCATTA
AAGACTAATAGCCATTCAAAAATATTGTCTGTGCCACGTATTCTTACGCTTTCAGGTCAGAA
GGGTTCTATCTCTGTTGGCCAGAATGTCCCTTTTATTACTGGTCGTGTGACTGGTGAATCTGC
CAATGTAAATAATCCATTTCAGACGATTGAGCGTCAAAATGTAGGTATTTCCATGAGCGTTT
TTCCTGTTGCAATGGCTGGCGGTAATATTGTTCTGGATATTACCAGCAAGGCCGATAGTTTG
AGTTCTTCTACTCAGGCAAGTGATGTTATTACTAATCAAAGAAGTATTGCTACAACGGTTAA
TTTGCGTGATGGACAGACTCTTTTACTCGGTGGCCTCACTGATTATAAAAACACTTCTCAGGA
TTCTGGCGTACCGTTCCTGTCTAAAATCCCTTTAATCGGCCTCCTGTTTAGCTCCCGCTCTGAT
TCTAACGAGGAAAGCACGTTATACGTGCTCGTCAAAGCAACCATAGTACGCGCCCTGTAGCG
GCGCATTAAGCGCGGCGGGTGTGGTGGTTACGCGCAGCGTGACCGCTACACTTGCCAGCGCC
CTAGCGCCCGCTCCTTTCGCTTTCTTCCCTTCCTTTCTCGCCACGTTCGCCGGCTTTCCCCGTC
AAGCTCTAAATCGGGGGCTCCCTTTAGGGTTCCGATTTAGTGCTTTACGGCACCTCGACCCC
AAAAAACTTGATTTGGGTGATGGTTCACGTAGTGGGCCATCGCCCTGATAGACGGTTTTTCG
CCCTTTGACGTTGGAGTCCACGTTCTTTAATAGTGGACTCTTGTTCCAAACTGGAACAACACT
CAACCCTATCTCGGGCTATTCTTTTGATTTATAAGGGATTTTGCCGATTTCGGAACCACCATC
AAACAGGATTTTCGCCTGCTGGGGCAAACCAGCGTGGACCGCTTGCTGCAACTCTCTCAGGG
CCAGGCGGTGAAGGGCAATCAGCTGTTGCCCGTCTCACTGGTGAAAAGAAAAACCACCCTG
GCGCCCAATACGCAAACCGCCTCTCCCCGCGCGTTGGCCGATTCATTAATGCAGCTGGCACG
ACAGGTTTCCCGACTGGAAAGCGGGCAGTGAGCGCAACGCAATTAATGTGAGTTAGCTCAC
TCATTAGGCACCCCAGGCTTTACACTTTATGCTTCCGGCTCGTATGTTGTGTGGAATTGTGAG
CGGATAACAATTTCACACAGGAAACAGCTATGACCATGATTACGAATTCGAGCTCGGTACCC
GGGGATCCTCAACTGTGAGGAGGCTCACGGACGCGAAGAACAGGCACGCGTGCTGGCAGAA
ACCCCCGGTATGACCGTGAAAACGGCCCGCCGCATTCTGGCCGCAGCACCACAGAGTGCAC
AGGCGCGCAGTGACACTGCGCTGGATCGTCTGATGCAGGGGGCACCGGCACCGCTGGCTGC
AGGTAACCCGGCATCTGATGCCGTTAACGATTTGCTGAACACACCAGTGTAAGGGATGTTTA
TGACGAGCAAAGAAACCTTTACCCATTACCAGCCGCAGGGCAACAGTGACCCGGCTCATAC
CGCAACCGCGCCCGGCGGATTGAGTGCGAAAGCGCCTGCAATGACCCCGCTGATGCTGGAC
ACCTCCAGCCGTAAGCTGGTTGCGTGGGATGGCACCACCGACGGTGCTGCCGTTGGCATTCT
TGCGGTTGCTGCTGACCAGACCAGCACCACGCTGACGTTCTACAAGTCCGGCACGTTCCGTT
ATGAGGATGTGCTCTGGCCGGAGGCTGCCAGCGACGAGACGAAAAAACGGACCGCGTTTGC
CGGAACGGCAATCAGCATCGTTTAACTTTACCCTTCATCACTAAAGGCCGCCTGTGCGGCTT
TTTTTACGGGATTTTTTTATGTCGATGTACACAACCGCCCAACTGCTGGCGGCAAATGAGCA
GAAATTTAAGTTTGATCCGCTGTTTCTGCGTCTCTTTTTCCGTGAGAGCTATCCCTTCACCAC
GGAGAAAGTCTATCTCTCACAAATTCCGGGACTGGTAAACATGGCGCTGTACGTTTCGCCGA
TTGTTTCCGGTGAGGTTATCCGTTCCCGTGGCGGCTCCACCTCTGAAAGCTTGGCACTGGCCG
TCGTTTTACAACGTCGTGACTGGGAAAACCCTGGCGTTACCCAACTTAATCGCCTTGCAGCA
CATCCCCCTTTCGCCAGCTGGCGTAATAGCGAAGAGGCCCGCACCGATCGCCCTTCCCAACA
GTTGCGCAGCCTGAATGGCGAATGGCGCTTTGCCTGGTTTCCGGCACCAGAAGCGGTGCCGG
AAAGCTGGCTGGAGTGCGATCTTCCTGAGGCCGATACTGTCGTCGTCCCCTCAAACTGGCAG
ATGCACGGTTACGATGCGCCCATCTACACCAACGTGACCTATCCCATTACGGTCAATCCGCC
GTTTGTTCCCACGGAGAATCCGACGGGTTGTTACTCGCTCACATTTAATGTTGATGAAAGCT
GGCTACAGGAAGGCCAGACGCGAATTATTTTTGATGGCGTTCCTATTGGTTAAAAAATGAGC
TGATTTAACAAAAATTTAATGCGAATTTTAACAAAATATTAACGTTTACAATTTAAATATTTG

CTTATACAATCTTCCTGTTTTTGGGGCTTTTCTGATTATCAACCGGGGTACATATGATTGACA
TGCTAGTTTTACGATTACCGTTCATCGATTCTCTTGTTTGCTCCAGACTCTCA

References

1. Engelhardt, F. A. S. *et al.* Custom-Size, Functional, and Durable DNA Origami with Design-Specific Scaffolds. *ACS Nano* **13**, 5015–5027 (2019).
2. Gerling, T., Kube, M., Kick, B. & Dietz, H. Sequence-programmable covalent bonding of designed DNA assemblies. *Sci. Adv.* **4**, eaau1157 (2018).
3. Yu, I.-M. *et al.* Structure of the Immature Dengue Virus at Low pH Primes Proteolytic Maturation. *Science* **319**, 1834–1837 (2008).
4. Yu, I.-M. *et al.* Association of the pr Peptides with Dengue Virus at Acidic pH Blocks Membrane Fusion. *J. Virol.* **83**, 12101–12107 (2009).
5. Kremer, J. R., Mastronarde, D. N. & McIntosh, J. R. Computer Visualization of Three-Dimensional Image Data Using IMOD. *J. Struct. Biol.* **116**, 71–76 (1996).
6. Zivanov, J. *et al.* New tools for automated high-resolution cryo-EM structure determination in RELION-3. *eLife* **7**, e42166 (2018).
7. Zheng, S. Q. *et al.* MotionCor2: anisotropic correction of beam-induced motion for improved cryo-electron microscopy. *Nat. Methods* **14**, 331–332 (2017).
8. Rohou, A. & Grigorieff, N. CTFFIND4: Fast and accurate defocus estimation from electron micrographs. *J. Struct. Biol.* **192**, 216–221 (2015).
9. Wagner, T. *et al.* SPHIRE-crYOLO is a fast and accurate fully automated particle picker for cryo-EM. *Commun. Biol.* **2**, 1–13 (2019).
